# A Co-Expression Network in Hexaploid Wheat Reveals Mostly Balanced Expression and Lack of Significant Gene Loss of Homeologous Meiotic Genes Upon Polyploidization

**DOI:** 10.3389/fpls.2019.01325

**Published:** 2019-10-18

**Authors:** Abdul Kader Alabdullah, Philippa Borrill, Azahara C. Martin, Ricardo H. Ramirez-Gonzalez, Keywan Hassani-Pak, Cristobal Uauy, Peter Shaw, Graham Moore

**Affiliations:** ^1^John Innes Centre, Norwich Research Park, Norwich, United Kingdom; ^2^School of Biosciences, University of Birmingham, Birmingham, United Kingdom; ^3^Computational and Analytical Sciences, Rothamsted Research, Harpenden, United Kingdom

**Keywords:** wheat, meiosis, hexaploid, polyploidization, co-expression, network

## Abstract

Polyploidization has played an important role in plant evolution. However, upon polyploidization, the process of meiosis must adapt to ensure the proper segregation of increased numbers of chromosomes to produce balanced gametes. It has been suggested that meiotic gene (MG) duplicates return to a single copy following whole genome duplication to stabilize the polyploid genome. Therefore, upon the polyploidization of wheat, a hexaploid species with three related (homeologous) genomes, the stabilization process may have involved rapid changes in content and expression of MGs on homeologous chromosomes (homeologs). To examine this hypothesis, sets of candidate MGs were identified in wheat using co-expression network analysis and orthology informed approaches. In total, 130 RNA-Seq samples from a range of tissues including wheat meiotic anthers were used to define co-expressed modules of genes. Three modules were significantly correlated with meiotic tissue samples but not with other tissue types. These modules were enriched for GO terms related to cell cycle, DNA replication, and chromatin modification and contained orthologs of known MGs. Overall, 74.4% of genes within these meiosis-related modules had three homeologous copies which was similar to other tissue-related modules. Amongst wheat MGs identified by orthology, rather than co-expression, the majority (93.7%) were either retained in hexaploid wheat at the same number of copies (78.4%) or increased in copy number (15.3%) compared to ancestral wheat species. Furthermore, genes within meiosis-related modules showed more balanced expression levels between homeologs than genes in non-meiosis-related modules. Taken together, our results do not support extensive gene loss nor changes in homeolog expression of MGs upon wheat polyploidization. The construction of the MG co-expression network allowed identification of hub genes and provided key targets for future studies.

## Introduction

Meiosis is a specialized mode of cell division which generates haploid gametes. Prior to meiosis, chromosomes are replicated. On entry into meiosis, homologous chromosomes (homologs) locate each other and intimately align (synapse) along their length. Within this paired structure, chromosomes recombine and crossover before being accurately segregated (Kleckner, 1996; Mercier et al., 2015; Zickler and Kleckner, 2015). This complex and dynamic process is essential to maintain genome stability and integrity over sexual life cycles and to generate genome variation, which is a major evolutionary driving force (Capilla et al., 2016; Melamed-Bessudo et al., 2016). The genetic variation created by meiotic recombination underpins plant breeding to improve crop species (Wijnker and de Jong, 2008; Choi, 2017; Fernandes et al., 2018). Polyploidization has played an important role in the evolution and speciation of flowering plants (Comai, 2005; Alix et al., 2017), although the resultant multiplicity of related genomes poses a major challenge for the meiotic process. Segregation of the chromosomes to produce balanced gametes requires correct pairing, synapsis, and recombination between only true homologs, rather than any of the other highly related chromosomes (homeologs) (Ramsey and Schemske, 2002; Comai, 2005; Stenberg and Saura, 2013).

In the last two decades, there have been significant advances in our understanding of plant meiosis. Since the isolation of the ﬁrst meiosis-speciﬁc cDNA from lily in the mid-1990s (Kobayashi et al., 1993; Kobayashi et al., 1994), more than 110 plant meiotic genes (MGs) have been identified, mainly from studies of the model diploid plants *Arabidopsis* and rice (Mercier and Grelon, 2008; Luo et al., 2014; Mercier et al., 2015). Although 25–30% of flowering plants are extant polyploids (Alix et al., 2017), the meiotic mechanisms responsible for their stabilization remain poorly understood. An exception is hexaploid wheat (*Triticum aestivum* L.), where there is now better understanding of these processes (Blary and Jenczewski, 2019). Despite possessing multiple related genomes, durum wheat, a tetraploid (AABB), and bread wheat, a hexaploid (AABBDD) behave as diploids during meiosis. Thus, most of the meiotic studies conducted in hexaploid wheat have focused on providing better understanding of the meiotic processes required to stabilize this polyploid species (Riley and Chapman, 1958; Riley, 1960; Holm, 1986; Martinez-Perez et al., 2000; Martinez-Perez et al., 2003). An emphasis has been to characterize the role of the *Ph1* locus in the suppression of recombination between homeologs (Riley et al., 1968; Holm, 1988; Holm and Wang, 1988; Feldman, 1993; Aragon-Alcaide et al., 1997; Martinez-Perez et al., 2001; Prieto et al., 2004; Colas et al., 2008; Martín et al., 2017). Recent studies have defined this phenotype to a *ZIP4* gene which duplicated and diverged on polyploidization (Martín et al., 2017; Rey et al., 2017; Martín et al., 2018; Rey et al., 2018). This event resulted in the suppression of homeologous crossover, and promotion of homologous synapsis.

Although all flowering plants have undergone at least one event of whole genome duplication during their evolutionary history (Jiao et al., 2011), it has been suggested that MG duplicates return to a single copy following whole genome duplication, more rapidly than the genome-wide average (Lloyd et al., 2014). Therefore, it has been assumed that the stabilization process upon the polyploidization of wheat also involved rapid changes in the content and expression of the genes on homeologs. This process would facilitate the correct pairing and synapsis of homeologs during meiosis. The recent development of an expression atlas for hexaploid wheat revealed that 70% of homeologous genes in syntenic triads showed balanced expression (Ramírez-González et al., 2018); however, this study did not include analysis of the genes expressed during meiosis.

Here, we assessed whether the level of expression of all genes in triads was balanced between homeologs during meiosis. Analysis indicated similar balanced expression to that observed in other wheat tissues. However, it could be argued that only meiotic specific genes might show differential expression between homeologs. Sets of candidate MGs were identified using co-expression network analysis and orthology informed approaches, allowing us to evaluate the effect of polyploidization on wheat MG copy number and expression. The combination of co-expression network analysis, in conjunction with orthologue information, will now contribute to the discovery of new MGs and greatly empower reverse genetics approaches (such as wheat TILLING and CRISPR) that can be used to validate the function of the identified candidate genes in wheat.

## Results and Discussion

An initial assessment of the homeolog expression pattern in triads during meiosis in hexaploid wheat was undertaken. Relative expression abundance of 19,801 triads (59,403 genes) was calculated for 8 tissues, including meiotic anther tissue, according to published criteria (Ramírez-González et al., 2018). This analysis revealed that the percentage of balanced triads was slightly higher in meiotic anther tissue (77.3%) than in other type of tissues (ranging from 67.3% in floral organs and 76.6% in leaves) (**Figure S1**). The copy number of genes expressed during meiosis was also investigated. This involved the definition of 19,801 triads (59,403 genes), 7,565 duplets (15,130 genes), 15,109 monads (single-copy genes), and 18,250 genes from the “others” group with various copy numbers, based on the *Ensembl* Plants database for the high confidence (HC) genes of hexaploid wheat (International Wheat Genome Sequencing Consortium, 2018) (IWGSC v1.1 gene annotation; **Table S1**). Comparison of copy number of genes expressed in the eight different tissues showed that 70.9% of the genes expressed during meiosis belonged to triads. This percentage ranged between 66.5 and 72.5% for the genes expressed in floral organs and stem tissues, respectively (**Figure S2**). The overall results were consistent with a previous study, which analyzed a range of tissues, but not meiotic anther tissue, and reported significant balanced expression between homeologous genes in these tissues (Ramírez-González et al., 2018). However, our analysis revealed a slightly higher percentage of balanced triads in meiotic anther tissue than in other types of tissues. These observations did not support the hypothesis that stabilization of polyploidization in wheat involved significant changes in gene content and expression between homeologs (Freeling, 2009; Lloyd et al., 2014; Edger et al., 2017). Considering that not all genes expressed in meiotic anther tissue are directly involved in the meiosis process, it is possible that meiotic specific genes exhibit a different pattern. Therefore, a co-expression gene network was developed to compare the expression pattern of homeologous genes in meiosis-related modules, which potentially represent meiosis specific genes, and other tissue-related modules.

### Weighted Co-Expression Network Construction

**Figure 1:**
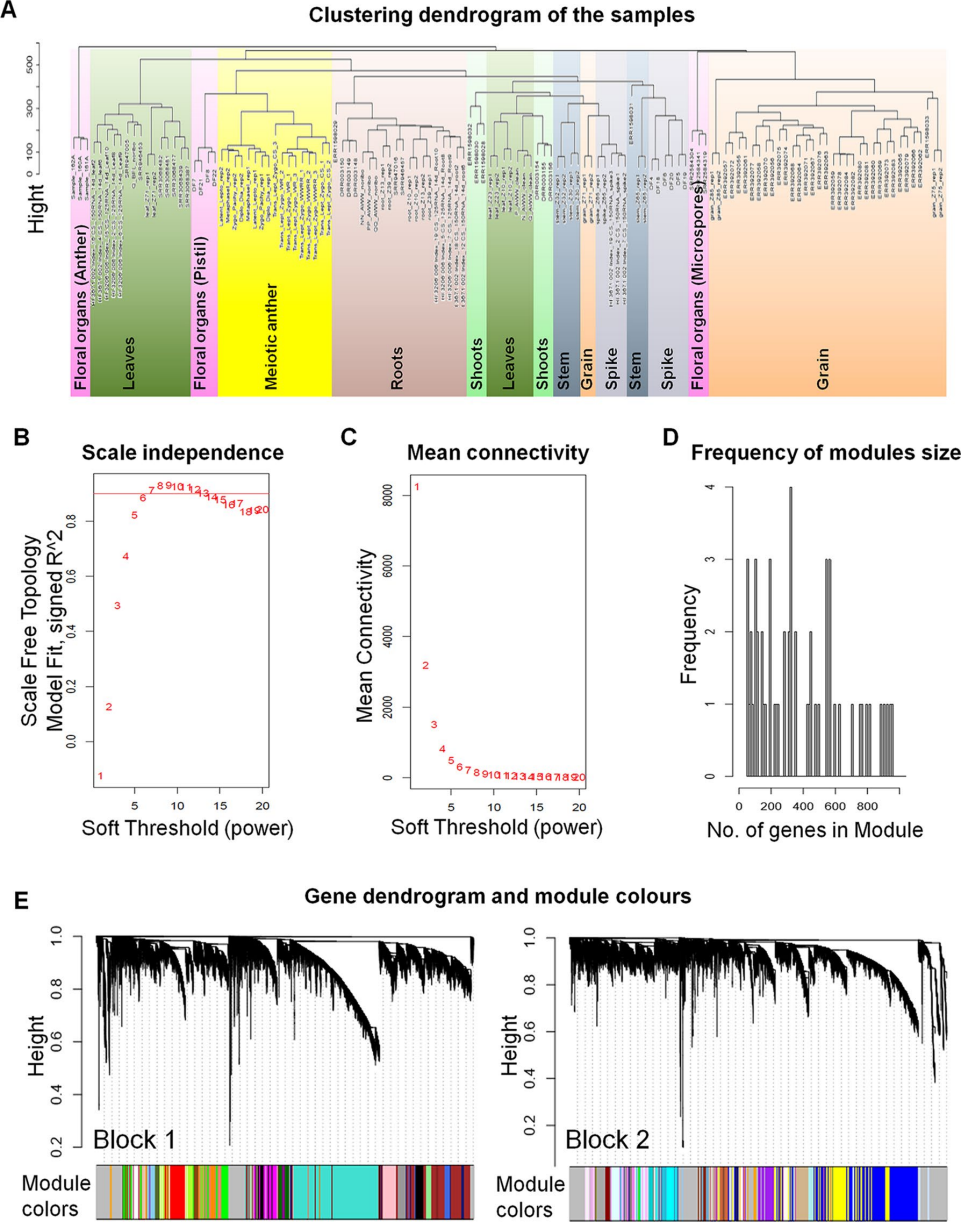
The weighted gene co-expression networks analysis (WGCNA). **(A)** Clustering dendrogram of 130 samples from different types of tissues. The sample clustering was based on the expression data of the genes expressed during meiosis. **(B)** Analysis of the scale-free fit index for various soft-thresholding powers (β). A scale-free network is a network with the property that the number of connections *k* originating from a given node exhibits a power law distribution *P* (*k*) ∼ *k* *^−^* ^ɤ^ (where 2 < ɤ < 3). The red line indicates the scale-free topology fit index (of 0.9) for which the network obeys scale free network property. The soft power threshold used in constructing the weighted gene co-expression networks was chosen as the first power to exceed the red line (then β = 7). **(C)** Analysis of the mean connectivity for various soft-thresholding powers. **(D)** Number of genes in the modules with their frequency. **(E)** Dendrogram of the analyzed genes (60,379 genes) clustered based on a dissimilarity measure of topological overlap matrices (1-TOM). Blockwise dendrogram was obtained using average linkage hierarchical clustering with maximum block size of 46,000 genes. Modules were identified using height cut off equal to 0.15 with minimum module size of 30 genes. The different color modules correspond to the branches of the dendrogram.

**Figure 2:**
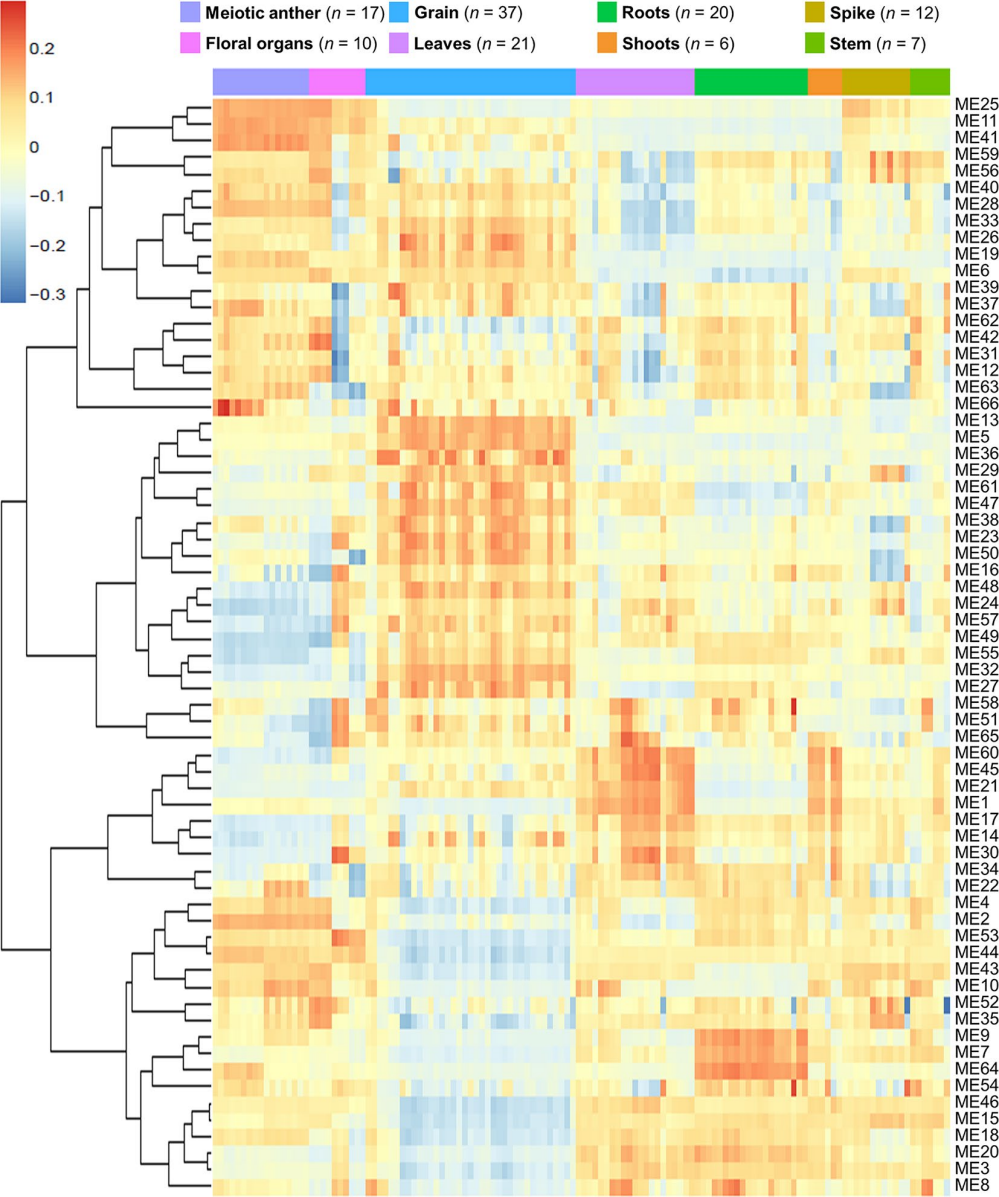
Heatmap plotting of MEs values in relationship to tissue samples. *n* indicates number of samples.

Network-based approaches have been proved useful in systems biology, to mine gene function from high-throughput gene expression data. Gene co-expression analysis has become a powerful tool to build transcriptional networks of genes involved in common biological events in plants (Usadel et al., 2009; He and Maslov, 2016; Krishnan et al., 2017; Ma et al., 2018; Yu et al., 2018; Liu et al., 2019). The use of co-expression networks has uncovered candidate genes to regulate biological processes in many plants including wheat (International Wheat Genome Sequencing Consortium, 2018), rice (Aya et al., 2011; Tan et al., 2017), and *Arabidopsis thaliana* (Costa et al., 2015; Silva et al., 2016). The recently published high-quality genome reference sequence (International Wheat Genome Sequencing Consortium, 2018) and a developmental gene expression atlas (Borrill et al., 2016; Ramírez-González et al., 2018), together with the gene expression data collected from meiotic samples, were used to build a co-expression gene network. One hundred and thirty samples from different tissue types were included in this co-expression analysis (**Table S2**; **Figure 1A**). A set of 60,379 genes out of the total 107,892 HC genes was considered expressed during meiosis [transcript per million (TPM) > 0.5 in at least one meiosis sample (biological replicate)] and used to run the co-expression analysis. Using the “WGCNA” package in R (Langfelder and Horvath, 2008; Langfelder and Horvath, 2012), genes with similar expression patterns were grouped into modules *via* the average linkage hierarchical clustering of normalized count expression values (**Figure 1E**). The power of β = 7 (scale free topology R^2^ = 0.91) was selected as the soft threshold power to emphasize strong correlations between genes and penalize weak correlations to ensure a scale-free network (**Figures 1B, C**). Based on this analysis, 50,387 out of 60,379 genes (83.5% of expressed genes) could be assigned to 66 modules. Module size ranged from 52 to 7,541 genes (mean 763 genes; median 429 genes) (**Figure 1D**). The expression patterns of all genes within a single module were summarized into a module eigengene (ME; representative gene of the module) to minimize data size for subsequent analyses. Expression patterns of modules are shown as a heatmap by plotting ME values in relation to tissue samples (**Figure 2**).

### Identification of Meiosis-Related Modules

**Figure 3:**
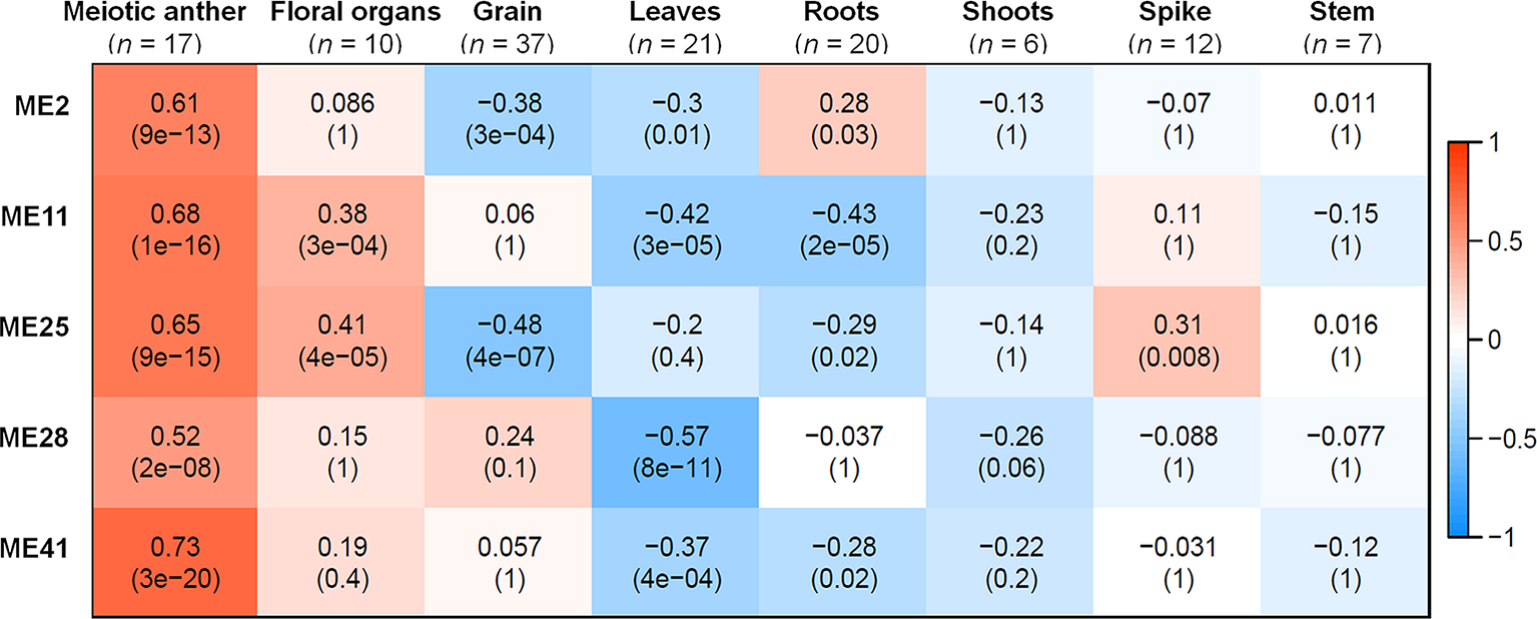
Co-expression network modules in relationship to tissues samples. Each row corresponds to a module; each column corresponds to a tissue type. Each cell contains the correlation value and, in parentheses, its corresponding FDR adjusted *P* value. *n* indicates number of samples. Only modules that have correlation value > 0.5 with meiotic anther tissue are shown.

A correlation analysis was conducted using the 66 MEs and the 8 different tissue types. A module was considered as meiosis-related when there was a strong correlation (*r*) with the 17 meiosis samples, and a weak or negative correlation with other tissue types. Accordingly, three meiosis-related modules were identified: module 2 (containing 4,940 genes), module 28 (544 genes), and module 41 (313 genes). Module 41 showed the strongest correlation with meiotic tissue (*r* = 0.73, FDR = 2.7 x 10^−20^), compared to module 2 (*r* = 0.61, FDR = 9.2 x 10^−13^) and module 28 (*r* = 0.52, FDR = 2.1 x 10^−8^) (**Figure 3**).

Two other modules (modules 11 and 25) also showed significantly positive correlation with meiotic samples (*r* = 0.68 and 0.65, respectively); however, they were not considered meiosis-related because they also correlated with samples from floral organs (at stages other than meiosis) and spike tissues, as shown in **Figure 3**. Therefore, our analysis focused on the three modules (2, 28, and 41), exhibiting a strong correlation with meiotic tissues and not with other floral organs, while modules 11 and 25 were considered as non-meiosis specific modules (referred to in this paper as non-meiotic modules). Other tissue-related modules (the top three correlated modules) were also identified to be used as controls for the meiosis-related modules in the subsequent analysis. These modules were grain-related modules 5, 13, and 32 (*r* = 0.89, 0.89, and 0.85, respectively); leaves-related modules 1, 45, and 60 (*r* = 0.72, 0.68, and 0.71, respectively); and roots-related modules 7, 9, and 64 (*r* = 0.70, 0.76, and 0.85, respectively) (**Figure S3**).

### Biological Significance of Expression Similarity in Modules

**Figure 4:**
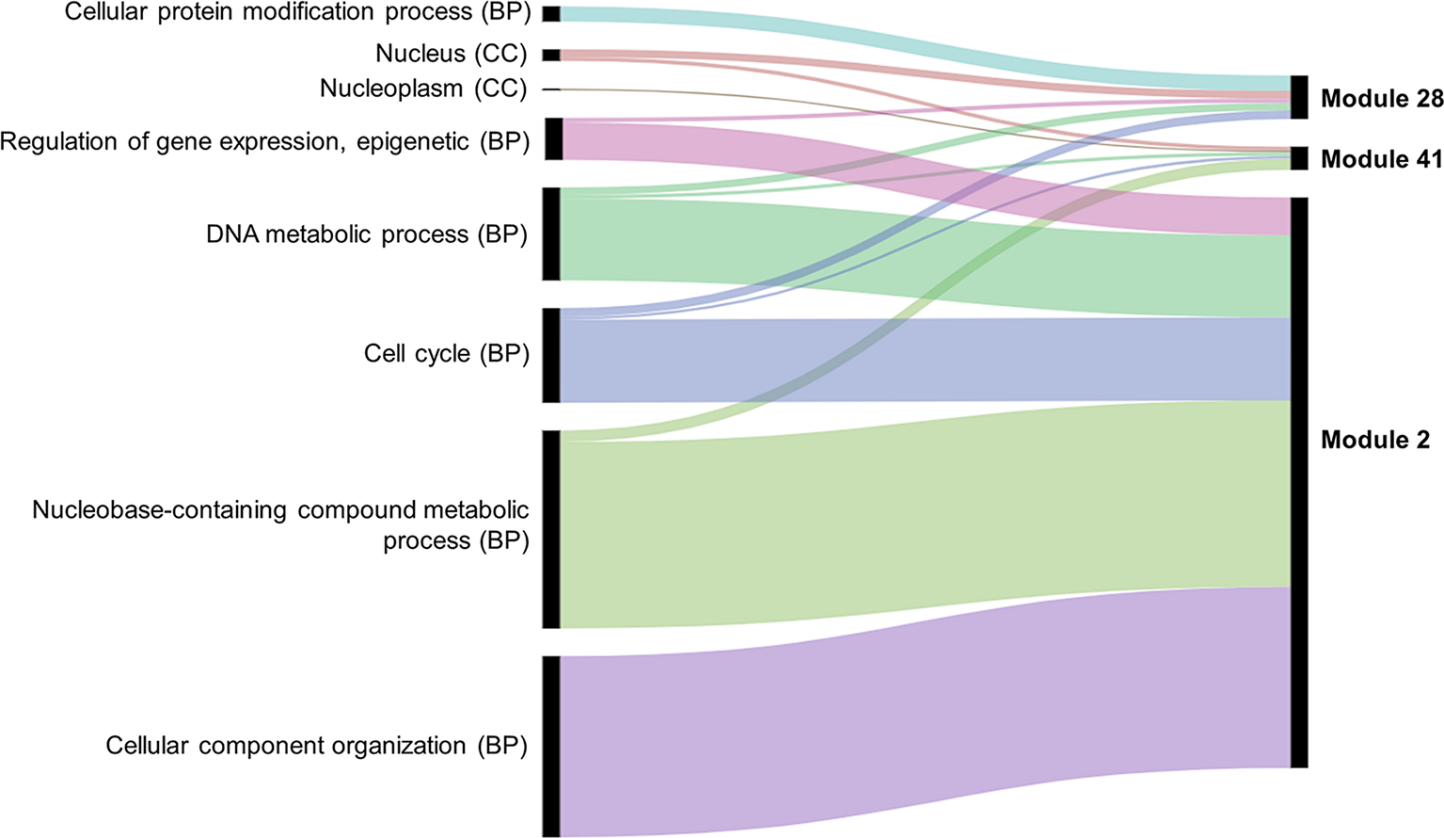
Enriched GO slim terms in the meiosis-related modules. Top five enriched GO terms in each module are shown. BP indicates biological processes and CC cellular component. No molecular function (MF) GO terms appear among the top five GO slim terms. Black bars indicate the number of genes in the GO term.

Several approaches were undertaken to validate the meiosis-related modules. The three modules (2, 28, and 41), strongly correlated with meiotic tissue expression, were found to be significantly enriched with the gene ontology (GO) slim terms “cell cycle,” “DNA metabolic process,” “nucleobase-containing compound metabolic process,” and “nucleus” (**Figure 4**). Among the top five enriched GO slim terms in each of the 66 modules, the term “cell cycle” was significant only in the three meiosis-related modules, suggesting this was not a general property of all modules and was instead specific to the meiosis modules. Module 2 in particular was significantly enriched with GO terms related to many biological processes occurring during meiosis such as “DNA replication,” “histone methylation,” “cytokinesis,” “nucleosome assembly,” and “chromatin silencing” (**Table 1**; **Table S3**). The term “double-strand break repair *via* homologous recombination,” an important process during meiosis, was the primary enriched Biological Processes GO term in module 41 (FDR < 0.05). The biological processes mediated by genes in module 28 included “protein deneddylation,” “positive regulation of G2/M transition of mitotic cell cycle,” “COP9 signalosome,” and other terms related to protein deneddylation and cell cycle control (**Table 1**). GO terms of meiosis-related modules were compared with those of modules highly correlated with other tissues. The GO terms “chloroplast,” “plastids,” “thylakoid,” and “photosystem” were significantly enriched in module 1, the most highly correlated module with leaves. The terms related to protein ubiquitination and protein binding were enriched in module 5 (the most highly correlated module with grain), while the terms “lignin biosynthetic process,” “phenylpropanoid metabolic process,” and “response to wounding” were enriched in module 7, the largest module correlated with roots (**Figure S4**). This indicated that our co-expression module-tissue correlation was meaningful both from the biological and physiological points of view. Detailed information of the enriched GO and GO slim terms in all modules is listed in the supplementary table **Table S3**. In summary, GO analysis confirmed that the three modules (2, 28, and 41) were enriched for genes associated with meiotic processes.

**Table 1:** Top five enriched GO terms in the meiosis-related modules for each ontology group.

	GO term (FDR adjusted *P* value)		
	Biological process	Molecular function	Cellular components
**Module 2**	Cell proliferation (2.2 x 10^−222^)	Protein heterodimerization activity (1.5 x 10^−93^)	Nucleosome (9.3 x 10^−162^)
	DNA replication (4.8 x 10^−151^)	DNA binding (1.8 x 10^−69^)	Nuclear chromatin (2 x 10^−100^)
	Histone H3-K9 methylation (1.6 x 10^−137^)	Microtubule binding (1.3 x 10^−41^)	Chromosome (3.5 x 10^−95^)
	DNA-dependent DNA replication (5.4 x 10^−135^)	DNA-dependent ATPase activity (1.6 x 10^−32^)	Pericentric heterochromatin (3.2 x 10^−88^)
	Regulation of DNA replication (2.4 x 10^−127^)	Motor activity (4.4 x 10^−31^)	Heterochromatin (6.7 x 10^−84^)
**Module 28**	Protein deneddylation (3.6 x 10^−16^)	Apurinic or apyrimidinic site) endonuclease activity (3.3 x 10^−05^)	COP9 signalosome (3.3 x 10^−21^)
	Positive regulation of G2/M transition of mitotic cell cycle (5.4 x 10^−11^)	RNA cap binding (1.8 x 10^−03^)	Nucleus (6.8M x 10^−10^)
	COP9 signalosome assembly (1.2 x 10^−7^)	NADH activity (3.2 x 10^−3^)	Nuclear cap binding complex (1.5 x 10^−6^)
	Mitotic recombination (1.5 x 10^−7^)	Enoyl-[acyl-carrier-protein] reductase activity (3.2 x 10^−3^)	Protein-containing complex (1.9 x 10^−6^)
	Photomorphogenesis (1.7 x 10^−7^)	Signaling receptor activity (3.9 x 10^−3^)	Cortical cytoskeleton (1.5 x 10^−3^)
**Module 41**	Double-strand break repair *via* homologous recombination (3.9 x 10^−5^)	Methyl-CpG binding (1 x 10^−4^)	Nuclear euchromatin (7.3 x 10^−5^)
	Somatic cell DNA recombination (1.1 x 10^−4^)	siRNA binding (1.6 x 10^−3^)	Nucleus (1 x 10^−4^)
	Megasporocyte differentiation (4.3 x 10^−4^)	SUMO transferase activity (2.1 x 10^−3^)	RNA polymerase IV complex (1.1 x 10^−3^)
	Gene silencing by RNA (5.5 x 10^−4^)	DNA binding (5.7 x 10^−3^)	RNA polymerase II, core complex (4 x 10^−3^)
	Positive regulation of sulfur metabolic process (1.1 x 10^−3^)	Cytosine C-5 DNA demethylase activity (1 x 10^−2^)	Proteasome regulatory particle, base subcomplex (4.4 x 10^−3^)

### Enrichment of Meiosis-Related Modules for Wheat Orthologs of Known MGs

An assessment was undertaken to confirm that the meiosis-related modules were enriched for wheat orthologs of known MGs. Although the first wheat meiotic cDNA clones were isolated concurrently with the early discoveries of MGs in other plants (Ji and Langridge, 1994), the identification of MGs in wheat has been hampered by the large wheat genome size, its polyploid nature, and the absence of a complete genome sequence. Thus, in comparison to model plants (*Arabidopsis* and rice), few MGs have been functionally characterized in wheat. Characterized wheat MGs include *TaASY1* (Boden et al., 2007; Boden et al., 2009), *TaMSH7* (Lloyd et al., 2007), *TaRAD51* (Khoo et al., 2008; Devisetty et al., 2010), *TaDMC1* (Khoo et al., 2008; Devisetty et al., 2010), *TaPSH1* (Khoo et al., 2012), *TaZIP4* (Martín et al., 2017; Martín et al., 2018; Rey et al., 2018), and *RecQ-7* (Gardiner et al., 2019). Given that, the assessment of whether the three modules contain known MGs was undertaken using orthology informed approaches. A set of 1,063 candidate MGs in wheat was identified and categorized based on the method used to identify the genes:

- The “orthologs” group contained 407 genes (**Table S4**; Sheet 1) that correspond to wheat orthologs of 103 functionally characterized MGs in model plant species. The majority of these genes (97.5%) were retrieved from the *Ensembl* Plant ortholog database, while for MGs with no wheat orthologs identified using this method, potential wheat orthologs were identified by searching for amino acid sequence similarity using BLASTP (see Materials and Methods). A list of 10 wheat gene IDs that are potentially orthologs of 4 MGs (*AM1*, *ATM*, *FANCM*, and *ZYP1*) were identified using the BLASTP methods. These four genes were included in our analysis due to their importance for meiosis in other plant models. However, using homology methods (like BLASTP) to infer orthology has a considerably high false positive error rate (Chen et al., 2007). Thus, the corresponding proteins of those 10 genes together with any conclusions drawn from their identification are tentative and must be treated with caution. There were no apparent wheat orthologs for 14 plant MGs (*AtDFO*, *AtNACK2*, *AtPRD2*, *AtRECQ4B*, *AtSGO1*, *CDKG1*, *GIG1*, *JASON*, *MMD1*, *MS5*, *OsMEL1*, *OsMOF*, *PANS1*, and *XRI*).
- The “meiotic GO” group contained 927 wheat genes annotated with one or more meiotic GO terms (**Table S4**; Sheet 2).

**Figure 5:**
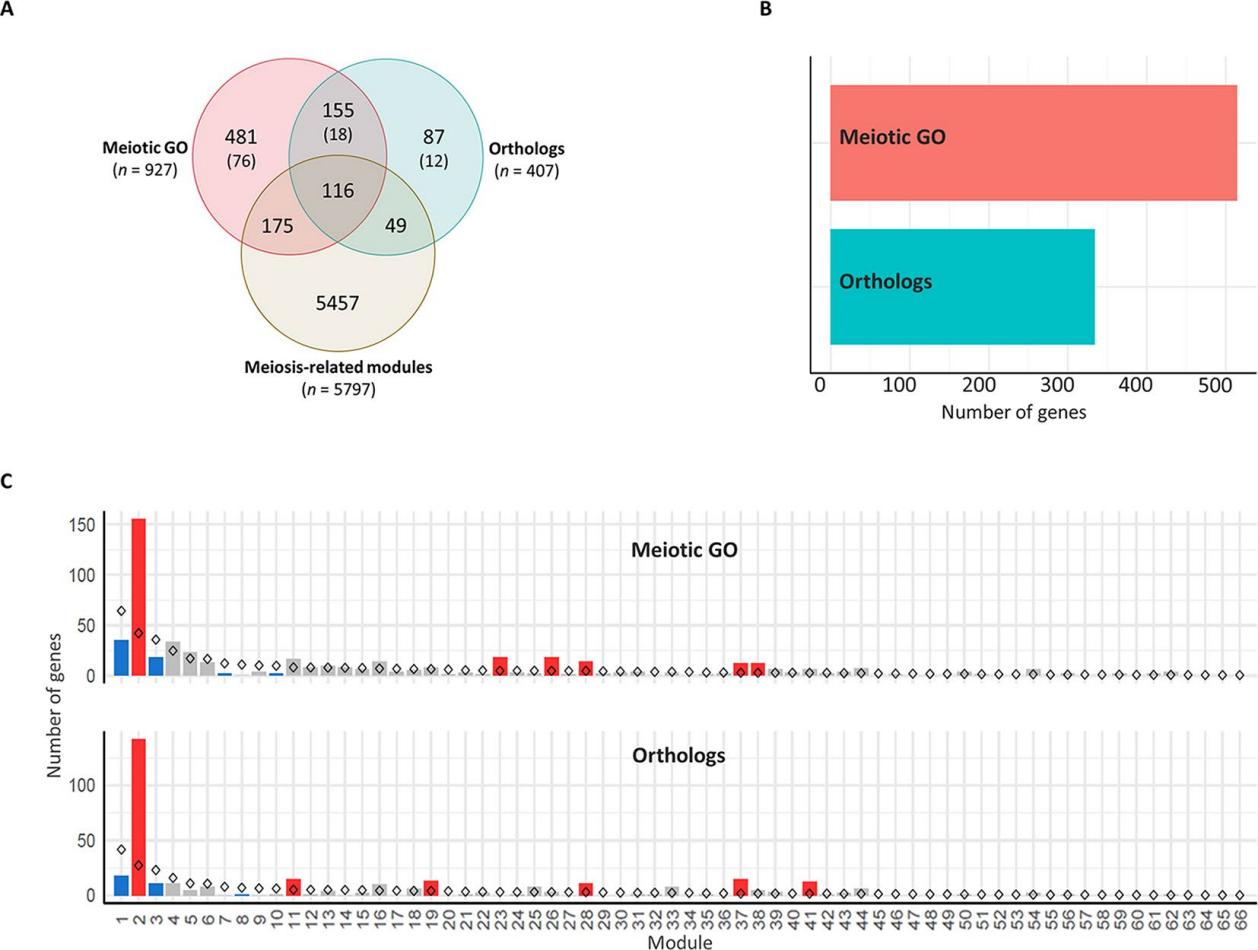
Enrichment of meiosis-related genes in the co-expression network modules. **(A)** Venn diagram of total number of genes in the three groups: meiosis-related modules (genes in modules 2, 28, and 41), orthologs (wheat orthologs of MGs in model plant species), and meiotic GO (genes with meiotic GO terms). *N* indicates number of genes in each group. Numbers in brackets refer to number of genes not included in the WGCNA analysis because they are not expressed in meiotic anther tissue. **(B)** Total number of genes assigned to modules from orthologs of MGs (orthologs) and genes with meiotic GO terms (meiotic GO). **(C)** Gene enrichment in modules. Statistical significance of gene enrichment in a module is color coded (red indicates over-represented, blue under-represented, and gray not significant; *P* < 0.05). Rhombus shape indicates the expected number of genes in module.

There were 271 genes overlapping between the two groups (**Figure 5A)**, which were considered in the “orthologs” group when undertaking gene enrichment analysis. The presence of each gene in the different modules was determined. A set of 848 genes was assigned to modules in the co-expression network (**Figure 5B**), including 340 genes in meiosis-related modules. Genes from both groups were significantly over-represented (*P* < 0.05) in four modules, including two meiosis-related modules (2 and 28). Module 2, in particular, was the most enriched for these genes, possessing more than one third of the total candidate MGs assigned to modules. Module 2 had 142 wheat orthologs of MGs and 155 genes with meiotic GO terms, compared to the expected numbers (based on module size) of 27 and 42, respectively. Module 41 (the third meiosis-related module) was enriched only with genes from the “orthologs” group, having 15 orthologs of known MGs, whereas the expected number was 2 (**Figure 5C**). Consistent with this, genes from the “orthologs” and/or “meiotic GO” groups were significantly under-represented in modules strongly correlated with other types of tissue (modules 1, 7, and 9), and in modules with negative or no correlation with meiotic tissue (modules 3, 8, 14, and 17) (**Table S4**; Sheet 3).

In this study, three co-expression gene modules were identified that are strongly correlated to meiotic anther tissue and highly enriched with GO terms related to many processes occurring during meiosis, orthologs of known MGs, and genes having meiosis-specific GO terms. Although 67 (65%), out of the 103 wheat orthologs, had at least one gene copy assigned to one of the three meiosis-related modules, there were 36 orthologs whose gene homeologs were assigned to other modules (**Table S5**). Some of those genes have essential meiotic functions, like *ASY1*, which encodes a protein essential for homologous chromosome synapsis (Caryl et al., 2000; Armstrong et al., 2002; Boden et al., 2007); and *DMC1*, a gene encoding a recombination protein that acts only in meiosis (Klimyuk and Jones, 1997; Devisetty et al., 2010). Others are known to have both meiotic and mitotic functions, like *BRCA2*, a DNA repair gene required for double strand breaks repair by homologous recombination (Trapp et al., 2011) and *SMC1* and *SMC3*, chromosome cohesion genes (Lam et al., 2005); thus, they are expressed in both reproductive and non-reproductive tissues. Assessment of these 36 orthologs showed that the expression patterns of their gene copies did not allow them to be clustered in any of the meiosis-related modules (or allocated to module 0 that is composed of genes not forming part of a co-expressed module), either because they were expressed in most samples from all types of tissues, or because they were expressed in a few samples of a specific tissue type (like meiotic anther tissue). The expression values (TPMs) of all gene copies of those 36 orthologs are summarized in **Table S5**. The number of meiotic anther samples (17) used in the present study might not be enough to identify all MGs being expressed in a specific meiotic stage. Such genes might be identified by WGCNA analysis when a larger number of meiotic samples become available. However, the analysis confirmed that the meiosis-related modules were indeed enriched for orthologs of known MGs, and for GO terms associated with processes involved in meiosis.

### Copy Number of MGs

**Figure 6:**
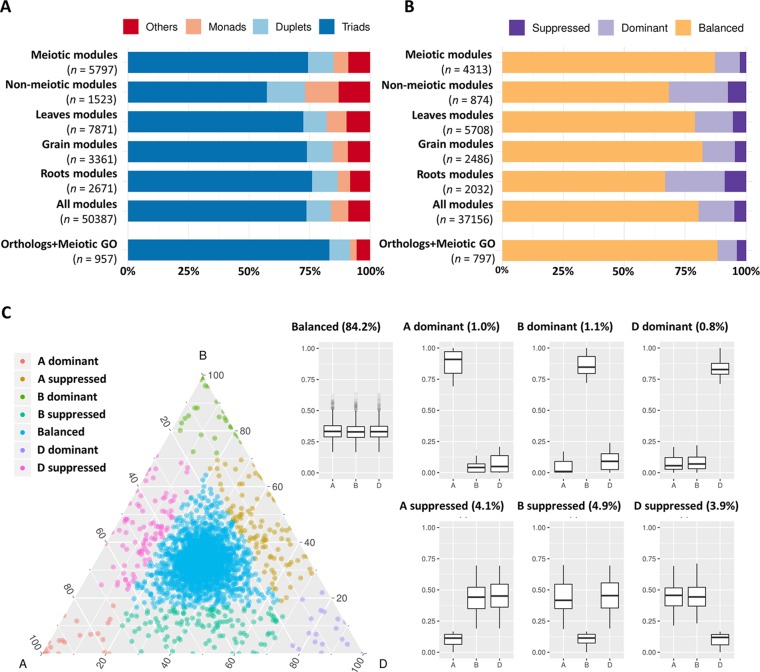
Copy number and homeolog expression pattern for genes from meiosis-related and other tissue-related modules. **(A)** Proportion of genes in each copy number category (triads, duplets, monads, and others) for different sets of expressed genes during meiosis including: “meiotic modules” refers to the three meiosis-related modules 2, 28, and 41. “Non-meiotic modules” refers to the modules 11 and 25 that showed high correlation with meiotic anther but were not considered meiosis-related because they were also correlated with spike and floral organs tissues. The top three correlated modules with each of leaves (modules 1, 45, and 60), grain (modules 5, 13, and 32), and roots (modules 7, 9, and 64) tissues. “All modules” contains all genes assigned to modules in the co-expression network, and “orthologs and meiotic GO” refers to the set of genes that are orthologs of known MGs in other plant species and/or have meiotic GO terms. *n* number of genes in each set. **(B)** Proportion of genes from each homeolog expression pattern category (balanced, dominant, and suppressed) calculated for triads in the previously mentioned sets of genes. *n* number of genes in each set. **(C)** Ternary plot showing relative expression abundance in meiotic anther tissue of 2,366 triads to which the genes of meiosis-related modules (2, 28, and 41) belong. Each circle represents a gene triad with an A, B, and D coordinate consisting of the relative contribution of each homeolog to the overall triad expression. Triads in vertices correspond to single-subgenome-dominant categories, whereas triads close to edges and between vertices correspond to suppressed categories. Box plots indicate the relative contribution of each subgenome based on triad assignment to the seven categories (balanced, A dominant, B dominant, D dominant, A suppressed, B suppressed, D suppressed). Percentages between brackets indicate the percentage of triad number in each category to the total number of triads.

It has previously been suggested that MG duplicates return to a single copy following whole genome duplication more rapidly than the genome-wide average in angiosperms (Lloyd et al., 2014). The analysis of 19 meiotic recombination genes in hexaploid wheat and oilseed rape showed no evidence of gene loss after polyploidization. However, a recent study in tetraploid oilseed rape showed that reducing the copy number of *MSH4*, a key meiotic recombination gene involved in the ZMM (an acronym stands for the MGs Zip1/Zip2/Zip3/Zip4, Msh3/Msh5, and Mer3 identified initially in yeast) pathway, prevents meiotic crossovers between non-homologous chromosomes (Gonzalo et al., 2019). This led to the suggestion that meiotic adaptation in polyploids could involve “fine-tuning” the progression or the effectiveness of meiotic recombination, which could be achieved through the loss of MG duplicates in the newly formed polyploids (Lloyd et al., 2014; Gonzalo et al., 2019). This hypothesis was evaluated in hexaploid wheat. The gene copy number was assessed for the genes in the three meiosis-related modules and compared with genes in all modules and in other tissue-related modules. Analysis showed that the percentage of genes belonging to triads was 74.4% in the meiosis-related modules, which was similar to this percentage in other tissue-related modules (72.5, 74.0, and 76.1% in leaf-, grain-, and root-related modules, respectively); however, it was significantly higher than those of the non-meiotic modules (57.4%). The highest percentage of genes with three homeologs (83.3%) and lowest percentage of genes with single copy (2.7%) were observed in the group of genes identified as MG orthologs and/or possessing a meiotic GO term (**Figure 6A**).

The high percentage of meiosis-related genes present as triads provides evidence that polyploid wheat did not experience significant gene loss (gene erosion) after polyploidization. However, this assumes that these genes were originally present as single copy genes in each of the A-, B-, and D-genome progenitor species which gave rise to polyploid wheat. Therefore, the copy number of the 103 wheat MG orthologs in wheat progenitor species was investigated. All possible orthologs (high and low confidence predicted orthologs) were retrieved from *Ensembl* Plants Genes 43 database for *Triticum urartu* (ASM34745v1; Ling et al., 2013), the diploid progenitor of the wheat A-genome; the D-genome ancestor *Aegilops tauschii* (Aet_v4.0; Luo et al., 2017), the diploid progenitor of the wheat D-genome; and *Triticum dicoccoides* (WEWSeq_v.1.0; Avni et al., 2017), the tetraploid progenitor of the hexaploid wheat (genome AABB). There was no change in copy number of 78.4% of genes, while 6.3 and 15.3% of genes had a lower and greater number of copies, respectively (**Table 2** and **Table S6**). Regardless of genome of origin, the percentage of MGs with more copies was always greater than the percentage of genes with fewer copies. Comparing the A-genome MG copy number in hexaploid wheat with the relevant orthologs copy number in the corresponding A-genome ancestor, 86 genes (84.5%) had the same gene copy number in *T. dicoccoides* as in hexaploid wheat, while only 64 genes (63.1%) had the same gene copy number in *T. urartu*. This is consistent with the evolutionary history of hexaploid wheat, with *T. dicoccoides* being a more recent wheat progenitor (∼10,000 years) than *T. urartu* (> 5 million years) (Marcussen et al., 2014).

**Table 2:** Changes in copy number of wheat MGs in comparison with their orthologs in wheat progenitors.

	Number of meiotic genes (%)			Number of meiotic recombination genes (%)		
	Lower copy number	Greater copy number	Equal copy number	Lower copy number	Greater copy number	Equal copy number
**A genome (*Triticum urartu*)**	9 (8.7%)	29 (28.2%)	64 (63.1%)	3 (4.7%)	21 (32.8%)	40 (62.5%)
**A genome (*Triticum dicoccoides*)**	5 (4.9%)	11 (10.7%)	86 (84.5%)	3 (4.7%)	9 (14.1%)	52 (81.3%)
**B genome (*Triticum dicoccoides*)**	6 (5.8%)	12 (11.7%)	84 (82.5%)	3 (4.7%)	6 (9.4%)	55 (85.9%)
**D genome (*Aegilops tauschii*)**	6 (5.8%)	11 (10.7%)	85 (83.5%)	4 (6.3%)	5 (7.8%)	55 (85.9%)
**Average per all genomes**	7 (6.3%)	16 (15.3%)	81 (78.4%)	3 (5.1%)	10 (16.0%)	51 (78.9%)

Comparison was done for two sets of genes: wheat orthologs of MGs (n = 103) and wheat meiotic recombination genes (n = 64).

An analysis on a subset of wheat genes, which were expected to be involved in meiotic recombination based on the function of their orthologs in model plants (64 genes; **Table S6**), was conducted. Again, results showed that the majority (94.9%) of those genes had greater or no change in number of copies (**Table 2**). Given it has been suggested that the reduction in the copy number of ZMM pathway genes could stabilize meiosis in *Brassica* (Gonzalo et al., 2019), the copy number of the wheat orthologs of seven ZMM genes was evaluated. Five of the seven ZMM genes (*MER3*, *MSH5*, *ZIP4*, *PTD*, and *SHOC1*) had equal or greater number of copies. However, *TaMSH4* gained one A-genome copy (comparing with *T. urartu*) and lost one D-genome copy (compared with *Ae. tauschii*), while *TaHEI10* lost A-genome copy and gained a D-genome copy (**Table S6**). In conclusion, our findings did not support any significant gene loss upon the polyploidization of hexaploid wheat, as suggested for other polyploids (Scannell et al, 2006; Lloyd et al., 2014; Gonzalo et al., 2019).

### Homeolog Expression Patterns in Triads of MGs

Initial analysis revealed that most genes expressed during meiosis showed balanced expression between homeologs (**Figure S1**). The analysis was repeated using gene expression within the validated meiosis modules. Genes from all modules were assigned to three categories (balanced, dominant, and suppressed). Homeolog expression patterns in triads showed that meiosis-related modules 2, 28, and 41 had the highest percentage (87.3%) of genes with balanced expression (belong to balanced triads), compared to the top three tissue-related modules for grain, leaves, and roots (**Figure 6B**). Surprisingly, the group of candidate MGs selected for being orthologs of known MGs in other plant species and/or having meiotic GO terms had a higher percentage of genes from balanced triads (88.3%), whereas the modules not considered meiosis-specific (having high correlation with meiotic anther tissue and with spike and floral organs) contained only 68.3% genes with balanced expression (**Figure 6B**). The majority (84.19%) of triads with genes in meiosis-related modules (2,366 triads) showed balanced expression in meiotic anther tissue (**Figure 6C**).

**Figure 7:**
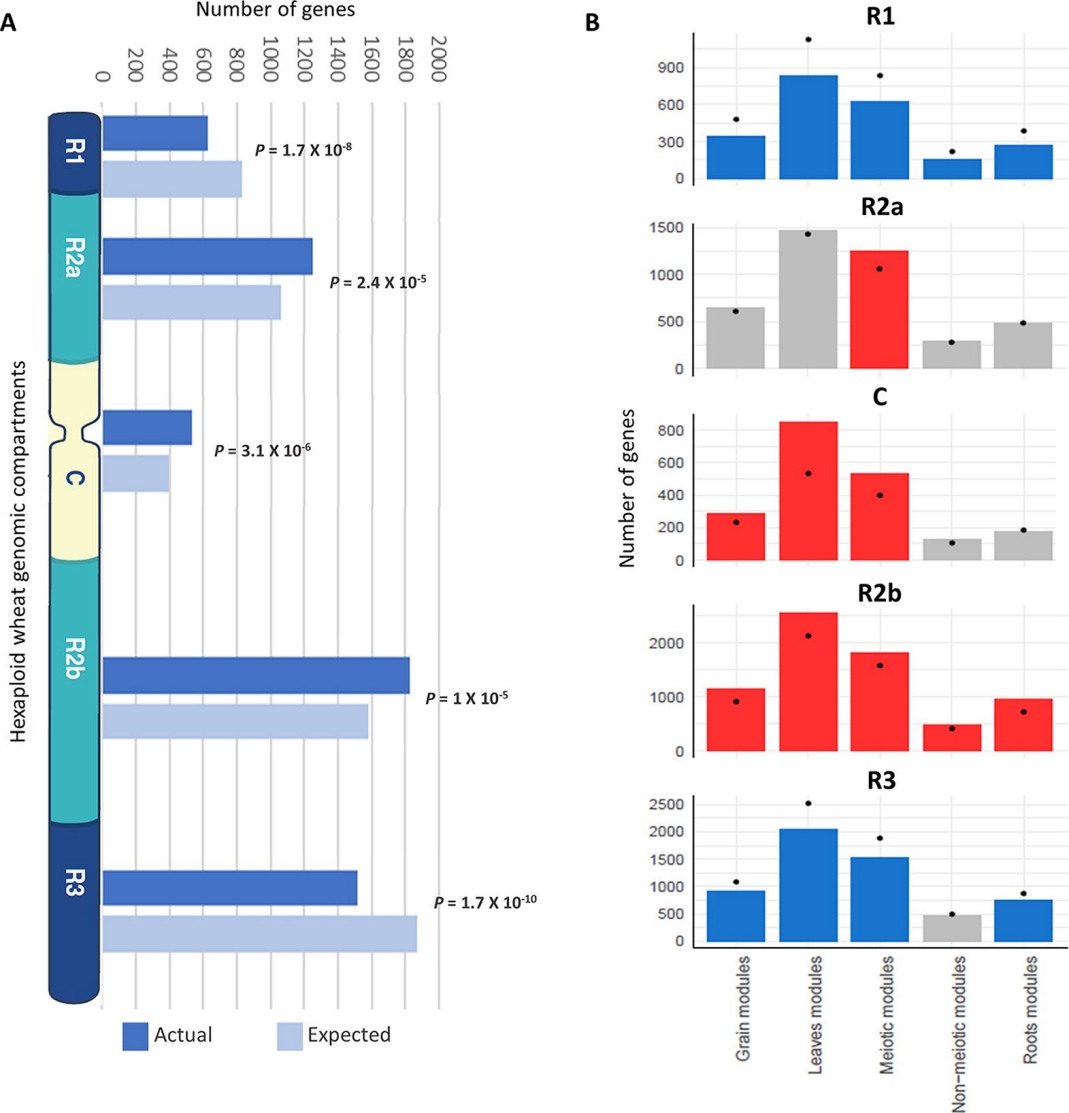
Enrichment of genes from different tissue-related modules in the wheat genomic compartments. **(A)** Number of genes (actual and expected) from the three meiosis-related modules in each genomic compartment. **(B)** Comparison of number of genes from different tissue-related modules in genomic compartments. Statistical significance of gene enrichment in modules is color coded (red indicates enriched, blue depleted, and gray not significant; *P* < 0.05). Black dots indicate the expected number of genes in groups.

In wheat, meiotic recombination and gene evolution rates are strongly affected by chromosome position, with relatively low recombination rates in the interstitial and proximal regions (genomic compartments R2a, C, and R2b) but notably higher rates toward the distal ends of the chromosomes (genomic compartments R1 and R3) (Akhunov et al., 2003; Choulet et al., 2014). The lack of significant changes in gene content and more balanced expression between homeologs suggested that these genes might be more prevalent in the proximal genomic compartments (Ramírez-González et al., 2018; International Wheat Genome Sequencing Consortium, 2018). The distribution of MGs was therefore assessed across the genomic compartments compared with the distribution of all HC genes across chromosomes. Analysis showed that genes from the meiotic modules (modules 2, 28, and 41) were significantly over-represented in the genomic compartments R2a, C, and R2b (*P* = 2.4 x 10^−5^, 3.1 x 10^−6^, and 1 x 10^−5^, respectively), while they were under-represented in the R1 and R3 genomic compartments (*P* = 1.7 x 10^−8^ and 1.7 x 10^−10^, respectively) (**Figure 7A**). Enrichment in the R2a genomic compartment region was not observed for genes from any of the other top three tissue-related modules (**Figure 7B**), since 21.7% of genes from the meiosis-related modules were assigned to R2a, while this percentage ranged between 18.2 and 19.5% in other tissue-related modules (**Table 3**). Interestingly, the set of the genes identified through orthology approaches and MG ontology approaches had also similar high percentage (21%) of genes assigned to R2a genomic compartment (**Table 3**).

**Table 3:** Number of genes from different groups in the wheat genomic compartments.

Modules	R1		R2a		C		R2b		R3	
	No.	%	No.	%	No.	%	No.	%	No.	%
**Meiotic modules**	625	10.9%	1,246	21.7%	531	9.3%	1,823	31.8%	1,515	26.4%
**Non-meiotic modules**	158	10.5%	292	19.5%	123	8.2%	469	31.3%	456	30.4%
**Grain modules**	338	10.2%	649	19.5%	287	8.6%	1,144	34.4%	903	27.2%
**Leaves modules**	838	10.8%	1,466	18.9%	853	11.0%	2,557	33.0%	2,037	26.3%
**Roots modules**	270	10.3%	479	18.2%	177	6.7%	951	36.1%	754	28.7%
**All modules**	5,147	10.3%	9,880	19.9%	4,507	9.1%	16,491	33.1%	13,738	27.6%
**Orthologs and meiotic GO**	78	6.5%	253	21.0%	164	13.6%	387	32.1%	324	26.9%

**Figure 8:**
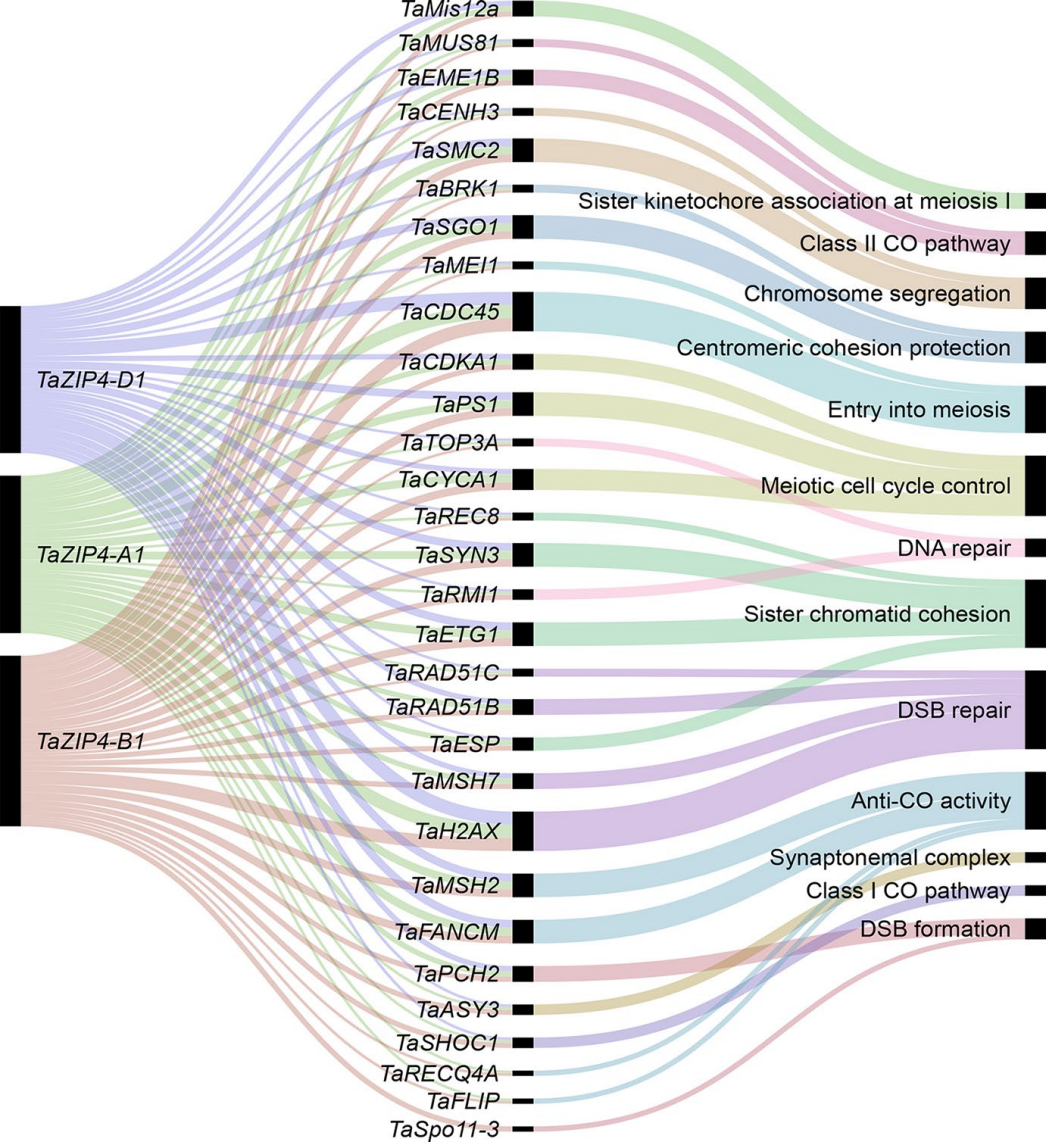
The wheat MG orthologs connected to *TaZIP4*. The alluvial diagram shows the connected genes to the *TaZIP4* homeologs *TaZIP4-A1*, *TaZIP4-B1*, and *TaZIP4-D1*. Edge weight > 1 was used as threshold to visualize connected genes. Black bars indicate the number of homeologs for each connected gene.

Our analysis reveals that homeologous MGs on homeologs mostly show balanced expression and lack a significant change in MG content following polyploidization. The majority of homeologous genes (not only MGs) on homeologs also show over 95% sequence identity to each other (Schreiber et al., 2012; International Wheat Genome Sequencing Consortium, 2018). Given these observations, such homeologs could synapse and recombine during meiosis. However, in allohexaploid wheat, homologs rather than homeolog synapse and recombine during meiosis ensuring the stability and fertility of this species, and the *Ph1* locus, in particular to the *TaZIP4* gene copy inside this locus, has been identified as the main locus controlling this process. The wheat *ZIP4*, an ortholog of *ZIP4/Spo22* in *A. thaliana* and rice, is a member of the ZMM genes involved in the synaptonemal complex formation and class I crossover pathway (Chelysheva et al., 2007; Shen et al., 2012). Moreover, wheat lacking *Ph1* exhibits extensive genome rearrangements, including translocation, duplications, and deletions (Martín et al., 2018). Thus, the evolution of *Ph1* during wheat polyploidization is likely to explain why wheat has largely maintained a similar gene content and balanced expression of its homeologs. How meiosis has adapted to cope with allopolyploidy in other species is still to be resolved; however, it has been suggested that reduction in the copy number of MGs may stabilize the meiotic process after polyploidization (Lloyd et al., 2014; Gonzalo et al., 2019). The present study shows that this is not the case in wheat. It is likely that the presence of *Ph1* in wheat enabled the retention of multiple copies of MGs as an alternative mechanism to ensure proper segregation of chromosomes during meiosis. The identification of the *TaZIP4* gene within the *Ph1* locus as the gene responsible for the *Ph1* effect on recombination and the observed effects of *Ph1* in wheat suggests that it may have more of a central role in meiosis than originally suspected from studies on model systems (Chelysheva et al., 2007; Shen et al., 2012). It has recently been suggested that *ZIP4* might act as a scaffold protein facilitating physical interactions and assembly of different proteins complexes (De Muyt et al., 2018). Therefore, our co-expression network was used to identify the wheat orthologs of known MGs connected with *TaZIP4*. The analysis indicates that the three *TaZIP4* homeologs on group 3 chromosomes (TraesCS3A02G401700, TraesCS3B02G434600, and TraesCS3D02G396500) were clustered in module 2, the largest meiosis-related module, and strongly connected to many orthologs of MGs with various meiotic functions (**Figure 8**). However, the *TaZIP4* copy responsible for *Ph1* phenotype (TraesCS5B02G255100) did not cluster in the same module, reflecting its different expression profile from the other homeologs, being expressed in most tissues (Martín et al., 2017; Martín et al., 2018; Rey et al., 2018). The analysis reveals that *TaZIP4* is connected to several genes involved in centromere function. Studies on budding yeast have suggested that *ZIP4* may affect centromere pairing during meiosis (Kurdzo et al., 2017). Moreover, the *Ph1* locus has been shown to affect centromere pairing during meiosis in hexaploid wheat (Martinez-Perez et al., 2001). Therefore, it will be important to assess whether *TaZIP4* within the *Ph1* locus is responsible for this centromere effect. *TaZIP4* was connected to wheat orthologs of genes known to be involved in crossover formation such as *MSH2*, *SHOC1*, *FANCM*, *FLIP*, *EME1B*, and *MUS81* (Mercier et al., 2015). This suggests that there may be an interplay between *TaZIP4* and genes from the anti-crossover pathway. This may be important as knockouts of genes involved in the anticrossover pathway have been shown to increase crossovers in other crops (Mieulet et al., 2018). However, on wheat’s polyploidization, *TaZIP4* has been duplicated and diverged in order to improve homolog pairing and prevent homeolog crossover (Martín et al., 2018; Rey et al., 2018). This event may also affect the action of these anticrossover genes and the effects of their knockouts. Thus, *TaZIP4* sub-network analysis supports a more central role of *ZIP4* in meiosis than originally suspected from studies on model species.

### Further Characterization of the Wheat Meiotic Co-expression Network

#### Identification of Hub Genes in the Meiosis-Related Modules

**Figure 9:**
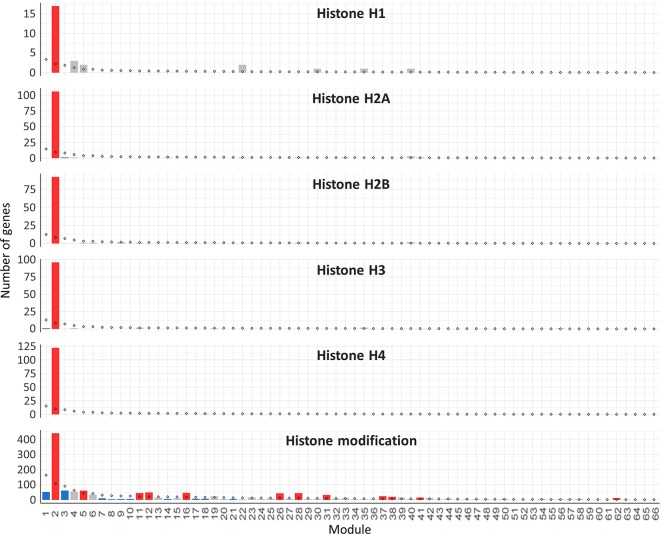
Histone genes enrichment in the gene co-expression network modules. The analysis included all the genes annotated as core histones (H1, H2A, H2B, H3, and H4) in the wheat genome and the genes with GO terms related to histone modification. Statistical significance of gene enrichment in a module at *P* < 0.05 is color coded (red indicates enriched, blue depleted, and gray not significant). Rhombus shape indicates the expected number of genes in module.

Hub genes were identified within our meiosis-related modules by calculating the correlation between expression patterns of each gene and the ME: the most highly correlated genes to the eigengene being the hub genes. The top 10 hub genes of each module with their functional annotation are shown in **Table 4**. The top 10 hub genes in module 2 were core histone genes, supporting the strong contribution of histones in this meiosis-related module. For further verification of histone involvement in module 2 and other modules in general, all wheat genes annotated as core histones or having GO terms related to histone modification were retrieved for enrichment analysis. Analysis showed that the five types of histones (H1, H2A, H2B, H3, and H4) were enriched only in module 2 (*P* = 3.6 x 10^−4^, 1.2 x 10^−22^, 1.1 x 10^−19^, 9.4 x 10^−21^, and 3.3 x 10^−26^, respectively), having 433 genes (85% of all core histone genes in all modules), compared to an expected number of genes of 39 (**Figure 9**). Similar results were obtained for histone modification genes. Module 2 was the most enriched module with this group of genes (*P* = 9.3 x 10^−52^), containing 438 genes (30% of all histone modification genes in all modules). The histone modification genes were also enriched in 11 other modules, including the other meiosis-related modules (modules 28 and 41), however, with much lower numbers of enriched genes (**Figure 9**). Detailed information about genes included in this analysis is provided in **Table S7**. The strong enrichment of histone modification genes in module 2 (the largest meiosis-related module) supports the important role of histone modifications in meiosis (Maleki and Keeney, 2004; Oliver et al., 2013; Hu et al., 2015; Luense et al., 2016; Xu et al., 2016; Wang et al., 2017).

**Table 4:** The top 10 hub genes of each meiosis-related module with their functional annotation.

	Gene	Functional annotation	GO terms
**Module 2**	TraesCS1B02G192500	Histone H2B	Nucleosome; DNA binding; protein heterodimerization activity
	TraesCS1D02G286600	Histone H4	Nucleosome; DNA binding; nucleosome assembly; protein heterodimerization activity
	TraesCS3A02G534100	Histone H2A	Nucleosome; DNA binding; protein heterodimerization activity
	TraesCS6A02G034300	Histone H2A	Nucleosome; DNA binding; protein heterodimerization activity
	TraesCS6A02G034600	Histone H2A	Nucleosome; DNA binding; protein heterodimerization activity
	TraesCS6B02G048300	Histone H2A	Nucleosome; DNA binding; protein heterodimerization activity
	TraesCS6B02G049000	Histone H2A	Nucleosome; DNA binding; protein heterodimerization activity
	TraesCS6D02G326800	Histone H2B	Nucleosome; DNA binding; protein heterodimerization activity
	TraesCS7B02G408400	Histone H2A	Nucleosome; DNA binding; nucleus; protein heterodimerization activity
	TraesCSU02G095700	Histone H3	Nucleosome; DNA binding; protein heterodimerization activity
**Module 28**	TraesCS1A02G263400	Zinc finger CCCH domain protein	DNA binding; protein binding; zinc ion binding
	TraesCS2B02G281900	Receptor kinase	Protein kinase activity; protein binding; ATP binding; protein phosphorylation
	TraesCS2D02G000100	Histone deacetylase complex subunit SAP30	Protein binding
	TraesCS2D02G263500	Receptor kinase	Protein kinase activity; protein binding; ATP binding; protein phosphorylation
	TraesCS5B02G379400	Vacuolar protein sorting-associated 2-2-like protein	Vacuolar transport
	TraesCS5D02G070800	Aminotransferase	Catalytic activity; biosynthetic process; pyridoxal phosphate binding
	TraesCS5D02G386000	Vacuolar protein sorting-associated 2-2-like protein	Vacuolar transport
	TraesCS6A02G253900	High mobility group protein	Chromatin assembly or disassembly; chromatin binding; chromatin remodeling; DNA binding
	TraesCS6B02G271600	High mobility group protein	Chromatin assembly or disassembly; chromatin binding; chromatin remodeling; DNA binding
	TraesCSU02G072600	Vacuolar protein sorting-associated 2-2-like protein	Vacuolar transport
**Module 41**	TraesCS1D02G003800	Serine/threonine-protein kinase ATM	NA
	TraesCS1D02G072800	Chaperone protein dnaJ	Cytoplasm; protein folding; unfolded protein binding; heat shock protein binding; response to stress
	TraesCS2A02G120400	Adenine nucleotide transporter 1	NA
	TraesCS2D02G401100	High mobility group family	Nucleus; DNA binding; transcription factor activity; sequence-specific DNA binding
	TraesCS3A02G162200	Zinc finger CCCH domain-containing protein 4	Metal ion binding
	TraesCS3D02G127500	Ankyrin repeat protein-like	Protein binding
	TraesCS4D02G284200	F-box family protein	Protein binding
	TraesCS5A02G170400	F-box protein	Protein binding
	TraesCS7A02G233900	Poor homologous synapsis 1 protein	Nucleus; synapsis; intracellular signal transduction; kinase activity
	TraesCS7D02G181800	Interleukin-6	NA

Hub genes such as “*poor homologous synapsis 1*” (*PHS1*) were also identified with module 41, the module most highly correlated to meiotic samples. This gene has been previously reported to have a key role in homologous chromosome pairing, synapsis, DNA recombination, and accurate chromosome segregation during meiosis in maize (Pawlowski et al., 2004), *Arabidopsis* (Ronceret et al., 2009), and wheat (Khoo et al., 2012). Other hub genes identified in modules 28 and 41 encoded for the high mobility group proteins (Tessari et al., 2003; Di Agostino et al., 2004; Pedersen et al., 2011; Antosch et al., 2015; Alonso-Martin et al., 2016), histone deacetylase (Akiyama et al., 2004; Wang et al., 2006; Magnaghi-Jaulin and Jaulin, 2006; Getun et al., 2017), and F-box proteins (Zheng et al., 2016).

#### Analysis of Transcription Factors Within the Meiosis-Related Modules

**Figure 10:**
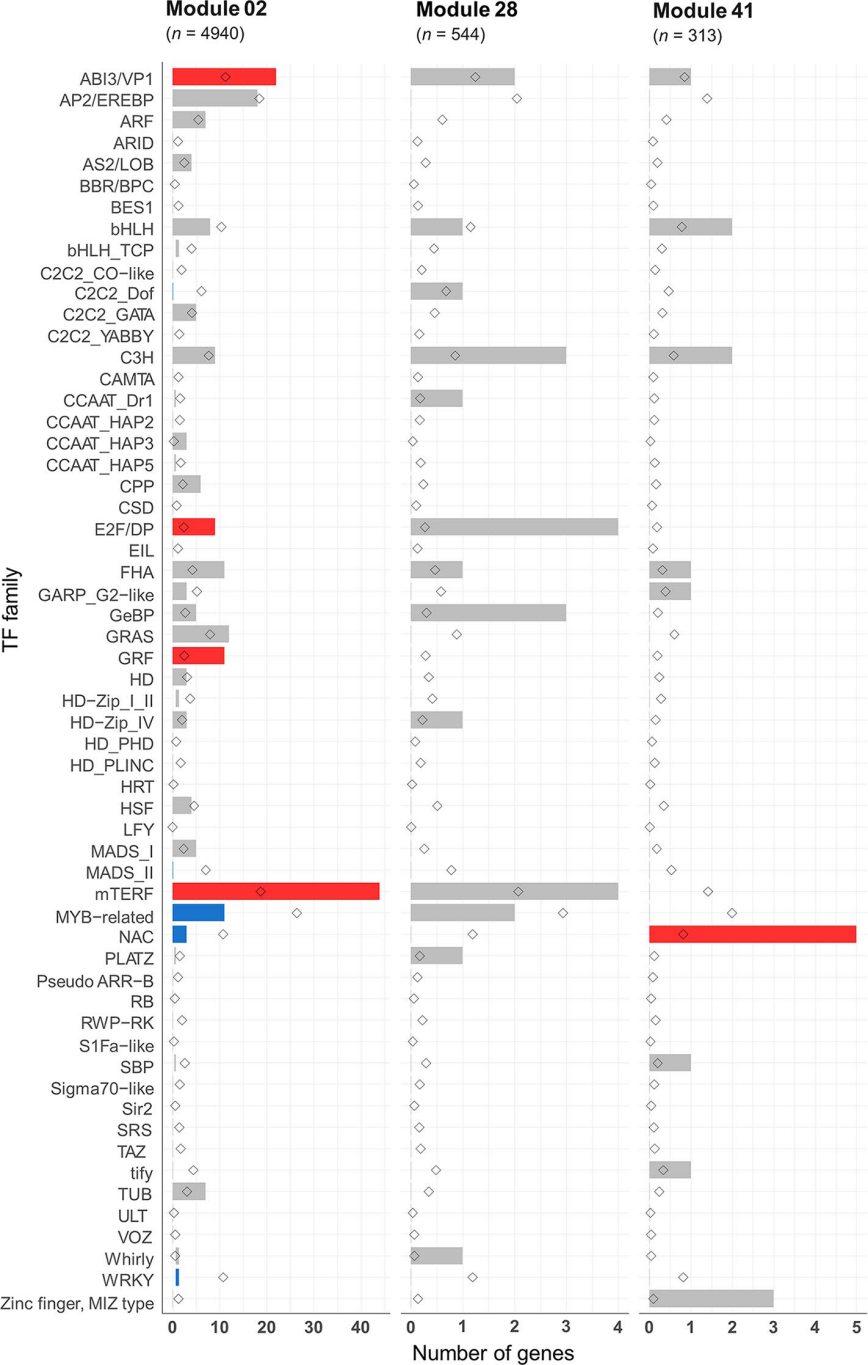
Transcription factor families in the meiosis-related modules. Statistical significance of gene enrichment in the modules is color coded (red indicates over-represented, blue under-represented, and gray not significant; *P* < 0.05). Rhombus shape indicates the expected number of genes in module.

Many transcription factors (TFs) have been reported as key regulators of meiosis from studies on animals (Bolcun-Filas et al., 2011; Yan et al., 2016), yeast (Xu et al., 1995; Horie et al., 1998; Pierce et al., 2003; Beaudoin et al., 2018), and protozoa (Zhang et al., 2018). However, very little is known about the involvement of TFs in plant meiosis. The meiotic co-expression network was therefore exploited to identify potential meiosis-specific TFs. An assessment was undertaken of the enrichment of previously identified TF families in hexaploid wheat in the meiosis-related modules 2, 28, and 41. A total of 4,889 HC genes belonging to 58 TF families were predicted from the annotation of the wheat genome sequence. Of these, 2,439 TFs from 57 families could be assigned to the 66 modules in the gene co-expression network. Modules 2, 28, and 41 (meiosis-related modules) had 225, 25, and 17 TFs belonging to 31, 13, and 9 TF families, respectively (**Table S8**). Compared to the expected number of TF family genes in each module, only five TF families were significantly enriched in module 2: mitochondrial transcription termination factor (mTERF), growth-regulating factor (GRF), abscisic acid-insensitive protein 3/viviparous1 (ABI3/VP1), forkhead-associated domain (FHA), and E2F/dimerization partner (DP). On the other hand, four TF families were significantly depleted (**Figure 10**). The TF family NAC was the only TF family significantly enriched in module 41, containing 5 NAC genes (expected number 0.6; *P* < 0.05). Module 28 was not enriched with any TF family, although E2F/DP TFs were enriched in this module with borderline statistical significance (*P* = 0.06), with 4 genes in this module (the expected number was 0.2). Except in module 2, E2F/DP and FHA TF families were not enriched in any other modules in the gene co-expression network (**Table S8**). E2F/DP plays an important role in regulating gene expression necessary for passage through the cell cycle in mammals and plants (Zwicker et al., 1996; Zheng et al., 1999; Sozzani et al., 2006). Members of FHA contain the forkhead-associated domain, a phosphopeptide recognition domain found in many regulatory proteins. Genes belonging to the FHA group are reported to have roles in cell cycle regulation (Hollenhorst et al., 2000; Zhu et al., 2000; Kim et al., 2002), DNA repair (Sun et al., 1998; Bashkirov et al., 2003; Palmbos et al., 2008; Liang et al., 2015), and meiotic recombination and chromosome segregation (Pérez-Hidalgo et al., 2003; Cigliano et al., 2011; Crown et al., 2013). A previous meiotic transcriptome study identified up-regulation of TFs belonging to the MADS-box, bHLH, bZIP, and NAC families in *Arabidopsis* and maize meiocytes at early meiosis (Dukowic-Schulze et al., 2014). Zinc finger-like TFs have also been suggested to be regulators of maize MG expression (Ma et al., 2008). The present study indicates that TF families known to have roles in cell cycle, and meiosis processes are over-represented in the meiosis-related modules (module 2 in particularly). Those TF families contain about 20 meiosis-specific candidate TF genes whose function can be validated using the available reverse genetics resources in polyploid wheat (Krasileva et al., 2017).

#### Visualization of Networks and Identification of Candidate MGs

**Figure 11:**
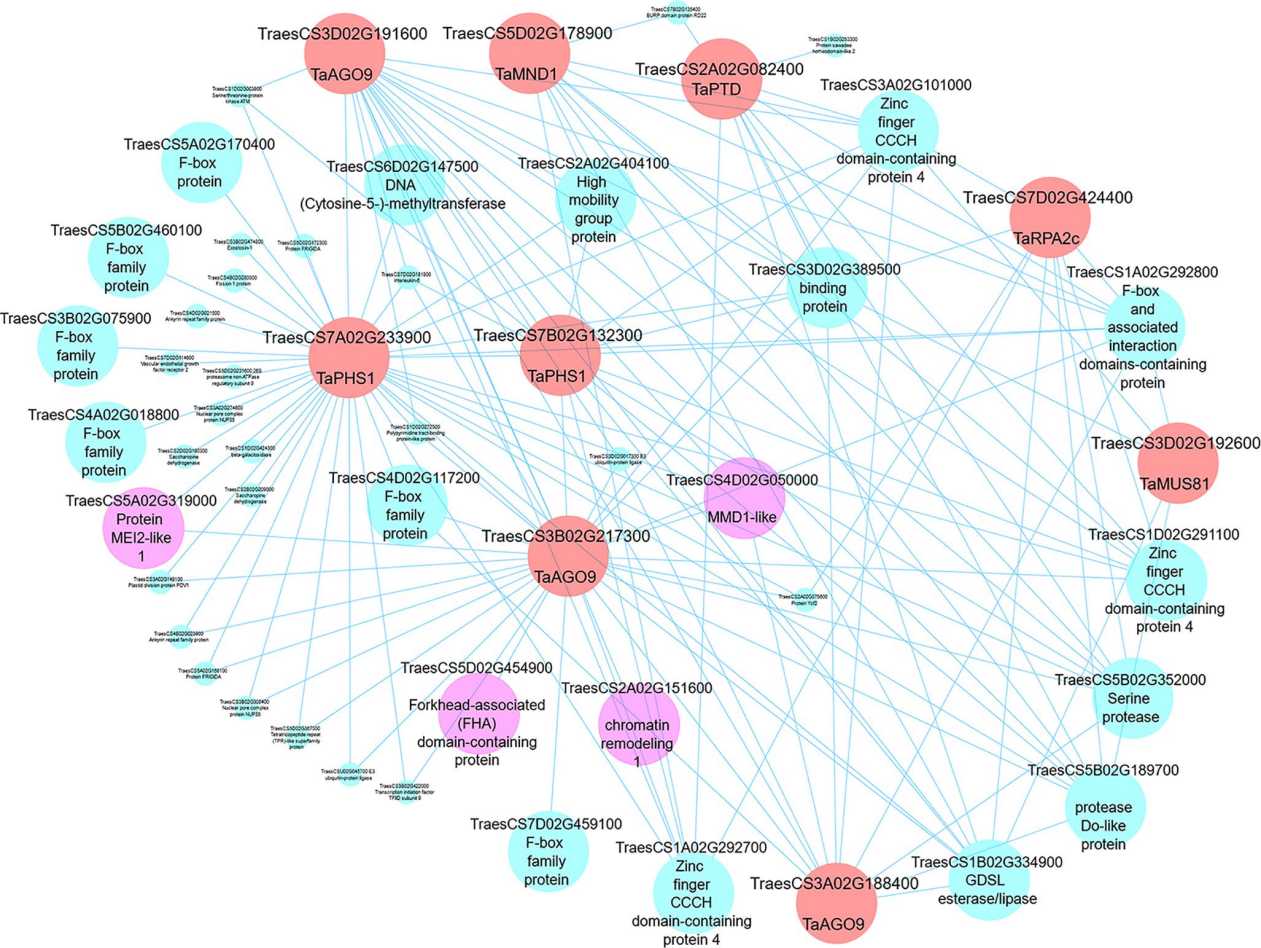
A meiotic co-expression subnetwork in hexaploid wheat. This subnetwork was constructed using 9 guide genes in module 41. Guide genes are wheat orthologs of MGs in other plant species (red circles); pink circles represent genes with putative meiosis function. Edge weight 0.05 was used as threshold to visualize genes in the subnetwork using Cytoscape 3.7.1 software.

Having identified meiosis-related modules, the networks within such modules can be visualized, highlighting genes for future studies. Edge files were created with gene annotation for the three meiosis-related modules 2, 28, and 41. Those files can be used to investigate the relation between orthologs of MGs within a module and ranked based on the strength of the connection (weight value). Another application of co-expression networks is the identification of previously uncharacterized genes regulating biological processes (Usadel et al., 2009; Aya et al., 2011; Costa et al., 2015; Silva et al., 2016; Krishnan et al., 2017; Tan et al., 2017; International Wheat Genome Sequency Consortium, 2018; Ma et al., 2018; Yu et al., 2018; Liu et al., 2019). Cytoscape 3.7.1 software (Shannon et al., 2003) was therefore used to visualize our network and to show connections between different orthologs of MGs in meiosis-related modules. Wheat MG orthologs in meiosis-related modules were used as “guide genes” to construct co-expression subnetworks containing only genes with direct connections to the guide genes. One such subnetwork is shown in **Figure 11**, where the following wheat orthologs of MGs in module 41 were selected and used to construct a meiotic subnetwork: *poor homologous synapsis 1* (*TaPHS1*; Khoo et al., 2012), *argonaute* (AGO9/AGO104; Durán-Figueroa and Vielle-Calzada, 2010; Singh et al., 2011), *replication protein A2c* (*OsRPA2c*; Li et al., 2013), *meiotic nuclear division protein 1* (*AtMND1*; Domenichini et al., 2006; Kerzendorfer et al., 2006), *MMS and UV sensitive 81* (*AtMUS81*; Hartung et al., 2006; Higgins et al., 2008), and *parting dancers* (*AtPTD*; Wijeratne et al., 2006; Lu et al., 2014) (guide genes; red circles in **Figure 11**). The network complexity was reduced using an edge weight > 0.05. The visualized subnetwork contained 53 gene IDs including 9 guide gene copies. The gene TraesCS7A02G233900 (*TaPHS1*), a hub gene in module 41, was central in the network having the highest number of direct edges (41 direct edges; connected with 77.4% of the genes in the subnetwork). This subnetwork allowed identification of other genes with putative roles in meiosis (pink circles): (a) RNA recognition motif-containing gene (TraesCS5A02G319000) similar to Mei2, a master regulator of meiosis and required for premeiotic DNA synthesis as well as entry into meiosis in *Schizosaccharomyces pombe* (Watanabe and Yamamoto, 1994; Watanabe et al., 1997); (b) the gene TraesCS4D02G050000 showed similarity to Male meiocyte death 1 (*MMD/DUET*), a PHD-finger protein plays role in chromatin structure and male meiotic progression in *A. thaliana* (Reddy et al., 2003); and (c) the gene TraesCS5D02G454900, a possible TF belonging to the FHA family known to have function in cell cycle regulation (Hollenhorst et al., 2000; Zhu et al., 2000; Kim et al., 2002), DNA repair (Sun et al., 1998; Bashkirov et al., 2003; Palmbos et al., 2008; Liang et al., 2015), and meiotic recombination and chromosome segregation (Pérez-Hidalgo et al., 2003; Cigliano et al., 2011; Crown et al., 2013). The meiotic subnetwork contained genes with similarity to cell cycle like F-box family proteins, high mobility family proteins, and chromatin remodeling genes. The subnetwork also contained a group of genes connected to most of our guide genes, which thus might be involved with them in similar biological processes. Examples of such genes are TraesCS3A02G101000, TraesCS1A02G292700, and TraesCS1D02G291100 which encode for zinc finger CCCH domain-containing proteins (**Figure 11**). Other meiotic subnetworks were also constructed using other guide genes from modules 2 and 28.

#### The Meiotic Co-Expression Network Is Accessible in a Larger Biological Contest

Our WGCNA co-expression network and GO enrichment data have been integrated with the wheat knowledge network (Hassani-Pak et al., 2016) to make it publicly accessible and searchable through the KnetMiner web application (http://knetminer.rothamsted.ac.uk; Hassani-Pak, 2017). KnetMiner can be searched with keywords (incl. module ID and GO terms) and wheat gene identifiers. The gene knowledge graphs generated contain many additional relation types such protein–protein interactions, homology, and links to genome wide association studies and associated literature placing the co-expression networks generated here in a wider context.

## Conclusion

In summary, the present study shows that most MGs in wheat are retained as three homeologous genes, which are expressed during meiosis at similar levels, suggesting that they have not undergone extensive gene loss nor sub/neo-functionalization. Meiosis-related modules have been used to create networks and identify hub genes providing targets for future studies. The network containing the *ZIP4* gene, recently defined as *Ph1* (Martín et al., 2017; Martín et al., 2018; Rey et al., 2018)—for example, highlights potential interacting partners. Finally, the networks highlight genes such as *ZIP4* and “*poor homologous synapsis 1*,” which may play a more central role in meiosis than previously thought. The co-expression network analysis combined with orthologue information will contribute to the discovery of new MGs and greatly empowers reverse genetics approaches to validate the function of candidate genes (Krasileva et al., 2017). Ultimately, this will lead to better understanding of the regulation of meiosis in wheat (and other polyploid plants) and subsequently improve wheat fertility.

## Materials and Methods

### RNA-Seq Data Collection

For co-expression network analysis, we included 130 samples, containing 113 samples previously described in Ramírez-González et al. (2018) and 17 samples from anthers during meiosis (9 samples from Martín et al. (2018), and 8 samples downloaded from https://urgi.versailles.inra.fr/files/RNASeqWheat/Meiosis/). Samples were selected to represent all main tissue types: grain (*n* = 37 samples), leaves (*n* = 21 samples), roots (*n* = 20 samples), anther at meiosis (*n* = 17 samples), spike (*n* = 12 samples), floral organs (anther, pistil, and microspores) at stages other than meiosis (*n =* 10 samples), stem (*n* = 7 samples), and shoots (*n* = 6 samples). All samples were under nonstress conditions and mostly from the reference accession Chinese Spring. Detailed information about the used samples are listed in the **Supplementary Materials (Table S2)**.

### Mapping of RNA-Seq Reads to Reference

Kallisto v0.42.3 (Bray et al., 2016) was used to map all RNA-Seq samples to the Chinese Spring transcriptome reference IWGSC RefSeq Annotation v1.1 (International Wheat Genome Sequencing Consortium, 2018), following default parameters previously shown to result in accurate homeolog-specific read mapping in polyploid wheat (Borrill et al., 2016; Ramírez-González et al., 2018). Tximport v1.2.0 was then used to summarize expression levels from transcript to gene level (**Text S1**; Part 1).

### Co-Expression Network Construction

The WGCNA package in R (Langfelder and Horvath, 2008; Langfelder and Horvath, 2012) was used to construct the scale-free co-expression network. Metadata for all samples were assigned with eight tissue types (average 16.25; median 14.5 replicates per factor). Only HC genes (International Wheat Genome Sequencing Consortium, 2018) with expression > 0.5 TPM in at least one meiosis sample were retained for co-expression network construction using the R Package WGCNA (version 1.66). Using the varianceStabilizingTransformation() function from DESeq2 (Love et al., 2014), the count expression level of selected genes was normalized to eliminate differences in sequencing depth between different RNA-Seq studies (**Text S1**; Part 2). To select a soft power threshold (β) for adjacency calculation (as a*_ij_* = |s*_ij_*|^β^; where s*_ij_* is the correlation between gene *i* and gene *j*), the scale-free topology criterion was used (Zhang and Horvath, 2005). The soft thresholding means suppressing low correlations in a continuous (“soft”) manner by using β value to power the correlation of the genes to that threshold, which reduces the noise of the correlations in the adjacency matrix. Using the pickSoftThreshold() function to calculate β values, the soft power threshold emphasizing strong correlations between genes and penalizing weak correlations was selected as the first power to exceed a scale-free topology fit index of 0.9 (Ramírez-González et al., 2018) (**Text S1**; Part 3). The correlation type used to calculate adjacency matrices was biweight midcorrelation (bicor). The adjacency matrices were transformed into a topological overlap matrix (TOM), measuring the network connectivity of a gene deﬁned as the sum of its adjacency with all other genes for network generation. The blockwiseModules() function was used to calculate matrices and construct blockwise networks considering the following parameters: network type (NetworkType) = “signed hybrid,” maximum block size (maxBlockSize) = 46,000 genes, soft power threshold (power) = 7, correlation type (corType) = “bicor” (biweight midcorrelation with maxPOutliers set to 0.05 to eliminate effects of outlier samples), topological overlap matrices type (TOMType) = “unsigned” with the mergeCutHeight = 0.15, and the minModuleSize = 30 to classify genes with similar expression proﬁles into gene modules using average linkage hierarchical clustering, according to the TOM-based dissimilarity measure with a minimum module size of 30 genes (**Text S1**; Part 4). MEs, summarizing the expression patterns of all genes within a given module into a single characteristic expression profile, were calculated as the first principal component in the principal component analysis (PCA) using the moduleEigengenes() function (**Text S1**; Part 4).

### Identifying Meiosis-Related Modules

The MEs were used to test correlations between gene modules and traits (eight tissue types) using the cor() function. To assess the significance of correlations, Student asymptotic *P* values for correlations were calculated using the function corPvalueStudent() and corrected for multiple testing by calculating FDR (false discovery rate) using a p.adjust() function following the Benjamini and Yekutieli (2001) method. We considered a module meiosis-related when its correlation was strong with meiosis samples (*r* > 0.5 and FDR < 0.05) and weak (*r* < 0.3) or negative with other type of tissues (**Text S1**; Part 5).

### Analysis of GO Term Enrichment in Modules

GO term enrichment was calculated using the “goseq” package (Young et al., 2010). Gene ontology (GO) annotations of IWGSC RefSeq v1.0 genes were retrieved from the file “FunctionalAnnotation.rds” in https://opendata.earlham.ac.uk/wheat/under_license/toronto/Ramirez-Gonzalez_etal_2018-06025-Transcriptome-Landscape/data/TablesForExploration/FunctionalAnnotation.rds (Ramírez-González et al., 2018) by filtering for ontology “IWGSC+Stress.” GO data was then converted to IWGSC RefSeq Annotation v1.1 by replacing “01G” by “02G” in the IWGSC v1.0 gene IDs and retaining only genes > 99% similar with > 90% coverage in the v1.0 and v1.1 annotation versions (as determined by BLASTn of the cDNAs) (called “all_go”). *P* values for GO term enrichment were calculated using the goseq() function (using the following parameters: the pwf object was created using the nullp() function which calculated a probability weighting function for the genes v1.1 based on their length, the gene2cat = all_go, and test.cats = “GO : BP,” to specify the biological process GO term category to test for over representation amongst the inquired genes) and corrected using the FDR method (Benjamini and Hochberg, 1995). A GO term was considered enriched in a module when FDR adjusted *P* value < 0.05 (**Text S1**; Part 6). All figures shown for enriched GO terms in the modules were produced using RAWGraphs software (Mauri et al., 2017).

### Orthologs of MGs in Wheat

A comprehensive literature search was performed for MGs in model plant species (mainly *A. thaliana* and rice; **Table S9**), identifying gene IDs based on the “Os-Nipponbare-Reference-IRGSP-1.0” for rice (*Oryza sativa* Japonica Group) and “TAIR10” for *A. thaliana*. Wheat orthologs of MGs were then retrieved from *Ensembl* Plants Genes 43 database through BioMart (in; http://plants.ensembl.org/biomart) where orthologs calculated according to Vilella et al. (2009) using the following gene datasets: “*Triticum aestivum* genes (IWGSC),” “*Oryza sativa* Japonica Group genes (IRGSP-1.0),” and “*A. thaliana* genes (TAIR10)” for wheat (International Wheat Genome Sequencing Consortium, 2018), rice (Kawahara et al., 2013; Sakai et al., 2013), and *A. thaliana* (Waese et al., 2017), respectively. For MGs with no wheat orthologs identified using this method, potential wheat orthologs were identified by searching for amino acid sequence similarity using BLASTP (Korf et al., 2003) in *Ensembl* Plants according to the following criteria: e-value < 1e-10; ID% > 25% with *Arabidopsis* and > 70% with rice. By blasting the amino acid sequences of rice and *Arabidopsis* MGs against wheat proteins, a list of genes (that do not have rice and *Arabidopsis* MG orthologs in the Ensembl Plants database) was identified. For this list of wheat genes, we checked whether they have any other rice or *Arabidopsis* orthologs. Then, only wheat genes that did not have any rice or *Arabidopsis* orthologs were considered as orthologs of MGs. Finally, 407 wheat gene IDs were identified as orthologs of 103 plant MGs (listed in **Table S4**; Sheet 1). This group of genes was referred to in this study as “orthologs.”

### Wheat Genes With MG Ontology (GO)

A total number of 46,909 GO terms used by Ramírez-González et al. (2018) to calculate GO term accessions for wheat genes (IWGSC v1.0 gene annotation) were filtered for meiosis-related GO terms, using 15 meiosis-specific keywords (“meiosis,” “meiotic,” “synapsis,” “synaptonemal,” “prophase I,” “metaphase I,” “anaphase I,” “telophase I,” “leptotene,” “zygotene,” “pachytene,” “diplotene,” “chiasma,” “crossover,” and “homologous chromosome segregation”). A total of 284 meiosis GO accessions were identified and used to retrieve 927 wheat genes with potential roles during meiosis (**Table S4**; Sheet 2). All genes identified by gene orthologs and gene ontology methods were then filtered to retain only genes had expression > 0.5 TPM in at least one meiosis sample. This group of genes was referred to in this paper as “meiotic GO.” Enrichment analysis for the genes from “orthologs” and “meiotic GO” groups in all module was conducted (**Text S1**; Part 7). The number of genes from each group was assessed in all modules and compared with the expected number based on the module size. There was a set of genes overlapping between “orthologs” and “meiotic GO” groups, which was considered in the “orthologs” group when undertaking gene enrichment analysis. Fisher’s exact test was used to calculate significant enrichment in the modules. Gene group considered over- or under-represented in a module when *P* < 0.05.

### Identifying Highly Connected Hub Genes

Hub genes within each module were identified using the WGCNA R package function signedKME() to calculate the correlation between expression patterns of each gene and the ME. Hub genes were considered those more highly correlated to the eigengene (**Text S1**; Part 8).

### Assessment of TF Families in Modules

A total of 4,889 wheat HC genes (IWGSC RefSeq Annotation v1.1; International Wheat Genome Sequencing Consortium, 2018) belonging to 58 TF families were predicted from the annotation of the wheat genome sequence (https://github.com/Borrill-Lab/WheatFlagLeafSenescence/blob/master/data/TFs_v1.1.csv). The number of TFs from each family was assessed in all modules and compared with the expected number based on the module size. Fisher’s exact test was used to calculate significant enrichment of TFs in the modules. TF family considered over- or under-represented in a module when *P* < 0.05 (**Text S1**; Part 9).

### Defining Gene Categories Based on Number of Homeologs

A list of homeologs for all HC hexaploid wheat genes (IWGSC v1.1 gene annotation; International Wheat Genome Sequencing Consortium, 2018) was retrieved from *Ensembl* Plants Genes 43 database through BioMart (in; http://plants.ensembl.org/biomart). Based on number of homeologs from each of the A-, B-, and D-sub-genomes, genes were assigned to four groups: triads that refer to 1:1:1 triads (with a single copy from each of the A-, B-, and D-sub-genomes); duplets referring to 1:1:0, 1:0:1, and 0:1:1 duplets; monads group containing genes with no homeologs (e.g. 0:0:1); and “others” containing genes with more than two homeologs, in conjunction with genes from the homeologous groups 0:1:2, 0:2:1, 1:0:2, 2:0:1, 1:2:0, 2:1:0, 2:0:0, 0:2:0, and 0:0:2. Accordingly, 19,801 triads (59,403 genes), 7,565 duplets (15,130 genes), 15,109 monads (single-copy genes), and 18,250 genes from the “others” group were identified (**Table S1**).

### Defining Gene Categories Based on Homeolog Expression Patterns in Triads

Homeolog expression pattern in triads was determined for each of the eight tissue types (**Text S1**; Part 10). For triads, it was calculated according to Ramírez-González et al. (2018) where a triad can be described as balanced, A dominant, A suppressed, B dominant, B suppressed, D dominant, or D suppressed, based on the relative expression contribution of its A, B, and D homeologs. Briefly, the relative contribution of each gene in a triad was calculated with the following formula:

Relative expression contribution (A) = TPM(A)/[TPM(A) + TPM(B) + TPM(D)]

where A, B, and D represent the gene corresponding to the A, B, and D homeologs in the triad. Each category is defined by the following ideal relative expression contributions:

**Table:** 

Category	A	B	D
Balanced	0.33	0.33	0.33
A suppressed	0	0.5	0.5
B suppressed	0.5	0	0.5
D suppressed	0.5	0.5	0
A dominant	1	0	0
B dominant	0	1	0
D dominant	0	0	1

The triad was assigned to the category with the shortest Euclidean distance to its relative contribution. Triads were defined as expressed when one of its homeologs was expressed according to the criterion used in our WGCNA analysis (**Table S10**; Sheet 1). This insured that all triads contain genes from modules were included in the homeolog expression bias analysis (**Text S1**; Part 11). Genes from a triad might not belong to the same module due to dissimilarity of their expression patterns. Thus, to allow the assessment of the expression pattern of genes in each module, each homeolog (A, B, and D homeologs) in a triad was assigned to one of the three categories “balanced,” “dominant,” and “suppressed” based on the homeolog origin (A, B, and D sub-genome) and the triad description (balanced, A dominant, A suppressed, B dominant, B suppressed, D dominant, or D suppressed) as shown in **Table S10** (Sheet 2). The values of the relative contributions of each homeolog per triad were used to plot the ternary diagrams using the R package ggtern (Hamilton, 2016).

### Co-Expression Gene Network Visualization

Cytoscape software (version 3.7.1; Shannon et al., 2003) was used to visualize the network described in this study. Firstly, the “exportNetworkToCytoscape” function was used to create edge files which could be used to visualize the network, then depending on network complexity, different weight value thresholds were used to filter genes to be visualized (**Text S1**; Part 12). The term “weight value” in the input files for Cytoscape refers to the connection strength between two nodes (genes) in terms of correlation value obtained from the topological overlap matrices (TOM). The co-expression network data has also been integrated with the wheat knowledge network (Hassani-Pak et al., 2016) to make it publicly accessible through the KnetMiner web application (http://knetminer.rothamsted.ac.uk; (Hassani-Pak, 2017). The data was semantically modeled as nodes of type gene, co-expression-module, co-expression-study, and GOterm, connected by relations of type part-of and enriched. Each module was given a unique identifier composed of the module number and the prefix “AKA.” KnetMiner can be searched with keywords (incl. module ID and GO terms) and wheat gene identifiers.

## Data Availability Statement

All datasets used in this study can be found in in the Earlham Institute repository (https://opendata.earlham.ac.uk/wheat/under_license/toronto/Martin_etal_2018_Alabdullah_etal_2019_wheat_meiosis_transcriptome_and_co-expression_network/). All R scripts are provided as.text files in the **Supplementary Material**. The gene network data is accessible and searchable through the public KnetMiner website (http://knetminer.com).

## Author Contributions

AA, PB, AM, CU, and GM contributed conception and design of the study. AA, AM, RR-G, and KH-P organized and curated the data; AA performed the formal analysis; AA, PB, and RR-G wrote the R scripts; GM and PS secured the funding and supervised the work. AA wrote the original draft. All authors contributed to manuscript revision, read and approved the submitted version.

## Funding

This work was supported by the UK biotechnology and Biological Sciences Research Council (BBSRC) through a grant as part of Designing Future Wheat (DFW) Institute Strategic Programme (BB/P016855/1) and response mode grant (BB/R007233/1).

## Conflict of Interest

The authors declare that the research was conducted in the absence of any commercial or financial relationships that could be construed as a potential conflict of interest.

## Supplementary Material

Figure S1 Homeolog expression patterns of expressed triads in hexaploid wheat. Homeolog expression pattern was calculated for 19,801 triads (59,403 genes) across 8 tissue types according to published criteria (Ramírez-González et al., 2018), where triad defined as expressed when the sum of the A, B, and D subgenome homeologs was > 0.5 TPM. (A) Proportion of triads in each homeolog expression pattern across the 8 tissues. n is number of expressed triads. (B) Ternary plot showing relative expression abundance of 14,837 expressed triads (44,511 genes) in the meiotic anther tissue. Each circle represents a gene triad with an A, B, and D coordinate consisting of the relative contribution of each homeolog to the overall triad expression. Triads in vertices correspond to single-subgenome–dominant categories, whereas triads close to edges and between vertices correspond to suppressed categories. Box plots indicate the relative contribution of each subgenome based on triad assignment to the seven categories. Percentages between brackets indicate the percentage of triad number in each category to the total number of triads.

Figure S2 Proportion of genes in each homeologs number category. Expressed genes across 8 tissues were assigned to four categories (triads, duplets, monads and others). *n* indicates number of expressed genes.

Figure S3 Module-tissue relationship. Each row corresponds to a module; each column corresponds to a tissue type; Each cell contains the correlation value (*r*) and, in brackets, its corresponding FDR adjusted *P* value. *n* indicates number of samples. Only modules that have correlation value > 0.5 are shown.

Figure S4 Enriched GO terms in the meiosis-related and other tissue-related modules. Top 5 GO terms are shown for each module. Black bars indicate the number of genes in the GO term.
