# Supplementary figures and images for: A Co-Expression Network in Hexaploid Wheat Reveals Mostly Balanced Expression and Lack of Significant Gene Loss of Homeologous Meiotic Genes Upon Polyploidization
Source: Front Plant Sci. 2019 Oct 18;10:1325. doi: 10.3389/fpls.2019.01325 (PMC6813927; doi:10.3389/fpls.2019.01325)

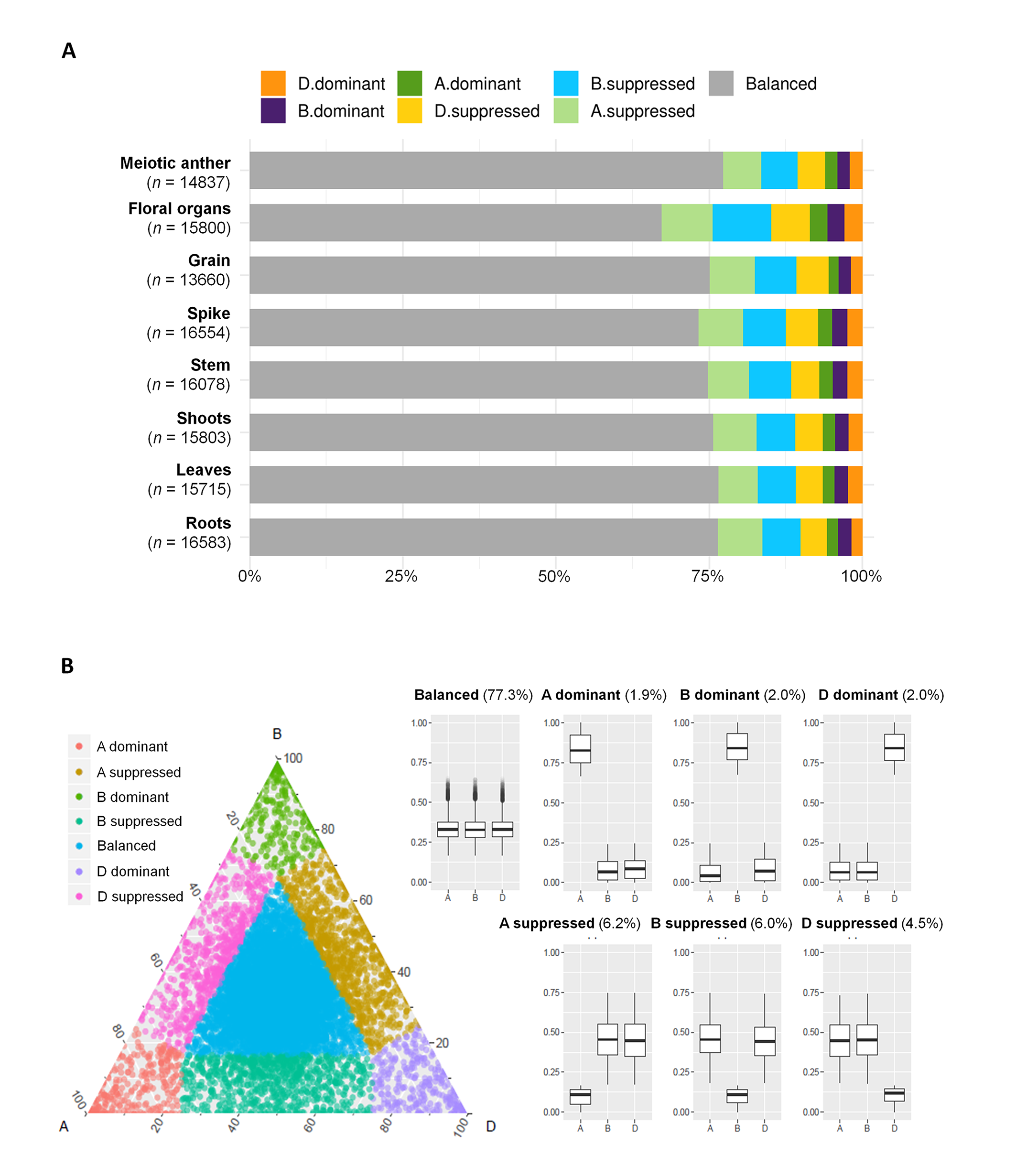

Supplement: Figure S1 — Homeolog expression patterns of expressed triads in hexaploid wheat. Homeolog expression pattern was calculated for 19,801 triads (59,403 genes) across 8 tissue types according to published criteria (Ramírez-González et al., 2018), where triad defined as expressed when the sum of the A, B, and D subgenome homeologs was > 0.5 TPM. (A) Proportion of triads in each homeolog expression pattern across the 8 tissues. n is number of expressed triads. (B) Ternary plot showing relative expression abundance of 14,837 expressed triads (44,511 genes) in the meiotic anther tissue. Each circle represents a gene triad with an A, B, and D coordinate consisting of the relative contribution of each homeolog to the overall triad expression. Triads in vertices correspond to single-subgenome–dominant categories, whereas triads close to edges and between vertices correspond to suppressed categories. Box plots indicate the relative contribution of each subgenome based on triad assignment to the seven categories. Percentages between brackets indicate the percentage of triad number in each category to the total number of triads. [file Image_1.tif]

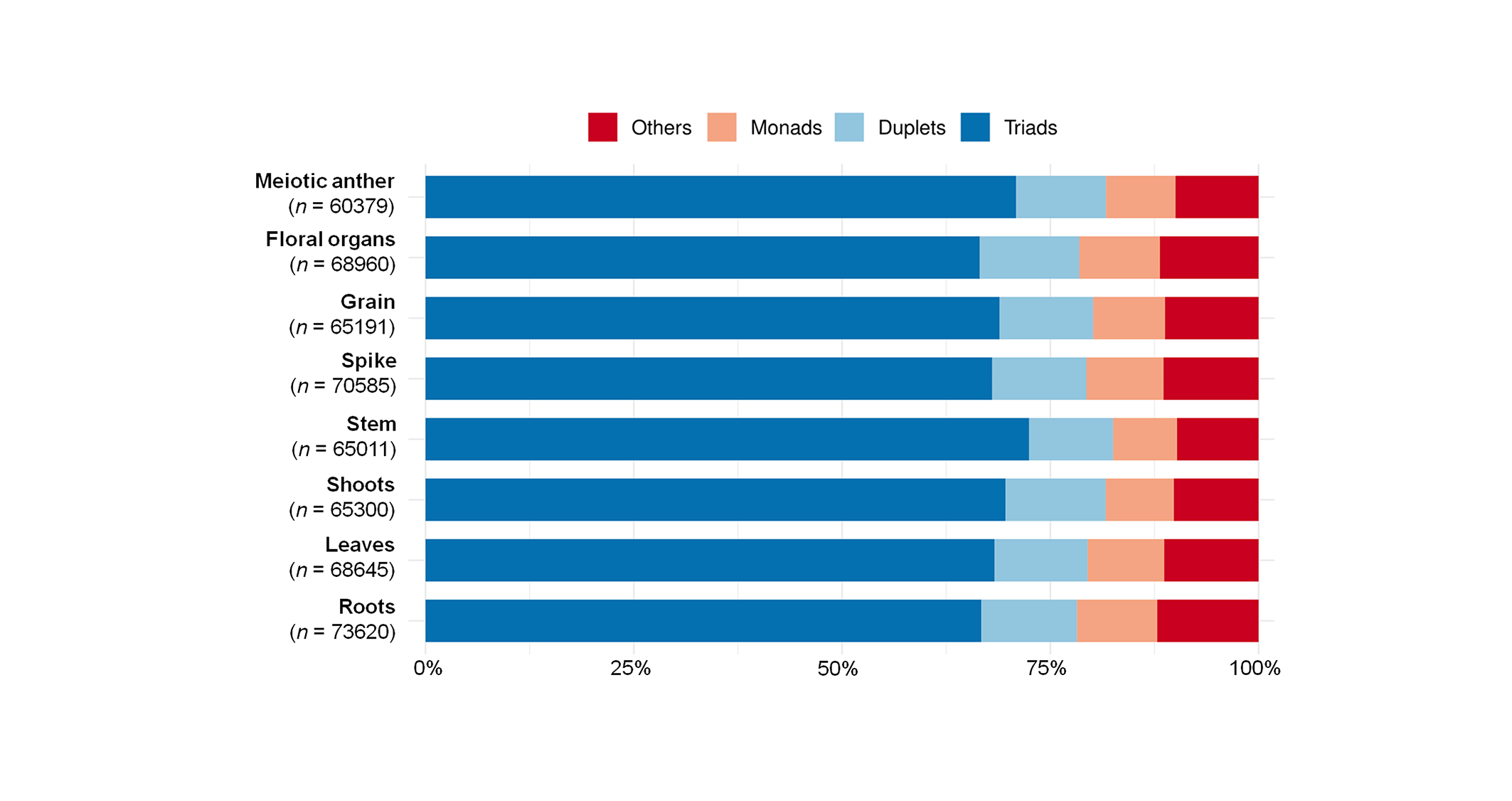

Supplement: Figure S2 — Proportion of genes in each homeologs number category. Expressed genes across 8 tissues were assigned to four categories (triads, duplets, monads and others). n indicates number of expressed genes. [file Image_2.tif]

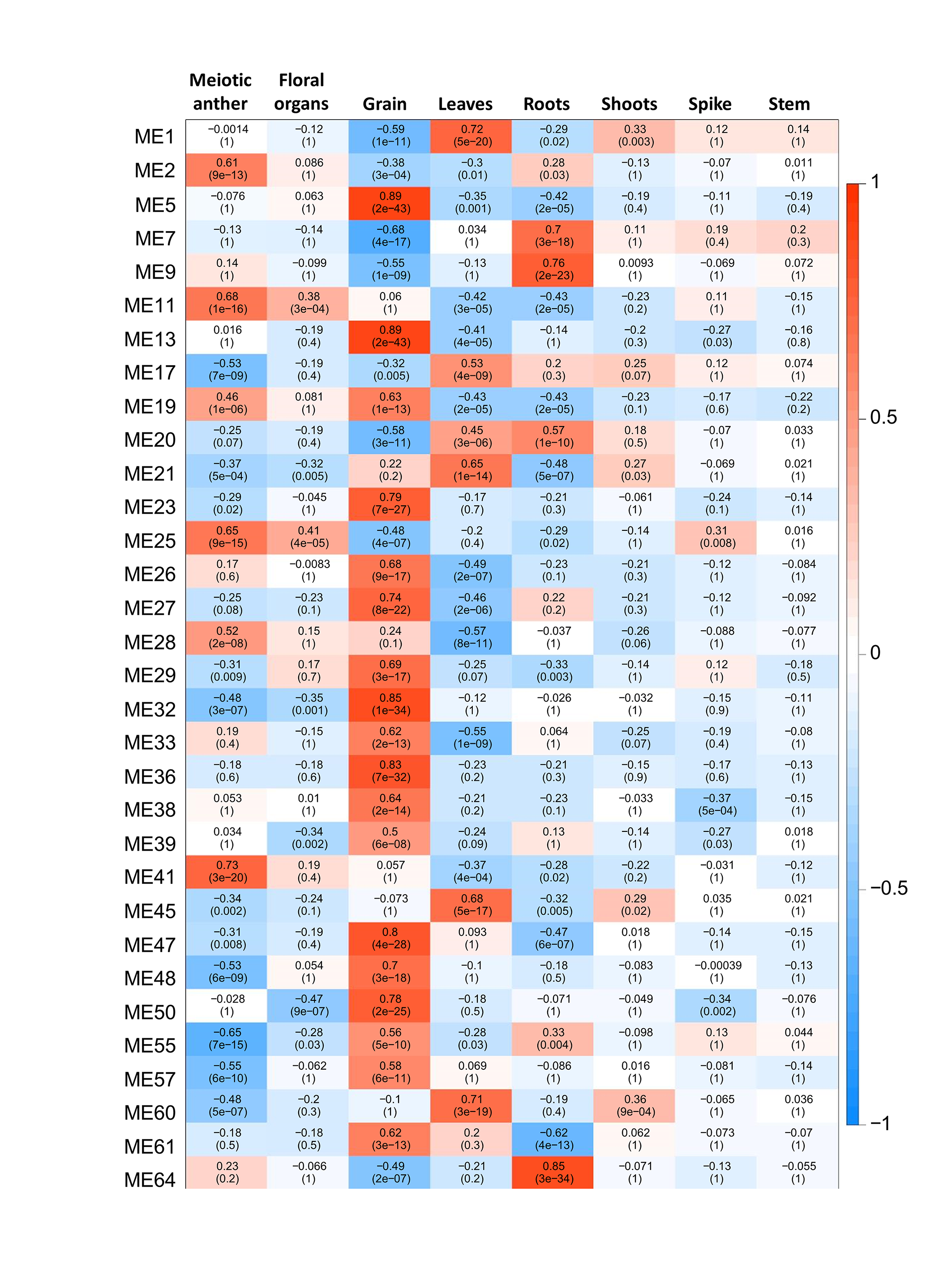

Supplement: Figure S3 — Module-tissue relationship. Each row corresponds to a module; each column corresponds to a tissue type; Each cell contains the correlation value (r) and, in brackets, its corresponding FDR adjusted P value. n indicates number of samples. Only modules that have correlation value > 0.5 are shown. [file Image_3.tif]

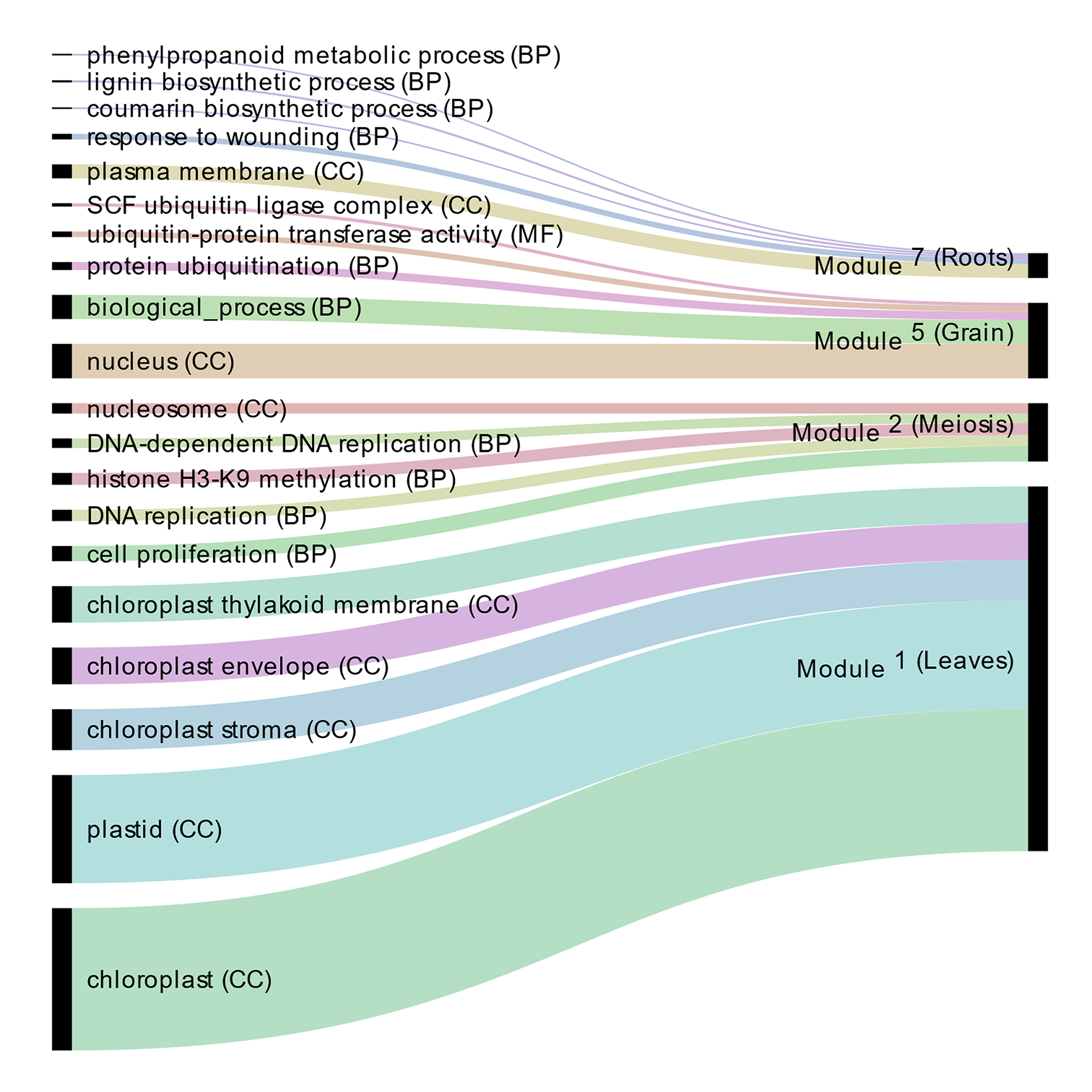

Supplement: Figure S4 — Enriched GO terms in the meiosis-related and other tissue-related modules. Top 5 GO terms are shown for each module. Black bars indicate the number of genes in the GO term. [file Image_4.tif]
