# Supplementary material for: A Co-Expression Network in Hexaploid Wheat Reveals Mostly Balanced Expression and Lack of Significant Gene Loss of Homeologous Meiotic Genes Upon Polyploidization
Source: Front Plant Sci. 2019 Oct 18;10:1325. doi: 10.3389/fpls.2019.01325 (PMC6813927; doi:10.3389/fpls.2019.01325)
Supplement: Supplementary file 15 [file Table_11.docx]

**#Text S1. R scripts used in this study**

**#Part 1: Summarize expression values (counts) from transcript to gene level**

library(tximportData)

library(readr)

library(rhdf5)

setwd("G:\\ABDUL_KADER\\Meiosis_genes_in_wheat\\Gene_Networks\\WGCNA_annoV1.1\\")

study_list <- read.table(file="list_of_studies.txt", header=F)

head(study_list)

colnames(study_list) <- c("study")

head(study_list)

tx2gene <- read.table("transcripts_to_genes_RefSeqv1.0_annot_v1.1.txt", header=T)

head(tx2gene)

for (i in study_list$study) {

setwd("G:\\ABDUL_KADER\\Meiosis_genes_in_wheat\\Gene_Networks\\WGCNA_annoV1.1\\")

study <- i

print(i)

samples <- read.table(paste0(study,".txt"), header=F)

samples

colnames(samples) <- c("sample")

files <- file.path("G:\\ABDUL_KADER\\Meiosis_genes_in_wheat\\Gene_Networks\\WGCNA_annoV1.1\\IWGSC_v1.1_ALL_20170706_transcripts.fasta.k31\\", study, samples$sample, "abundance.tsv", fsep ="\\")

files

names(files) <- paste0(samples$sample)

head(files)

all(file.exists(files))

library(tximport)

# read in the files and sum per gene

txi <- tximport(files, type = "kallisto", txIn = TRUE, txOut = FALSE, countsFromAbundance = c("no"), tx2gene = tx2gene)

names(txi)

setwd("G:\\ABDUL_KADER\\Meiosis_genes_in_wheat\\Gene_Networks\\WGCNA_annoV1.1\\expressionValuesPerGene")

head(txi$counts)

colnames(txi$counts)

# save counts summarized per gene

write.table(txi$counts, file=paste0(study,"_count.tsv"),sep = "\t")

# save tpm summarized per gene

write.table(txi$abundance, file=paste0(study,"_tpm.tsv"),sep = "\t")

# calculate average gene length across all samples

head(txi$length)

gene_lengths <- as.data.frame(rowMeans(txi$length))

head(gene_lengths)

colnames(gene_lengths) <- c("length")

head(gene_lengths)

#save length per gene

write.csv(gene_lengths, file=paste0(study,"_gene_lengths.csv"))

}

**#Part 2: Combine samples from all studies and normalize counts for WGCNA analysis**

library("matrixStats")

library("DESeq2")

library("WGCNA")

library("dendextend")

setwd("G:\\ABDUL_KADER\\Meiosis_genes_in_wheat\\Gene_Networks\\WGCNA_annoV1.1\\")

loadValuesFromExperiment<-function(metadata, folder, unit="tpm", values=c("RNAseq_A_C_Martin")){

v<-values[1]

v<-gsub(" ","_",v)

path<-paste0(folder,"\\",v,"_",unit,".tsv")

ret<-read.table(path, row.names = 1, header= TRUE)

for(i in 2:length(values)){

v<-values[i]

v<-gsub(" ","_",v)

path<-paste0(folder,"\\",v,"_",unit,".tsv")

tmp<-read.table(path, row.names = 1, header= TRUE)

ret<-cbind(ret,tmp)

}

md<-metadata[metadata$Sample.IDs%in%colnames(ret),]

ret<-ret[,as.character(md$Sample.IDs),]

list(ret,md)

}

folder<-"expressionValuesPerGene"

metadata_file<- "metadata.txt"

metadata <- read.csv(metadata_file, row.names = 1, sep="\t")

metadata[1:10,1:4]

nrow(metadata)

tpms <-loadValuesFromExperiment(metadata, folder, unit="tpm", values=unique(metadata$study.title))

counts<-loadValuesFromExperiment(metadata, folder, unit="count",values=unique(metadata$study.title))

metadata_used<-tpms[[2]]

head(metadata_used)

tpms<-tpms[[1]]

counts<-counts[[1]]

nrow(metadata_used)

head(metadata_used)

head(tpms)

colnames(tpms)

colnames(counts)

head(rownames(tpms))

head(rownames(counts))

print("dimension of counts")

print(dim(counts))

#Filter count data to only keep genes expressed >0.5 tpm in at least 1 % of samples (or in 1 sample at least)

tpm_threshold <- 0.5

count_filt <- counts[rowCounts(as.matrix(tpms>tpm_threshold))>=1,]

nrow(counts)

print("dimensions of count_filt for tpm >0.5 in 1 sample")

print(dim(count_filt))

nrow(count_filt)

#Filter to only keep HC genes expressed in meiosis (tpm >0.5 in 1 sample at least of the 17 meiosis samples)

inmeiosis <- read.csv("genes_exp_in_meiosis.csv") # this file has a list of expressed genes in meiosis (60379 genes)

head(inmeiosis)

dim(inmeiosis)

count_filt <- count_filt[rownames(count_filt)%in%inmeiosis$Gene,]

print("dimensions of count_filt for tpm >0.5 in at least 1 meiosis sample and high conf")

print(dim(count_filt))

setwd("G:\\ABDUL_KADER\\Meiosis_genes_in_wheat\\Gene_Networks\\WGCNA_annoV1.1\\HC_exp_in_meiosis\\")

write.csv(count_filt, file="Filtered_counts_HC_exp_in_meiosis_130samples.csv")

# round counts to integers (required for DESeq)

count_filt <- round(count_filt)

dim(count_filt)

#normalize counts

counts_matrix <- as.matrix(count_filt)

counts_matrix[1:6,1:3]

eigengenes <- svd(counts_matrix)$v

write.csv(eigengenes, file="eigengenes_meiosis_only.csv")

vsd_blind <- varianceStabilizingTransformation(counts_matrix,blind=TRUE)

vc <- (vsd_blind)

head(vc)

vsd2 <- t(vc)

vsd2[1:4,1:4]

dim(vsd2) # [1] 130 60379

options(stringsAsFactors = FALSE)

disableWGCNAThreads()

datExpr0 <- vsd2

gsg = goodSamplesGenes(datExpr0, verbose = 3)

print("are all genes ok?")

print(gsg$allOK)

#If the last statement returns TRUE, all genes have passed the cuts. If not, we remove the offending genes and samples from the data with the following:

#if (!gsg$allOK) {

# if (sum(!gsg$goodGenes)>0)

# printFlush(paste("Removing genes:", paste(names(datExpr0)[!gsg$goodGenes], collapse= ", ")));

# if (sum(!gsg$goodSamples)>0) printFlush(paste("Removing samples:", paste(rownames(datExpr0)[!gsg$goodSamples], collapse=", ")))

# datExpr0= datExpr0[gsg$goodSamples, gsg$goodGenes]

#}

#gsg = goodSamplesGenes(datExpr0, verbose = 3)

#print("are all genes ok?")

#print(gsg$allOK)

print("dimensions of datExpr after removing bad genes")

print(dim(datExpr0)) # [1] 130 60379

sampleTree = hclust(dist(datExpr0), method = "average");

pdf(file = paste0("Sample_Clustering_HC_exp_in_meiosis_",tpm_threshold,"tpm.pdf"), width = 20, height = 4)

par(cex = 0.6);

par(mar = c(0,4,2,0))

plot(sampleTree, main = "Sample clustering to detect outliers (only HC genes expressed in meiosis)", sub="", xlab="", cex.lab = 1.5,

cex.axis = 1.5, cex.main = 2)

dev.off()

# save the data to use in the next step

save(datExpr0, metadata_used, file=paste0("filtered_data_HC_only_ready_for_WGCNA_",tpm_threshold,"tpm.RData"))

# now plot dendrogram with colours according to tissue

metadata_selected <- metadata_used

dend <- as.dendrogram(sampleTree)

metadata_selected$High.level.tissue

colorCodes <- c(Meiocytes="purple", Leaves_Shoots="green", Roots="orange", Spike="pale green", Grain="brown")

labels_colors(dend) <- colorCodes[(metadata_selected$High.level.tissue[(order.dendrogram(dend))])]

labels_colors(dend)

head(metadata_selected$High.level.tissue)

head(metadata_selected$High.level.tissue[(order.dendrogram(dend))])

labels_colors(dend)

pdf(file = paste0("Sample_Clustering_HC_exp_in_meiosis_coloured_by_tissue_",tpm_threshold,"tpm.pdf"), width = 40, height = 4)

par(cex = 0.6);

par(mar = c(6,4,2,0))

plot(dend, main = "Sample clustering to detect outliers (only HC genes expressed in meiosis)", sub="", xlab="", cex.lab = 1.5,

cex.axis = 1.5, cex.main = 2)

dev.off()

**#Part 3: Calculate the soft power threshold for the WGCNA analysis**

setwd("G:\\ABDUL_KADER\\Meiosis_genes_in_wheat\\Gene_Networks\\WGCNA_annoV1.1\\exp_in_meiosis\\")

library(WGCNA)

options(stringsAsFactors = FALSE)

allowWGCNAThreads(20)

#load data from the 1st part (see S2 Text)

lnames=load(file="filtered_data_HC_only_ready_for_WGCNA_0.5tpm.RData")

lnames

datExpr0[1:4,1:4]

datExpr <- datExpr0

rm(datExpr0)

datExpr[1:4,1:4]

dim(datExpr)

print("dimensions of datExpr after removing bad genes")

print(dim(datExpr))

powers = c(c(1:20))

sft = pickSoftThreshold(datExpr, powerVector = powers, verbose = 5, networkType ="signed hybrid",

corFnc ="bicor", corOptions=list(maxPOutliers=0.05))

#Plot the results:

sizeGrWindow(9, 5)

pdf(file="soft-threshold_power_signed_hybrid_HC_only_0.5tpm.pdf")

par(mfrow = c(1,2));

cex1 = 0.9;

# Scale-free topology fit index as a function of the soft-thresholding power

plot(sft$fitIndices[,1], -sign(sft$fitIndices[,3])*sft$fitIndices[,2],

xlab="Soft Threshold (power)",ylab="Scale Free Topology Model Fit,signed R^2",type="n",

main = paste("Scale independence"));

text(sft$fitIndices[,1], -sign(sft$fitIndices[,3])*sft$fitIndices[,2],

labels=powers,cex=cex1,col="red");

# this line corresponds to using an R^2 cut-off of h

abline(h=0.90,col="red")

# Mean connectivity as a function of the soft-thresholding power

plot(sft$fitIndices[,1], sft$fitIndices[,5],

xlab="Soft Threshold (power)",ylab="Mean Connectivity", type="n",

main = paste("Mean connectivity"))

text(sft$fitIndices[,1], sft$fitIndices[,5], labels=powers, cex=cex1,col="red")

dev.off()

**#Part 4: R script used to run the WGCNA analysis and MEs calculation and plotting**

setwd("G:\\ABDUL_KADER\\Meiosis_genes_in_wheat\\Gene_Networks\\WGCNA_annoV1.1\\HC_exp_in_meiosis\\")

library(WGCNA)

options(stringsAsFactors = FALSE)

allowWGCNAThreads(2)

lnames=load(file="filtered_data_HC_only_ready_for_WGCNA_0.5tpm.RData")

lnames

datExpr0[1:4,1:4]

datExpr <- datExpr0

rm(datExpr0)

datExpr[1:4,1:4]

print("dimensions of datExpr after removing bad genes")

print(dim(datExpr)) #[1] 130 60379

setwd("G:\\ABDUL_KADER\\Meiosis_genes_in_wheat\\Gene_Networks\\WGCNA_annoV1.1\\HC_exp_in_meiosis\\maxP0.05\\")

# This shows that for a signed hybrid network I should use a power of 7.

power <- 7

bwnet = blockwiseModules(datExpr, maxBlockSize = 46000,

power = power, networkType = "signed hybrid", TOMType = "unsigned", minModuleSize = 30, corType="bicor",

corOptions = "use = 'p', maxPOutliers = 0.05",

reassignThreshold = 0, mergeCutHeight = 0.15,

numericLabels = TRUE,

saveTOMs = TRUE,

saveTOMFileBase = "Signed_hybrid_TOM-blockwise_maxP0.05",

verbose = 3)

names(bwnet)

length(table(bwnet$blocks))

table(bwnet$blocks)

#get the modules colours

bwnetModuleColors <- labels2colors(bwnet$colors)

#get the module labels

bwnetModuleLabels <- bwnet$colors

#look at how many genes per module

table(bwnetModuleLabels)

bwnetModuleLabels

write.csv(table(bwnetModuleLabels), file="bwnet_modules_mergeCutHeight_0.15.csv")

#plot histogram of module sizes

pdf(file="Number of genes in module mergeCutHeight_0.15.pdf")

par(mfrow=c(1,2))

hist(table(bwnetModuleLabels), xlim=c(1,10000), breaks=1000, xlab="Number of genes in Module", main="mergeCutHeight_0.15")

hist(table(bwnetModuleLabels), xlim=c(1,1000), breaks=1000, xlab="Number of genes in Module", main="Zoomed in mergeCutHeight_0.15")

dev.off()

pdf(file="dendrogram_of_module_membership_0.15_mergeCutHeight.pdf", width=6, height=4)

for (i in 1:length(table(bwnet$blocks))) {

plotDendroAndColors(bwnet$dendrograms[[i]],

bwnetModuleColors[bwnet$blockGenes[[i]]],

"Module colors", main = paste0("Gene dendrogram and module colors in block ",i),

dendroLabels = FALSE, hang = 0.03, addGuide = TRUE, guideHang = 0.05)

}

dev.off()

save(bwnet, file="bwnet_network_mergeCutHeight0.15.RData")

bwnetMEs <- bwnet$MEs

bwnetdendrograms <- bwnet$dendrograms

save(bwnetModuleColors, bwnetModuleLabels, bwnetMEs, bwnetdendrograms, file = "bwnet_network_components_mergeCutHeight0.15.RData")

gene_expr <- cbind(t(datExpr),bwnetModuleLabels, bwnetModuleColors)

head(gene_expr)

# write genes with modules to file

write.csv(gene_expr,"genes_with_modules_mergeCutHeight0.15.csv")

#Calculate Module Eigengenes (MEs)

setwd("G:\\ABDUL_KADER\\Meiosis_genes_in_wheat\\Gene_Networks\\WGCNA_annoV1.1\\HC_exp_in_meiosis\\maxP0.05\\")

library(WGCNA)

options(stringsAsFactors = FALSE)

#calculate MEs

lnames=load(file="G:\\ABDUL_KADER\\Meiosis_genes_in_wheat\\Gene_Networks\\WGCNA_annoV1.1\\HC_exp_in_meiosis\\filtered_data_HC_only_ready_for_WGCNA_0.5tpm.Rdata")

# lnames contains the names of loaded variables

lnames

datExpr <- datExpr0

rm(datExpr0)

colnames(datExpr)

rownames(datExpr)

# Load network data saved before (see S3 Text)

lnames=load(file="bwnet_network_components_mergeCutHeight0.15.Rdata")

lnames

nGenes = ncol(datExpr)

nSamples = nrow(datExpr)

MEs = moduleEigengenes(datExpr, bwnetModuleLabels)$eigengenes

colnames(MEs)

rownames(MEs)

rownames(MEs) <- rownames(datExpr)

rownames(MEs)

head(MEs)

write.table(MEs, file="eigengenes.txt")

write.csv(MEs, file="eigengenes.csv")

# remove eigengene 0

MEs <- MEs[,2:ncol(MEs)]

head(MEs)

setwd("G:\\ABDUL_KADER\\Meiosis_genes_in_wheat\\Gene_Networks\\WGCNA_annoV1.1\\HC_exp_in_meiosis\\maxP0.05\\heatmap_eigengene\\")

#plot deodrogram of Eigengene relatedness

sizeGrWindow(10,10);

pdf(file="Eigengene dendrogram.pdf", width=14, height=7)

par(cex = 0.8)

plotEigengeneNetworks(MEs, "Eigengene dendrogram", marDendro = c(0,4,2,0),

plotHeatmaps = FALSE)

dev.off()

# make dist and hclust

distance <- dist(t(MEs), method = "euclidean")

head(distance)

hclust_distance <- hclust(distance, method = "complete", members = NULL)

head(hclust_distance)

plot(hclust_distance)

dev.off()

#with bootstraps

library(pvclust)

result <- pvclust((MEs), method.dist="cor", method.hclust="average", nboot=1000, iseed=2)

par(cex = 0.8)

plot(result, print.pv=TRUE, print.num=TRUE, float=0.01,

col.pv=c(2,3,5), cex.pv=0.5, font.pv=NULL, col=NULL, cex=0.8,

font=NULL, lty=NULL, lwd=NULL, main=NULL, sub=NULL, xlab=0.8)

pvrect(result, alpha=0.95)

pdf(file="eigengene_dendrogram_bootstrap1000_no_rectangle.pdf", width=14, height=7)

plot(result, print.pv=TRUE, print.num=TRUE, float=0.01,

col.pv=c(2,3,5), cex.pv=0.5, font.pv=NULL, col=NULL, cex=0.8,

font=NULL, lty=NULL, lwd=NULL, main=NULL, sub=NULL, xlab=0.8)

#pvrect(result, alpha=0.95)

dev.off()

#Plot MEs heatmap

setwd("G:\\ABDUL_KADER\\Meiosis_genes_in_wheat\\Gene_Networks\\WGCNA_annoV1.1\\HC_exp_in_meiosis\\maxP0.05\\heatmap_eigengene\\")

library(made4)

pdf(file="module_eigengene_expr_heatmap1.pdf", width =10, height=10)

heatplot(t(as.matrix(MEs)),margins=c(4,7),cexCol = 0.4, scale="column")

dev.off()

pdf(file="module_eigengene_expr_heatmap2.pdf", width =10, height=10)

heatplot(t(as.matrix(MEs)),margins=c(4,7),cexCol = 0.4, scale="none",dualScale=FALSE)

#### now want to plot heatmap of eigengene with bar of metadata #####

library("NMF")

metadata_file <- "G:\\ABDUL_KADER\\Meiosis_genes_in_wheat\\Gene_Networks\\WGCNA_annoV1.1\\metadata.csv"

metadata <- read.csv(metadata_file, row.names = 1, sep=",")

dim(metadata) # [1] 130 10

head(metadata)

colnames(metadata)

metadata[1:10,1:4]

matrix_to_plot <- t(as.matrix(MEs))

# remove ME0

matrix_to_plot <- matrix_to_plot[2:nrow(matrix_to_plot),]

rownames(matrix_to_plot)

# want to plot with order according to eigengene tree

rownames(matrix_to_plot)

matrix_to_plot <- matrix_to_plot[c(25,0,11,41,59,56,40,28,33,26,19,6,39,37,62,42,31,12,63,66,13,5,36,29,61,47,38,23,50,16,48,24,57,49,55,32,27,58,51,65,60,45,21,1,17,14,30,34,22,4,2,53,44,43,10,52,35,9,7,64,54,46,15,18,20,3,8),]

rownames(matrix_to_plot)

head(matrix_to_plot)

rownames(matrix_to_plot) <- gsub("ME","",rownames(matrix_to_plot))

# now get correct colours

Tissue <- c("#611aa6","#a61aa5", "#1a5fa6", "#deb42e","#3ca61a","#4e6410", "#645010", "#a6611a")

names(Tissue) <- c("Anthers_Meiocytes","Floral_parts","Spike","Grain","Leaves","Shoots","Stem","Roots")

ann_colors = list(Intermed.tissue = Tissue)

ann_colors

# order by intermed tissue

annotation_Intermed <- data.frame(metadata[,c(8)])

head(annotation_Intermed)

pdf(file=paste0("eigengene_heatmap_tissue__correct_row_order_coloured.pdf"), width = 12)

aheatmap(matrix_to_plot, annCol = annotation_Intermed, Colv=order(metadata[,8]),

annColors = ann_colors, Rowv = NA, scale="none", fontsize = 8, cexRow = 5)

dev.off()

#### now want to plot heatmap of eigengene with bar of metadata #####

metadata_file <- "G:\\ABDUL_KADER\\Meiosis_genes_in_wheat\\Gene_Networks\\WGCNA_annoV1.1\\metadata.csv"

metadata <- read.csv(metadata_file, row.names = 1, sep=",")

dim(metadata) # [1] 130 10

head(metadata)

colnames(metadata)

metadata[1:10,1:4]

print("nrow of metadata")

print(nrow(metadata)) # [1] 130

unique(metadata$Intermed.tissue)

# choose annotation for columns

annotation <- data.frame(metadata[,c(6:10)])

head(annotation)

**#Part 5: Calculate module-tissue correlation.**

setwd("G:\\ABDUL_KADER\\Meiosis_genes_in_wheat\\Gene_Networks\\WGCNA_annoV1.1\\HC_exp_in_meiosis\\maxP0.05\\")

eigengenes <- read.table(file="eigengenes.txt", header=T)

head(eigengenes)

# remove eigengene 0

eigengenes <- eigengenes[,2:ncol(eigengenes)]

head(eigengenes)

metadata_file <- "G:\\ABDUL_KADER\\Meiosis_genes_in_wheat\\Gene_Networks\\WGCNA_annoV1.1\\metadata_EI.txt"

metadata <- read.csv(metadata_file, row.names = 1, sep="\t")

dim(metadata)

head(metadata)

colnames(metadata)

dim(metadata_file)

metadata_Sel <- metadata[,c(2,8)]

head(metadata_Sel)

metadata_Sel$Sample.IDs <- gsub("-",".",metadata_Sel$Sample.IDs)

metadata_Sel$Sample.IDs == rownames(eigengenes)

colnames(metadata_Sel)

head(metadata_Sel)

unique(metadata_Sel$Intermed.tissue)

metadata_Sel$Meiotic_anther <- metadata_Sel$Intermed.tissue == "Meiotic_anther"

metadata_Sel$Leaves <- metadata_Sel$Intermed.tissue == "Leaves"

metadata_Sel$Roots <- metadata_Sel$Intermed.tissue == "Roots"

metadata_Sel$Grain <- metadata_Sel$Intermed.tissue == "Grain"

metadata_Sel$Shoots <- metadata_Sel$Intermed.tissue == "Shoots"

metadata_Sel$Stem <- metadata_Sel$Intermed.tissue == "Stem"

metadata_Sel$Spike <- metadata_Sel$Intermed.tissue == "Spike"

metadata_Sel$Floral_parts <- metadata_Sel$Intermed.tissue == "Floral_parts"

head(metadata_Sel,20)

tail(metadata_Sel,20)

setwd("G:\\ABDUL_KADER\\Meiosis_genes_in_wheat\\Gene_Networks\\WGCNA_annoV1.1\\HC_exp_in_meiosis\\maxP0.05\\tissue_module_correlations\\")

write.csv(metadata_Sel,file="Intermed_tissue_check_logical_tissue.csv")

cols <- sapply(metadata_Sel, is.logical)

metadata_Sel[,cols] <- lapply(metadata_Sel[,cols], as.numeric)

head(metadata_Sel)

rownames(metadata_Sel) <- metadata_Sel$Sample.IDs

dim(metadata_Sel)

metadata_logical <- metadata_Sel[,3:ncol(metadata_Sel)]

head(metadata_logical)

rownames(metadata_logical) ==rownames(eigengenes)

names(metadata_logical)

nSamples <- nrow(metadata_logical)

# now want to do a comparison between eigengenes and each column in metadata (i.e. intermed1 level stress)

library(WGCNA)

moduleTraitCor = cor(eigengenes, metadata_logical, use = "p")

moduleTraitPvalue = corPvalueStudent(moduleTraitCor, nSamples)

head(moduleTraitPvalue)

# FDR adjust pvalues

moduleTraitPvalue <- matrix(p.adjust(as.vector(as.matrix(moduleTraitPvalue)), method="BY"),ncol=ncol(moduleTraitPvalue))

head(moduleTraitPvalue)

rownames(moduleTraitPvalue) <- rownames(moduleTraitCor)

colnames(moduleTraitPvalue) <- colnames(moduleTraitCor)

head(moduleTraitPvalue)

# now display eigengenes to metadata relationships sizeGrWindow(15,6)

# Will display correlations and their p-values

textMatrix = paste(signif(moduleTraitCor, 2), "\n(", signif(moduleTraitPvalue, 1), ")", sep = "");

dim(textMatrix) = dim(moduleTraitCor)

par(mar = c(8, 8.5, 3, 3));

# Display the correlation values within a heatmap plot

labeledHeatmap(Matrix = moduleTraitCor, xLabels = names(metadata_logical), yLabels = names(eigengenes), ySymbols = names(eigengenes),

colorLabels = FALSE, colors = blueWhiteRed(50), textMatrix = textMatrix, setStdMargins = FALSE,

cex.text = 0.5, zlim = c(-1,1), main = paste("Module-tissue relationships"))

# now save as pdf and text matrix

write.csv(moduleTraitCor,file="Tissue_corr.csv")

write.csv(moduleTraitPvalue,file="Tissue_p-value.csv")

pdf(file="Tissue_corr_p-value_MEs1111.pdf", height=15, width=10)

par(mar = c(8, 8.5, 3, 3))

labeledHeatmap(Matrix = moduleTraitCor, xLabels = names(metadata_logical), yLabels = names(eigengenes), cex.lab.y = 0.75,

ySymbols = names(eigengenes), colorLabels = FALSE, colors = blueWhiteRed(50), textMatrix = textMatrix,

setStdMargins = FALSE, cex.text = 0.4, zlim = c(-1,1), main = paste("Module-tissue relationships"))

dev.off()

# now make pdf colour by significance

library("RColorBrewer")

hmcols<-colorRampPalette(c("red","white"))(256)

pdf(file="Tissue_corr_p-value_MEs_coloured_by_padj_0.05.pdf", height=15, width=10)

par(mar = c(8, 8.5, 3, 3))

labeledHeatmap(Matrix = moduleTraitPvalue, xLabels = names(metadata_logical), yLabels = names(eigengenes), cex.lab.y = 0.75, ySymbols = names(eigengenes), colorLabels = FALSE, colors = hmcols, textMatrix = textMatrix, setStdMargins = FALSE, cex.text = 0.45, zlim = c(0,0.05), main = paste("Module-tissue relationships"))

dev.off()

pdf(file="Tissue_corr_p-value_MEs_coloured_by_padj_0.01.pdf", height=15, width=10)

par(mar = c(8, 8.5, 3, 3))

labeledHeatmap(Matrix = moduleTraitPvalue, xLabels = names(metadata_logical), yLabels = names(eigengenes), cex.lab.y = 0.75, ySymbols = names(eigengenes), colorLabels = FALSE, colors = hmcols, textMatrix = textMatrix, setStdMargins = FALSE, cex.text = 0.45, zlim = c(0,0.01), main = paste("Module-tissue relationships"))

dev.off()

pdf(file="Tissue_corr_corr_p-value_MEs_coloured_by_padj_0.001.pdf", height=15, width=10)

par(mar = c(8, 8.5, 3, 3))

labeledHeatmap(Matrix = moduleTraitPvalue, xLabels = names(metadata_logical), yLabels = names(eigengenes), cex.lab.y = 0.75, ySymbols = names(eigengenes), colorLabels = FALSE, colors = hmcols, textMatrix = textMatrix, setStdMargins = FALSE, cex.text = 0.45, zlim = c(0,0.001), main = paste("Module-tissue relationships"))

dev.off()

**#Part 6: Enrichment analysis of GO and GO slim terms in the modules.**

#1# GO term enrichment analysis

setwd("G:\\ABDUL_KADER\\Meiosis_genes_in_wheat\\Gene_Networks\\WGCNA_annoV1.1\\HC_exp_in_meiosis\\maxP0.05\\")

module_info <- read.csv(file="genes_with_modules_mergeCutHeight0.15_no_expr_values.csv",

header=T)

head(module_info)

dim(module_info)

colnames(module_info) <- c("gene", "bwnetModulelabels", "bwnetModuleColors")

head(module_info)

dim(module_info)

rownames(module_info) <- module_info[,1]

module_info <- module_info[,-1]

head(module_info)

#read in information about lengths and GO terms for each cluster

OntologiesForGenes <- readRDS(file="G:\\ABDUL_KADER\\Meiosis_genes_in_wheat\\Gene_Networks\\WGCNA_annoV1.1\\OntologiesForGenes.rds")

head(OntologiesForGenes)

GO_IWGSC_Stress <- OntologiesForGenes[OntologiesForGenes$ontology =="IWGSC+Stress",]

all_go <- GO_IWGSC_Stress

head(all_go)

# first convert the v1.0 genes in all_go to v1.1 versions:

head(gsub("01G", "02G", all_go$Gene))

all_go$Gene <- (gsub("01G", "02G", all_go$Gene))

head(all_go)

dim(all_go) #[1] 1463101 3

# only keep genes which were >99 % ID > 90% coverage from v1.0 to v1.1

genes_to_transfer <- read.csv(file="G:\\ABDUL_KADER\\Meiosis_genes_in_wheat\\Gene_Networks\\WGCNA_annoV1.1\\genes_to_transfer_qcov90_pident99_same_ID.csv")

head(genes_to_transfer)

all_go <- all_go[all_go$Gene %in% genes_to_transfer$gene_v1.1,]

head(all_go)

all_go <- all_go[,c(1,2)]

colnames(all_go) <- c("Gene", "GO_id")

head(all_go)

dim(all_go)

# select only genes which were used for WGCNA

all_go <- subset(all_go, Gene %in% rownames(module_info))

dim(all_go)

#create vector for gene_lengths

lengths <- read.csv(file="G:\\ABDUL_KADER\\Meiosis_genes_in_wheat\\Gene_Networks\\WGCNA_annoV1.1\\gene_length.csv", header=T)

head(lengths)

colnames(lengths) <- c("gene", "length")

head(lengths)

t1 <- subset(lengths, gene %in% rownames(module_info))

head(t1)

dim(t1)

# turn into a vector called gene.lens to use with GOSeq

gene.lens <- as.numeric(t1$length)

names(gene.lens) = t1$gene

head(gene.lens)

#Do GO term enrichment and plot graph for each group ####

# start inspecting each cluster (this will save each hierachical group of genes and then perform GO term enrichment analysis using GOseq on each group

out_dir <- "G:\\ABDUL_KADER\\Meiosis_genes_in_wheat\\Gene_Networks\\WGCNA_annoV1.1\\HC_exp_in_meiosis\\maxP0.05\\GO_enrichment\\"

assayed.genes <- as.vector(rownames(module_info))

length(assayed.genes) #[1] 60379

unique(module_info$bwnetModulelabels)

library(goseq)

for (i in seq(1, length(unique(module_info$bwnetModulelabels)))) {

groupi_genes <- module_info[module_info$bwnetModulelabels == i, 1:2]

groupi_genes$target_id <- rownames(groupi_genes)

# decided not to write group genes file because I already have this info

write.table(groupi_genes, file = paste0(out_dir, "module_", i, "_genes.tsv", sep = ""), sep = "\t", quote = FALSE, col.names = TRUE)

de.genes <- groupi_genes$target_id

gene.vector=as.integer(assayed.genes%in%de.genes)

names(gene.vector)=assayed.genes

head(gene.vector)

#now carry out the GOseq analysis

pwf = nullp(gene.vector, bias.data = gene.lens, plot.fit = TRUE)

GO.wall = goseq(pwf, gene2cat = all_go, test.cats ="GO:BP")

#this gave table with p-values...now correct for multiple testing using FDR

enriched.GO=GO.wall$category[p.adjust(GO.wall$over_represented_pvalue, method="BH")<.05]

head(enriched.GO)

# add new column with over represented GO terms padj

GO.wall$over_rep_padj=p.adjust(GO.wall$over_represented_pvalue, method="BH")

# add new column with under represented GO terms padj

GO.wall$under_rep_padj=p.adjust(GO.wall$under_represented_pvalue, method="BH")

dep.GO=GO.wall$category[p.adjust(GO.wall$under_represented_pvalue, method="BH")<.05]

write.table(GO.wall, file = paste0(out_dir, "module_", i, "_GOseq.tsv", sep = ""), sep = "\t", quote = FALSE, col.names = TRUE)

}

# now extract GO over-representation info for revigo

setwd("G:\\ABDUL_KADER\\Meiosis_genes_in_wheat\\Gene_Networks\\WGCNA_annoV1.1\\HC_exp_in_meiosis\\maxP0.05\\GO_enrichment\\")

for (i in seq(1, length(unique(module_info$bwnetModulelabels)))) {

GO_data <- read.table(file = paste0(out_dir, "module_", i, "_GOseq.tsv", sep = ""), header=T, sep="\t")

head(GO_data)

dim(GO_data)

GO_data <- (GO_data[GO_data$over_rep_padj <0.05,c(1,8)])

dim(GO_data)

head(GO_data)

write.table(GO_data, file=paste0(out_dir, "module_", i, "_over_rep_GO_for_revigo.tsv", sep = ""), sep = "\t", quote=FALSE, col.names=F, row.names = F)

}

#2#GO slim term enrichment analysis

setwd("G:\\ABDUL_KADER\\Meiosis_genes_in_wheat\\Gene_Networks\\WGCNA_annoV1.1\\HC_exp_in_meiosis\\maxP0.05\\")

module_info <- read.csv(file="genes_with_modules_mergeCutHeight0.15_no_expr_values.csv",

header=T)

head(module_info)

dim(module_info)

colnames(module_info) <- c("gene", "bwnetModulelabels", "bwnetModuleColors")

head(module_info)

dim(module_info)

rownames(module_info) <- module_info[,1]

module_info <- module_info[,-1]

head(module_info)

#### read in information about lengths and GO terms for each cluster #########

OntologiesForGenes <- readRDS(file="G:\\ABDUL_KADER\\Meiosis_genes_in_wheat\\Gene_Networks\\WGCNA_annoV1.1\\OntologiesForGenes.rds")

head(OntologiesForGenes)

unique(OntologiesForGenes$ontology)

GO_IWGSC_Stress <- OntologiesForGenes[OntologiesForGenes$ontology =="slim_IWGSC+Stress",]

all_go <- GO_IWGSC_Stress

head(all_go)

# first convert the v1.0 genes in all_go to v1.1 versions:

head(gsub("01G", "02G", all_go$Gene))

all_go$Gene <- (gsub("01G", "02G", all_go$Gene))

head(all_go)

dim(all_go)

# only keep genes which were >99 % ID > 90% coverage from v1.0 to v1.1

genes_to_transfer <- read.csv(file="G:\\ABDUL_KADER\\Meiosis_genes_in_wheat\\Gene_Networks\\WGCNA_annoV1.1\\genes_to_transfer_qcov90_pident99_same_ID.csv")

head(genes_to_transfer)

all_go <- all_go[all_go$Gene %in% genes_to_transfer$gene_v1.1,]

head(all_go)

all_go <- all_go[,c(1,2)]

colnames(all_go) <- c("Gene", "GOslim_id")

head(all_go)

dim(all_go)

# select only genes which were used for WGCNA

all_go <- subset(all_go, Gene %in% rownames(module_info))

dim(all_go)

#create vector for gene_lengths

lengths <- read.csv(file="G:\\ABDUL_KADER\\Meiosis_genes_in_wheat\\Gene_Networks\\WGCNA_annoV1.1\\gene_length.csv", header=T)

head(lengths)

colnames(lengths) <- c("gene", "length")

head(lengths)

t1 <- subset(lengths, gene %in% rownames(module_info))

head(t1)

dim(t1)

# turn into a vector called gene.lens to use with GOSeq

gene.lens <- as.numeric(t1$length)

names(gene.lens) = t1$gene

head(gene.lens)

# Do GO term enrichment and plot graph for each group ####

# start inspecting each cluster (this will save each hierachical group of genes and then perform GO term enrichment analysis using GOseq on each group

out_dir <- "G:\\ABDUL_KADER\\Meiosis_genes_in_wheat\\Gene_Networks\\WGCNA_annoV1.1\\HC_exp_in_meiosis\\maxP0.05\\GO_enrichment\\"

assayed.genes <- as.vector(rownames(module_info))

length(assayed.genes) #[1] 60379

unique(module_info$bwnetModulelabels)

library(goseq)

for (i in seq(1, length(unique(module_info$bwnetModulelabels)))) {

groupi_genes <- module_info[module_info$bwnetModulelabels == i, 1:2]

groupi_genes$target_id <- rownames(groupi_genes)

de.genes <- groupi_genes$target_id

gene.vector=as.integer(assayed.genes%in%de.genes)

names(gene.vector)=assayed.genes

head(gene.vector)

#now carry out the GOseq analysis

pwf = nullp(gene.vector, bias.data = gene.lens, plot.fit = TRUE)

GO.wall = goseq(pwf, gene2cat = all_go, test.cats ="GO:BP")

#this gave table with p-values...now correct for multiple testing using FDR

enriched.GO=GO.wall$category[p.adjust(GO.wall$over_represented_pvalue, method="BH")<.05]

head(enriched.GO)

# add new column with over represented GO terms padj

GO.wall$over_rep_padj=p.adjust(GO.wall$over_represented_pvalue, method="BH")

# add new column with under represented GO terms padj

GO.wall$under_rep_padj=p.adjust(GO.wall$under_represented_pvalue, method="BH")

dep.GO=GO.wall$category[p.adjust(GO.wall$under_represented_pvalue, method="BH")<.05]

write.table(GO.wall, file = paste0(out_dir, "module_", i, "_GOslimseq.tsv", sep = ""), sep = "\t", quote = FALSE, col.names = TRUE)

}

# now extract GO over-representation info for revigo

setwd("G:\\ABDUL_KADER\\Meiosis_genes_in_wheat\\Gene_Networks\\WGCNA_annoV1.1\\HC_exp_in_meiosis\\maxP0.05\\GO_enrichment\\")

for (i in seq(1, length(unique(module_info$bwnetModulelabels)))) {

GO_data <- read.table(file = paste0(out_dir, "module_", i, "_GOslimseq.tsv", sep = ""), header=T, sep="\t")

head(GO_data)

dim(GO_data)

GO_data <- (GO_data[GO_data$over_rep_padj <0.05,c(1,8)])

dim(GO_data)

head(GO_data)

write.table(GO_data, file=paste0(out_dir, "module_", i, "_over_rep_GOslim_for_revigo.tsv", sep = ""), sep = "\t", quote=FALSE, col.names=F, row.names = F)

}

**#Part 7: Enrichment analysis of “Orthologs” and “Meiotic GO” genes in the modules.**

setwd("G:\\ABDUL_KADER\\Meiosis_genes_in_wheat\\Gene_Networks\\WGCNA_annoV1.1\\HC_exp_in_meiosis\\maxP0.05\\orthologs_2cat_FINAL\\")

module_info <- read.csv (file="G:\\ABDUL_KADER\\Meiosis_genes_in_wheat\\Gene_Networks\\WGCNA_annoV1.1\\HC_exp_in_meiosis\\maxP0.05\\genes_with_modules_mergeCutHeight0.15_no_expr_values.csv", header=T, sep=",")

head(module_info)

dim(module_info)

colnames(module_info) <- c("gene", "bwnetModulelabels", "bwnetModuleColors")

head(module_info)

dim(module_info)

gene_info <- read.csv(file="gene_info.csv", header=T)

head(gene_info)

dim(gene_info)

merged_info <- merge(gene_info,module_info, by.x="gene", by.y="gene")

head(merged_info)

write.csv(file="merged_info.csv", merged_info)

dim(merged_info)

unique(merged_info$category)

length(unique(merged_info$bwnetModulelabels)) #[1] 52 (so we have 15 out of 67 module with no genes assigned to them)

library("plyr")

cdata2 <- ddply(merged_info, .(category, bwnetModulelabels), summarise, Genes=length(gene), .drop=F)

head(cdata2)

head(cdata2[order(cdata2$bwnetModulelabels),])

dim(cdata2)

unique(cdata2$bwnetModulelabels)

write.csv(file="cdata2.csv", cdata2)

cdata <- read.csv(file="cdata.csv", header=T)

dim(cdata2)

dim(cdata)

colnames(cdata) <- c("category", "module", "number")

write.csv(file="merged_Genes_module_info.csv", cdata)

#remove module 0

cdata <- cdata[cdata$module != 0,]

head(cdata)

wide_data <- reshape(cdata,idvar="category",timevar="module", direction="wide")

write.csv(file="wide_data.csv", wide_data)

######### calculate fisher's exact test #########

head(module_info)

modules <- aggregate(module_info["gene"], by=module_info["bwnetModulelabels"], FUN=length)

head(modules)

dim(modules)

write.csv(file="number_of_genes_per_module.csv", modules)

modules <- modules[modules$bwnetModulelabels != 0,]

head(modules)

t_modules <- t(modules)

head(t_modules)

colnames(t_modules) <- t_modules[1,]

for (j in 1:2){

t_modules <- rbind(t_modules,t_modules[2,])

}

t_modules <- t_modules[2:3,]

head(t_modules)

rownames(t_modules) <- c(1:2)

head(t_modules)

head(merged_info)

agg_Genes <- aggregate(merged_info["gene"], by=merged_info[c("category")], FUN=length)

head(agg_Genes)

expr_genes <- nrow(module_info)

expr_genes

agg_Genes$perc_Genes <- agg_Genes$gene/expr_genes

head(agg_Genes)

expected_Genes <- t_modules*agg_Genes$perc_Genes

head(expected_Genes)

colnames(expected_Genes) <- paste0("number.",1:66)

expected_Genes <- data.frame(expected_Genes)

expected_Genes <- round(expected_Genes)

rownames(expected_Genes) <- agg_Genes$category

head(expected_Genes)

summary(expected_Genes)

write.csv(file="expected_Genes.csv", expected_Genes)

head(cdata)

cdata$number <- as.numeric(cdata$number) # need to make number numeric

cdata_agg <- aggregate(cdata["number"], by=cdata["category"], FUN=sum)

head(cdata_agg)

cdata_agg$perc <- cdata_agg$number/sum(cdata_agg$number)

head(cdata_agg)

dim(cdata_agg)

head(wide_data)

dim(wide_data)

Genes_in_module <- wide_data[,2:67]

rownames(Genes_in_module) <- wide_data[,1]

rownames(Genes_in_module)

colnames(Genes_in_module)

Genes_in_module <- cbind(Genes_in_module[,1:66])

head(Genes_in_module)

colnames(Genes_in_module) <- rep(paste0("module",(1:66)))

head(Genes_in_module)

write.csv(file="Genes_in_module.csv", Genes_in_module) # I checked all modules in the csv file

dim(Genes_in_module)

summary(Genes_in_module)

Genes_in_module <- data.frame(sapply(Genes_in_module, function(x) as.numeric(as.character(x))))

head(Genes_in_module)

summary(Genes_in_module)

head(Genes_in_module)

dim(Genes_in_module)

rownames(Genes_in_module) <- wide_data[,1]

head(Genes_in_module)

sum_of_Gene_per_module <- apply(Genes_in_module,2,sum, na.rm=T)

sum_of_Gene_per_module

Genes_in_module_mat <- as.matrix(Genes_in_module)

head(Genes_in_module_mat)

sum_of_Gene_per_module_vec <- as.vector(sum_of_Gene_per_module)

sum_of_Gene_per_module_vec

perc_Genes_per_module <- sweep(Genes_in_module_mat,2,sum_of_Gene_per_module_vec,`/`)

head(perc_Genes_per_module)

tail(perc_Genes_per_module)

dim(perc_Genes_per_module)

library(reshape2)

melted_perc_Genes_per_module <- melt(perc_Genes_per_module)

head(melted_perc_Genes_per_module)

colnames(melted_perc_Genes_per_module) <- c("category","module","perc")

dim(melted_perc_Genes_per_module)

unique(melted_perc_Genes_per_module$module)

melted_perc_Genes_per_module[melted_perc_Genes_per_module$module=="module66",]

head(cdata_agg)

head(melted_perc_Genes_per_module)

df.m <- merge(melted_perc_Genes_per_module, cdata_agg, by.x = "category", by.y = "category",suffixes =c(".raw",".mean"))

head(df.m)

tail(df.m)

dim(df.m)

library(ggplot2)

pdf(file="Genes_in_modules.pdf", height = 5, width =8)

ggplot(cdata, aes(x=category, y=number, fill=category)) + guides(fill=FALSE) + geom_bar(stat="identity", width =0.8) + coord_flip() +

scale_x_discrete(limits = rev(levels(as.factor(cdata$category)))) + theme_minimal() +

theme(axis.line= element_line(color="black")) + ggtitle("Genes_in_all_modules") + theme(axis.text.y = element_text(size=18)

+ theme(axis.text.x = element_text(size=18)))

dev.off()

head(cdata_agg)

dim(cdata_agg)

head(merged_info)

Gene_per_module <- ddply(merged_info, .(bwnetModulelabels), summarise, Genes=length(gene), .drop=F)

head(Gene_per_module)

Gene_per_module <- Gene_per_module[Gene_per_module$bwnetModulelabels != 0,]

head(Gene_per_module)

t_Gene_per_module <- t(Gene_per_module)

head(t_Gene_per_module)

colnames(t_Gene_per_module) <- t_Gene_per_module[1,]

for (i in 1:2){

t_Gene_per_module <- rbind(t_Gene_per_module,t_Gene_per_module[2,])

}

t_Gene_per_module <- t_Gene_per_module[2:3,]

rownames(t_Gene_per_module) <- c(1:2)

t_Gene_per_module <- as.data.frame(t_Gene_per_module)

head(t_Gene_per_module)

dim(t_Gene_per_module)

t_Gene_per_module$blank <- 0

head(t_Gene_per_module)

t_Gene_per_module <- cbind(t_Gene_per_module[,1:33], t_Gene_per_module$blank, t_Gene_per_module[,34:43], t_Gene_per_module$blank,

t_Gene_per_module[,44],t_Gene_per_module$blank, t_Gene_per_module$blank, t_Gene_per_module$blank, t_Gene_per_module[,45:46],

t_Gene_per_module$blank, t_Gene_per_module$blank, t_Gene_per_module[,47], t_Gene_per_module$blank,

t_Gene_per_module[,48], t_Gene_per_module$blank, t_Gene_per_module$blank, t_Gene_per_module[,49], t_Gene_per_module$blank,

t_Gene_per_module[,50:51], t_Gene_per_module$blank, t_Gene_per_module$blank, t_Gene_per_module$blank, t_Gene_per_module$blank)

colnames(t_Gene_per_module) <- c(1:66)

colnames(t_Gene_per_module)

perc_category_in_modules <- as.vector(cdata_agg$perc)

perc_category_in_modules

perc_Genes_per_module_correct <- sweep(as.matrix(t_Gene_per_module),1,perc_category_in_modules,`*`)

head(perc_Genes_per_module_correct)

rownames(perc_Genes_per_module_correct) <- cdata_agg$category

melted_perc_Genes_per_module_correct <- melt(perc_Genes_per_module_correct)

head(melted_perc_Genes_per_module_correct)

dim(melted_perc_Genes_per_module_correct) #[1] 132 3

colnames(melted_perc_Genes_per_module_correct) <- c("category","module","number")

melted_perc_Genes_per_module_correct$module <- gsub("module","",melted_perc_Genes_per_module_correct$module)

head(melted_perc_Genes_per_module_correct)

dim(melted_perc_Genes_per_module_correct) #[1] 132 3

head(cdata)

df.m2 <- merge(cdata, melted_perc_Genes_per_module_correct, by = c("category", "module"), suffixes =c(".actual",".expected"))

head(df.m2)

tail(df.m2)

#### fisher's exact test ####

head(df.m2)

head(modules)

modules_to_merge <- modules

colnames(modules_to_merge) <- c("module", "num_genes")

head(modules_to_merge)

dim(modules_to_merge)

df.m3 <- merge(df.m2, modules_to_merge, by = "module")

head(df.m3)

dim(df.m2) #[1] 396 4

dim(df.m3) #[1] 396 5

df.m3$genes_actual <- (df.m3$num_genes - df.m3$number.actual)

df.m3$genes_expected <- (df.m3$num_genes - df.m3$number.expected)

df.m3 <- df.m3[,-5]

df.m3 <- cbind(df.m3[,1:3],df.m3[,5], df.m3[,4], df.m3[,6])

head(df.m3)

colnames(df.m3) <- c("module", "category", "Gene_actual", "other_genes_actual", "Gene_expected", "other_genes_expected")

head(df.m3)

test.df <- matrix(df.m3[1,3:6], nrow=2)

test.df <-matrix(as.integer(test.df), nrow=2)

test.df

fisher_greater <- fisher.test(test.df, alternative="greater")

fisher_greater

fisher_less <- fisher.test(test.df, alternative="less")

fisher_less

fisher_greater$p.value #[1] 0.9979227

fisher_less$p.value #[1] 0.01058708

result <- c(as.character(df.m3[1,1]), as.character(df.m3[1,2]), fisher_greater$p.value, fisher_less$p.value )

result

result <- data.frame(module=character(), category=character(), p_value_greater=numeric(),p_value_less=numeric(), stringsAsFactors=F)

for (k in 1:nrow(df.m3)) {

test.df <- matrix(df.m3[k,3:6], nrow=2)

test.df <-matrix(as.integer(test.df), nrow=2)

fisher_greater <- fisher.test(test.df, alternative="greater")

fisher_less <- fisher.test(test.df, alternative="less")

result[nrow(result)+1,] <- c(as.character(df.m3[k,1]), as.character(df.m3[k,2]), fisher_greater$p.value, fisher_less$p.value)

}

head(result)

tail(result)

result$p_value_greater_adj <- p.adjust(result$p_value_greater, method="BY")

result$p_value_less_adj <- p.adjust(result$p_value_less, method="BY")

write.csv(file="fishers_exact_test_results.csv", result)

head(df.m2)

head(result)

df.m4 <- merge(df.m2, result, by.x = c("category","module"),by.y = c("category","module"))

head(df.m4)

df.m4$p_value_greater_sig <- df.m4$p_value_greater <0.05

df.m4$p_value_less_sig <- df.m4$p_value_less <0.05

head(df.m4)

df.m4$module <- factor(df.m4$module, levels=order(unique(df.m4$module))) # set order of module so that it goes from 1:66

head(df.m4)

write.csv(file="df.m4_data_for_plotting.csv", df.m4)

write.csv(file="df.m2_data_for_plotting.csv", df.m2)

library(ggplot2)

pdf(file="Gene_num_per_module_sig_highlighted_p_value.pdf", width=20, height=15)

myPlot <- ggplot(data=df.m4, aes(x=category)) +

scale_x_discrete(limits = (sort(levels(as.factor(df.m2$category))))) +

geom_bar(aes(y=number.actual, fill = interaction(p_value_greater_sig, p_value_less_sig)), stat="identity", width=0.8) +

scale_fill_manual(name="p-value < 0.05", values= c("grey","firebrick1","dodgerblue3"), labels=c("not significant", "enriched", "depleted")) +

facet_wrap(~ Module,scales="free_y", ncol=11) +

geom_point(aes(y=number.expected, group=1), color = "black",shape=23) +

theme_minimal() +

theme(axis.line= element_line(color="black")) + ggtitle("Genes_in_module") +

theme(axis.text.x=element_text(angle=90,hjust=1, size=8))

myPlot

dev.off()

#plot by category

pdf(file="Gene_num_per_category_sig_highlighted_p_value.pdf", width=15, height=6)

myPlot <- ggplot(data=df.m4, aes(x=module)) +

scale_x_discrete(limits = (sort(levels(as.factor(df.m2$Module))))) +

geom_bar(aes(y=number.actual, fill = interaction(p_value_greater_sig, p_value_less_sig)), stat="identity", width=0.8) +

scale_fill_manual(name="p-value < 0.05", values= c("grey","firebrick1","dodgerblue3"), labels=c("not significant", "enriched", "depleted")) +

facet_wrap(~ category,scales="free_y", ncol=1) +

geom_point(aes(y=number.expected, group=1), color = "black",shape=23) +

theme_minimal() +

theme(axis.line= element_line(color="black")) + ggtitle("Genes_in_modules per category") +

theme(axis.text.x=element_text(angle=90, size=12))

myPlot

dev.off()

head(cdata_agg)

dim(cdata_agg)

cdata_agg$perc_total <- cdata_agg$number/nrow(module_info)

head(cdata_agg)

head(t_modules)

dim(t_modules)

perc_category <- as.vector(as.numeric(cdata_agg$perc_total))

perc_category

expected_Genes_per_module_based_on_expr <- sweep(as.matrix(t_modules),1,perc_category,`*`)

head(expected_Genes_per_module_based_on_expr)

rownames(expected_Genes_per_module_based_on_expr) <- cdata_agg$category

melted_expected_Genes_per_module_based_on_expr <- melt(expected_Genes_per_module_based_on_expr)

head(melted_expected_Genes_per_module_based_on_expr)

colnames(melted_expected_Genes_per_module_based_on_expr) <- c("category","module","number")

melted_expected_Genes_per_module_based_on_expr$module <- gsub("module","",melted_expected_Genes_per_module_based_on_expr$module)

head(melted_expected_Genes_per_module_based_on_expr)

dim(melted_expected_Genes_per_module_based_on_expr) #[1] 198 3

head(cdata)

df.m2 <- merge(cdata, melted_expected_Genes_per_module_based_on_expr, by = c("category","module"), suffixes =c(".actual",".expected"))

head(df.m2)

tail(df.m2)

#### fisher's exact test ####

head(df.m2)

head(modules)

modules_to_merge <- modules

colnames(modules_to_merge) <- c("module", "num_genes")

head(modules_to_merge)

dim(modules_to_merge)

df.m3 <- merge(df.m2, modules_to_merge, by = "module")

head(df.m3)

dim(df.m2)

dim(df.m3)

df.m3$genes_actual <- (df.m3$num_genes - df.m3$number.actual)

df.m3$genes_expected <- (df.m3$num_genes - df.m3$number.expected)

df.m3 <- df.m3[,-5]

df.m3 <- cbind(df.m3[,1:3],df.m3[,5], df.m3[,4], df.m3[,6])

head(df.m3)

colnames(df.m3) <- c("module", "category", "Gene_actual", "other_genes_actual", "Gene_expected", "other_genes_expected")

head(df.m3)

test.df <- matrix(df.m3[1,3:6], nrow=2)

test.df <-matrix(as.integer(test.df), nrow=2)

test.df

fisher_greater <- fisher.test(test.df, alternative="greater")

fisher_greater

fisher_less <- fisher.test(test.df, alternative="less")

fisher_less

fisher_greater$p.value

fisher_less$p.value

result <- c(as.character(df.m3[1,1]), as.character(df.m3[1,2]), fisher_greater$p.value, fisher_less$p.value )

result

result <- data.frame(module=character(), category=character(), p_value_greater=numeric(),p_value_less=numeric(), stringsAsFactors=F)

result

for (k in 1:nrow(df.m3)) {

test.df <- matrix(df.m3[k,3:6], nrow=2)

test.df <-matrix(as.integer(test.df), nrow=2)

fisher_greater <- fisher.test(test.df, alternative="greater")

fisher_less <- fisher.test(test.df, alternative="less")

result[nrow(result)+1,] <- c(as.character(df.m3[k,1]), as.character(df.m3[k,2]), fisher_greater$p.value, fisher_less$p.value)

}

head(result)

tail(result)

result$p_value_greater_adj <- p.adjust(result$p_value_greater, method="BY")

result$p_value_less_adj <- p.adjust(result$p_value_less, method="BY")

write.csv(file="fishers_exact_test_results_all_expr_genes_background.csv", result)

head(df.m2)

head(result)

df.m4 <- merge(df.m2, result, by = c("category","module"))

head(df.m4)

df.m4$module <- factor(df.m4$module, levels=order(unique(df.m4$module))) # set order of module so that it goes from 1:66

df.m4$p_value_greater_sig <- df.m4$p_value_greater <0.05

df.m4$p_value_less_sig <- df.m4$p_value_less <0.05

head(df.m4)

df.m4$module <- factor(df.m4$module, levels=order(unique(df.m4$module))) # set order of module so that it goes from 1:39

library(ggplot2)

pdf(file="Gene_num_per_module_sig_highlighted_all_expr_background_p_value.pdf", width=15, height=10)

myPlot <- ggplot(data=df.m4, aes(x=category)) +

scale_x_discrete(limits = (sort(levels(as.factor(df.m2$category))))) +

geom_bar(aes(y=number.actual, fill = interaction(p_value_greater_sig, p_value_less_sig)), stat="identity", width=0.8) +

scale_fill_manual(name="p-value < 0.05", values= c("grey","firebrick1","dodgerblue3"), labels=c("not significant", "enriched", "depleted")) +

facet_wrap(~ Module,scales="free_y", ncol=11) +

geom_point(aes(y=number.expected, group=1), color = "black",shape=23) +

theme_minimal() +

theme(axis.line= element_line(color="black")) + ggtitle("Genes_in_module") +

theme(axis.text.x=element_text(angle=90,hjust=1, size=10))

myPlot

dev.off()

#plot by category

pdf(file="Gene_num_per_category_sig_highlighted_all_expr_background_p_value.pdf", width=12, height=4)

myPlot <- ggplot(data=df.m4, aes(x=module)) +

scale_x_discrete(limits = (levels(as.factor(df.m2$module)))) +

geom_bar(aes(y=number.actual, fill = interaction(p_value_greater_sig, p_value_less_sig)), stat="identity", width=0.8) +

scale_fill_manual(name="p-value < 0.05", values= c("grey","firebrick1","dodgerblue3"), labels=c("not significant", "enriched", "depleted")) +

facet_wrap(~ category,scales="free_y", ncol=1) +

geom_point(aes(y=number.expected, group=1), color = "black",shape=23) +

theme_minimal() +

theme(axis.line= element_line(color="black")) + ggtitle("Genes_in_modules per category") +

theme(axis.text.x=element_text(angle=90, size=10))

myPlot

dev.off()

# co_ord_flip

pdf(file="Gene_num_per_module_co_ord_flip_sig_highlighted_all_expr_background_p_value.pdf", width=12, height=12)

myPlot <- ggplot(data=df.m4, aes(x=category)) +

geom_bar(aes(y=number.actual, fill = interaction(p_value_greater_sig, p_value_less_sig)), stat="identity", width=0.8) +

scale_fill_manual(name="p-value < 0.05", values= c("grey","firebrick1","dodgerblue3"), labels=c("not significant", "enriched", "depleted")) +

facet_wrap(~ Module, scales="free_x",ncol=6) +

geom_point(aes(y=number.expected, group=1), color = "black",shape=20) +

scale_x_discrete(limits = rev(sort(levels(as.factor(df.m2$category))))) + theme_minimal() +

theme(axis.line= element_line(color="black")) + ggtitle("Genes_in_module") +

theme(axis.text.y=element_text(size=10)) +

theme(axis.text.x=element_text(size=10)) + coord_flip()

myPlot

dev.off()

#co_ord_flip plot by category

pdf(file="Gene_num_per_category_co_ord_flip_sig_highlighted_all_expr_background_p_value.pdf", width=6, height=15)

myPlot <- ggplot(data=df.m4, aes(x=module)) +

geom_bar(aes(y=number.actual, fill = interaction(p_value_greater_sig, p_value_less_sig)), stat="identity", width=0.8) +

scale_fill_manual(name="p-value < 0.05", values= c("grey","firebrick1","dodgerblue3"), labels=c("not significant", "enriched", "depleted")) +

facet_wrap(~ category, scales="free_x",ncol=3) +

geom_point(aes(y=number.expected, group=1), color = "black",shape=23) +

scale_x_discrete(limits = (levels(as.factor(df.m2$Module)))) + theme_minimal() +

theme(axis.line= element_line(color="black")) + ggtitle("Genes_in_modules per category") +

theme(axis.text.y=element_text(size=12)) +

theme(axis.text.x=element_text(size=12)) + coord_flip()

myPlot

dev.off()

**#Part 8: R script used to calculate hub genes for each module. Top 10 hub genes were selected for each module.**

setwd("G:\\ABDUL_KADER\\Meiosis_genes_in_wheat\\Gene_Networks\\WGCNA_annoV1.1\\HC_exp_in_meiosis\\maxP0.05\\top_kME\\")

library(WGCNA)

options(stringsAsFactors = FALSE)

lnames=load(file="G:\\ABDUL_KADER\\Meiosis_genes_in_wheat\\Gene_Networks\\WGCNA_annoV1.1\\HC_exp_in_meiosis\\filtered_data_HC_only_ready_for_WGCNA_0.5tpm.Rdata")

lnames

datExpr <- datExpr0

rm(datExpr0)

lnames=load(file="G:\\ABDUL_KADER\\Meiosis_genes_in_wheat\\Gene_Networks\\WGCNA_annoV1.1\\HC_exp_in_meiosis\\maxP0.05\\bwnet_network_components_mergeCutHeight0.15.Rdata")

lnames

nGenes = ncol(datExpr)

nSamples = nrow(datExpr)

#calculate hub genes (most highly connected = high kME values)

#read in module info

module_info <- read.csv (file="G:\\ABDUL_KADER\\Meiosis_genes_in_wheat\\Gene_Networks\\WGCNA_annoV1.1\\HC_exp_in_meiosis\\maxP0.05\\genes_with_modules_mergeCutHeight0.15_no_expr_values.csv", header=T, sep=",")

head(module_info)

dim(module_info)

modulesA1 <- as.vector(module_info$bwnetModuleColors)

PCs <- moduleEigengenes((datExpr), colors=modulesA1)

ME <- PCs$eigengenes

colorsA1 <- names(table(modulesA1))

colorsA1

# now get KME values

geneModuleMembership <- signedKME(datExpr,ME)

colnames(geneModuleMembership) <- paste0("PC", colorsA1,".cor")

head(geneModuleMembership)

MMPvalue1 <- corPvalueStudent(as.matrix(geneModuleMembership), dim(datExpr)[[2]])

colnames(MMPvalue1) <- paste0("PC",colorsA1,".pval")

head(MMPvalue1)

Gene <- colnames(datExpr)

head(Gene)

kMEtable1 <- cbind(Gene, Gene, modulesA1)

head(kMEtable1)

for (i in 1: length(colorsA1)){

kMEtable1 <- cbind(kMEtable1, geneModuleMembership[,i], MMPvalue1[,i] )

}

length(colorsA1)

head(kMEtable1)

colnames(kMEtable1) <- c("PSID","Gene", "Module", sort(c(colnames(geneModuleMembership),colnames(MMPvalue1))))

head(kMEtable1)

dim(kMEtable1)

write.csv(kMEtable1, "kMEtable.csv", row.names=F)

#find top ten hub genes per module head(geneModuleMembership)

topGenesKME <- NULL

for (c in 1:length(colorsA1)) {

kMErank1 <- rank(-geneModuleMembership[,c])

topGenesKME <- cbind(topGenesKME,Gene[kMErank1<=10])

}

head(sort(geneModuleMembership$PCblack.cor, decreasing=T))

topGenesKME

colnames(topGenesKME)

NULL

colorsA1

colnames(topGenesKME) <- colorsA1

topGenesKME

colnames(topGenesKME)

library("reshape2")

melted_topGenesKME <- melt(topGenesKME)

head(melted_topGenesKME)

melted_topGenesKME

# are the top genes TFs?

TFs <- read.csv(file="G:\\ABDUL_KADER\\Meiosis_genes_in_wheat\\Gene_Networks\\WGCNA_annoV1.1\\TFs_v1.1.csv")

head(TFs)

melted_topGenesKME_TF <- merge(melted_topGenesKME, TFs, by.x="value", by.y = "gene", all.x=T)

head(melted_topGenesKME_TF)

melted_topGenesKME_TF <- melted_topGenesKME_TF[,1:4]

head(melted_topGenesKME_TF)

melted_topGenesKME_TF <- melted_topGenesKME_TF[order(melted_topGenesKME_TF$Var2),]

head(melted_topGenesKME_TF,100)

# add in module numbers as well as colours

modules_info <- module_info[,2:3]

head(modules_info)

modules_info <- unique(modules_info)

head(modules_info)

melted_topGenesKME_TF_modNo <- merge(melted_topGenesKME_TF, modules_info, by.x ="Var2", by.y ="bwnetModuleColors" )

head(melted_topGenesKME_TF_modNo)

colnames(melted_topGenesKME_TF_modNo) <- c("bwnetModuleColors", "gene", "order", "TF_family", "bwnetModuleLabels")

head(melted_topGenesKME_TF_modNo)

melted_topGenesKME_TF_modNo <- melted_topGenesKME_TF_modNo[,c(2,5,1,3,4)]

head(melted_topGenesKME_TF_modNo)

write.csv(melted_topGenesKME_TF_modNo, file="top10_genes_kME_each_module.csv")

**#Part 9: Assessment of TF families in modules.**

setwd("G:\\ABDUL_KADER\\Meiosis_genes_in_wheat\\Gene_Networks\\WGCNA_annoV1.1\\HC_exp_in_meiosis\\maxP0.05\\TFs_in_modules\\")

# read in module info

module_info <- read.csv (file="G:\\ABDUL_KADER\\Meiosis_genes_in_wheat\\Gene_Networks\\WGCNA_annoV1.1\\HC_exp_in_meiosis\\maxP0.05\\genes_with_modules_mergeCutHeight0.15_no_expr_values.csv", header=T, sep=",")

head(module_info)

dim(module_info)

# rename columns

colnames(module_info) <- c("gene", "bwnetModulelabels", "bwnetModuleColors")

head(module_info)

dim(module_info)

# read in TF info

TF_info <- read.csv(file="G:\\ABDUL_KADER\\Meiosis_genes_in_wheat\\Gene_Networks\\WGCNA_annoV1.1\\TFs_v1.1.csv", header=T)

head(TF_info)

dim(TF_info)

merged_info <- merge(TF_info,module_info, by.x="gene", by.y="gene")

head(merged_info)

dim(merged_info)

unique(merged_info$TF_family)

length(unique(merged_info$bwnetModulelabels))

library("plyr")

cdata2 <- ddply(merged_info, .(TF_family, bwnetModulelabels), summarise, TFs=length(gene), .drop=F)

head(cdata2)

head(cdata2[order(cdata2$bwnetModulelabels),])

dim(cdata2)

#to know the missing modules (modules with no assigned TFs):

unique(cdata2$bwnetModulelabels)

unique(cdata2$TF_family)

# So, the missing 3 modules are: 54, 57, 59, 62

#df_mod_noTF <- data.frame(TF_family=c(rep(as.character(unique(cdata2$TF_family)))),

# bwnetModulelabels = c(rep(c("54","57","59","62"),length(unique(cdata2$TF_family)))),

# TFs = rep("0",length(unique(cdata2$TF_family))*4))

write.csv(file="cdata2.csv", cdata2)

#I added the missing modules manually to the file "cdata2.csv" and saved it in "cdata.csv"

cdata <- read.csv(file="cdata.csv", header=T)

# now combine

cdata <- rbind(cdata2, df_mod_noTF)

dim(cdata2)

dim(df_mod_noTF)

dim(cdata)

colnames(cdata) <- c("TF_family", "module", "number")

write.csv(file="merged_TF_module_info.csv", cdata)

#remove module 0

cdata <- cdata[cdata$module != 0,]

head(cdata)

wide_data <- reshape(cdata,idvar="TF_family",timevar="module", direction="wide")

######### calculate fisher's exact test #########

# first need to make a table of expected TFs per module using

# the proportion of TFs across all modules and number of genes in each module

# 1 calc no of genes in each module

head(module_info)

modules <- aggregate(module_info["gene"], by=module_info["bwnetModulelabels"], FUN=length)

head(modules)

dim(modules)

write.csv(file="number_of_genes_per_module.csv", modules)

#remove module 0

modules <- modules[modules$bwnetModulelabels != 0,]

head(modules)

t_modules <- t(modules)

head(t_modules)

colnames(t_modules) <- t_modules[1,]

# make t_modules into df with number of rows same as number of TFs (58)

for (j in 1:58){

t_modules <- rbind(t_modules,t_modules[2,])

}

t_modules <- t_modules[2:59,]

head(t_modules)

rownames(t_modules) <- c(1:58)

head(t_modules)

# 2 calculate average %TFs in each family across all expressed genes

head(merged_info)

agg_TF <- aggregate(merged_info["gene"], by=merged_info[c("TF_family")], FUN=length)

head(agg_TF)

expr_genes <- nrow(module_info)

expr_genes

agg_TF$perc_TF <- agg_TF$gene/expr_genes

head(agg_TF)

# 3 combine into table

expected_TF <- t_modules*agg_TF$perc_TF

head(expected_TF)

colnames(expected_TF) <- paste0("number.",1:66)

expected_TF <- data.frame(expected_TF)

expected_TF <- round(expected_TF)

rownames(expected_TF) <- agg_TF$TF_family

head(expected_TF)

summary(expected_TF)

write.csv(file="expected_TF.csv", expected_TF)

# 4 calculate average %TFs in each family across all modules

head(cdata)

cdata$number <- as.numeric(cdata$number) # need to make number numeric

cdata_agg <- aggregate(cdata["number"], by=cdata["TF_family"], FUN=sum)

head(cdata_agg)

cdata_agg$perc <- cdata_agg$number/sum(cdata_agg$number)

head(cdata_agg)

dim(cdata_agg)

#5 calc average % TFs per family in each module

head(wide_data)

dim(wide_data)

TF_in_module <- wide_data[,2:67]

rownames(TF_in_module) <- wide_data[,1]

rownames(TF_in_module)

colnames(TF_in_module)

# we need to arrand colomns by module number from 1 to 66

#first: arrange the colomns

TF_in_module <- cbind(TF_in_module[,1:66])

#TF_in_module <- cbind(TF_in_module[,1:53],TF_in_module[,63],TF_in_module[,54:55],TF_in_module[,64],

# TF_in_module[,56],TF_in_module[,65],TF_in_module[,57:58],TF_in_module[,66],TF_in_module[,59:62])

head(TF_in_module)

#second: change column name by module1 to module66

colnames(TF_in_module) <- rep(paste0("module",(1:66)))

head(TF_in_module)

write.csv(file="TF_in_module.csv", TF_in_module)

dim(TF_in_module)

summary(TF_in_module)

TF_in_module <- data.frame(sapply(TF_in_module, function(x) as.numeric(as.character(x)))) # make each column numeric

#(need to convert to character first to avoid 0 changing to 1)

head(TF_in_module)

summary(TF_in_module)

head(TF_in_module)

dim(TF_in_module)

rownames(TF_in_module) <- wide_data[,1]

head(TF_in_module)

sum_of_TF_per_module <- apply(TF_in_module,2,sum, na.rm=T)

sum_of_TF_per_module

TF_in_module_mat <- as.matrix(TF_in_module)

head(TF_in_module_mat)

sum_of_TF_per_module_vec <- as.vector(sum_of_TF_per_module)

sum_of_TF_per_module_vec

# divide number of TF in module by sum

perc_TF_per_module <- sweep(TF_in_module_mat,2,sum_of_TF_per_module_vec,`/`)

head(perc_TF_per_module)

tail(perc_TF_per_module)

dim(perc_TF_per_module)

library(reshape2)

melted_perc_TF_per_module <- melt(perc_TF_per_module)

head(melted_perc_TF_per_module)

colnames(melted_perc_TF_per_module) <- c("superfamily","module","perc")

dim(melted_perc_TF_per_module)

unique(melted_perc_TF_per_module$module)

melted_perc_TF_per_module[melted_perc_TF_per_module$module=="module66",]

#plot % TF with average

head(cdata_agg)

head(melted_perc_TF_per_module)

df.m <- merge(melted_perc_TF_per_module, cdata_agg, by.x = "superfamily", by.y = "TF_family",suffixes =c(".raw",".mean"))

head(df.m)

tail(df.m)

dim(df.m)

# plot graphs

library(ggplot2)

# plot across all modules

pdf(file="TF_in_all_modules.pdf", height = 5, width =5)

ggplot(cdata, aes(x=TF_family, y=number, fill=TF_family)) + guides(fill=FALSE) + geom_bar(stat="identity", width =0.8) + coord_flip() +

scale_x_discrete(limits = rev(levels(as.factor(cdata$TF_family)))) + theme_minimal() +

theme(axis.line= element_line(color="black")) + ggtitle("TF_in_all_modules") + theme(axis.text.y = element_text(size=5))

dev.off()

#### now plot with expected numbers too (expected adjusted for number of TF in module)#######

## this is to compare that give a module has X number of TFs, are the families in the distribution expected ####

head(cdata_agg) # has percentage of TFs across all modules per family

dim(cdata_agg)

# calc number of TF per module

head(merged_info)

TF_per_module <- ddply(merged_info, .(bwnetModulelabels), summarise, TFs=length(gene), .drop=F)

head(TF_per_module)

TF_per_module <- TF_per_module[TF_per_module$bwnetModulelabels != 0,]

head(TF_per_module)

t_TF_per_module <- t(TF_per_module)

head(t_TF_per_module)

colnames(t_TF_per_module) <- t_TF_per_module[1,]

for (i in 1:58){

t_TF_per_module <- rbind(t_TF_per_module,t_TF_per_module[2,])

}

t_TF_per_module <- t_TF_per_module[2:59,]

rownames(t_TF_per_module) <- c(1:58)

t_TF_per_module <- as.data.frame(t_TF_per_module)

head(t_TF_per_module) # has number of TF in each module as a dataframe with 58 rows (one row per TF family)

dim(t_TF_per_module)

t_TF_per_module$blank <- 0

head(t_TF_per_module)

t_TF_per_module <- cbind(t_TF_per_module[,1:53], t_TF_per_module$blank, t_TF_per_module[,54:55], t_TF_per_module$blank, t_TF_per_module[,56],

t_TF_per_module$blank, t_TF_per_module[,57:58], t_TF_per_module$blank, t_TF_per_module[,59:62])

colnames(t_TF_per_module) <- c(1:66)

colnames(t_TF_per_module)

perc_TF_family_in_modules <- as.vector(cdata_agg$perc)

perc_TF_family_in_modules

# divide number of TF in module by sum

perc_TF_per_module_correct <- sweep(as.matrix(t_TF_per_module),1,perc_TF_family_in_modules,`*`)

head(perc_TF_per_module_correct)

rownames(perc_TF_per_module_correct) <- cdata_agg$TF_family

melted_perc_TF_per_module_correct <- melt(perc_TF_per_module_correct)

head(melted_perc_TF_per_module_correct)

dim(melted_perc_TF_per_module_correct)

colnames(melted_perc_TF_per_module_correct) <- c("TF_family","module","number")

melted_perc_TF_per_module_correct$module <- gsub("module","",melted_perc_TF_per_module_correct$module)

head(melted_perc_TF_per_module_correct)

dim(melted_perc_TF_per_module_correct)

# use cdata for real values

head(cdata)

df.m2 <- merge(cdata, melted_perc_TF_per_module_correct, by = c("TF_family", "module"), suffixes =c(".actual",".expected"))

head(df.m2)

tail(df.m2)

#### fisher's exact test ####

# using df.m2 created with has actual and expected numbers of TF for each module for each TF family

head(df.m2)

head(modules)

modules_to_merge <- modules

colnames(modules_to_merge) <- c("module", "num_genes")

head(modules_to_merge)

dim(modules_to_merge)

df.m3 <- merge(df.m2, modules_to_merge, by = "module")

head(df.m3)

dim(df.m2)

dim(df.m3)

df.m3$genes_actual <- (df.m3$num_genes - df.m3$number.actual)

df.m3$genes_expected <- (df.m3$num_genes - df.m3$number.expected)

df.m3 <- df.m3[,-5]

df.m3 <- cbind(df.m3[,1:3],df.m3[,5], df.m3[,4], df.m3[,6])

head(df.m3)

colnames(df.m3) <- c("module", "superfamily", "TF_actual", "other_genes_actual", "TF_expected", "other_genes_expected")

head(df.m3)

#make matrix for Fisher's exact test

test.df <- matrix(df.m3[1,3:6], nrow=2)

test.df <-matrix(as.integer(test.df), nrow=2)

test.df

fisher_greater <- fisher.test(test.df, alternative="greater")

fisher_greater

fisher_less <- fisher.test(test.df, alternative="less")

fisher_less

fisher_greater$p.value

fisher_less$p.value

result <- c(as.character(df.m3[1,1]), as.character(df.m3[1,2]), fisher_greater$p.value, fisher_less$p.value )

result

# now loop through all possibilities

result <- data.frame(module=character(), superfamily=character(), p_value_greater=numeric(),p_value_less=numeric(), stringsAsFactors=F)

for (k in 1:nrow(df.m3)) {

test.df <- matrix(df.m3[k,3:6], nrow=2)

test.df <-matrix(as.integer(test.df), nrow=2)

fisher_greater <- fisher.test(test.df, alternative="greater")

fisher_less <- fisher.test(test.df, alternative="less")

result[nrow(result)+1,] <- c(as.character(df.m3[k,1]), as.character(df.m3[k,2]), fisher_greater$p.value, fisher_less$p.value)

}

head(result)

tail(result)

result$p_value_greater_adj <- p.adjust(result$p_value_greater, method="BY")

result$p_value_less_adj <- p.adjust(result$p_value_less, method="BY")

write.csv(file="fishers_exact_test_results.csv", result)

## now plot graph with significant bars highlighted

head(df.m2)

head(result)

df.m4 <- merge(df.m2, result, by.x = c("TF_family","module"),by.y = c("superfamily","module"))

head(df.m4)

# add columns for yes significant and no significant

df.m4$p_value_greater_sig <- df.m4$p_value_greater <0.05

df.m4$p_value_less_sig <- df.m4$p_value_less <0.05

head(df.m4)

df.m4$module <- factor(df.m4$module, levels=order(unique(df.m4$module))) # set order of module so that it goes from 1:66

head(df.m4)

write.csv(file="df.m4_data_for_plotting.csv", df.m4)

write.csv(file="df.m2_data_for_plotting.csv", df.m2)

#I sorted the modules from 1 to 66 manually in the two csv files then saved the modified file as:

#"mod.df.m2_data_for_plotting_background.csv" and "mod.df.m4_data_for_plotting_background.csv"

df.m2 <- read.csv (file="mod.df.m2_data_for_plotting.csv", header=T)

df.m4 <- read.csv (file="mod.df.m4_data_for_plotting.csv", header=T)

library(ggplot2)

pdf(file="TF_num_per_module_sig_highlighted.pdf", width=15, height=20)

myPlot <- ggplot(data=df.m4, aes(x=TF_family)) +

scale_x_discrete(limits = (sort(levels(as.factor(df.m2$TF_family))))) +

geom_bar(aes(y=number.actual, fill = interaction(p_value_greater_sig, p_value_less_sig)), stat="identity", width=0.8) +

scale_fill_manual(name="p-value < 0.05", values= c("grey","firebrick1","dodgerblue3"), labels=c("not significant", "enriched", "depleted")) +

facet_wrap(~ Module,scales="free_y", ncol=3) +

geom_point(aes(y=number.expected, group=1), color = "black",shape=20) +

theme_minimal() +

theme(axis.line= element_line(color="black")) + ggtitle("TF_in_module") +

theme(axis.text.x=element_text(angle=90,hjust=1, size=5))

myPlot

dev.off()

# co_ord_flip

pdf(file="TF_num_per_module_co_ord_flip_sig_highlighted.pdf", width=15, height=20)

myPlot <- ggplot(data=df.m4, aes(x=TF_family)) +

geom_bar(aes(y=number.actual, fill = interaction(p_value_greater_sig, p_value_less_sig)), stat="identity", width=0.8) +

scale_fill_manual(name="p-value < 0.05", values= c("grey","firebrick1","dodgerblue3"), labels=c("not significant", "enriched", "depleted")) +

facet_wrap(~ module, scales="free_x",ncol=6) +

geom_point(aes(y=number.expected, group=1), color = "black",shape=20) +

scale_x_discrete(limits = rev(sort(levels(as.factor(df.m2$TF_family))))) + theme_minimal() +

theme(axis.line= element_line(color="black")) + ggtitle("TF_in_module") +

theme(axis.text.y=element_text(size=4)) + coord_flip()

myPlot

dev.off()

####### plot only for meiosis-single module ########

#I selected the data manually for modules 2, 28 and 41 from "mod.df.m2_data_for_plotting" and "mod.df.m4_data_for_plotting" and saved

# them in the files "mod.df.m2_data_2_28_41_for_plotting" and "mod.df.m4_data_2_28_41_for_plotting" respectively.

df.m2 <- read.csv (file="mod.df.m2_data_2_28_41_for_plotting.csv", header=T)

df.m4 <- read.csv (file="mod.df.m4_data_2_28_41_for_plotting.csv", header=T)

library(ggplot2)

pdf(file="TF_num_per_modules_2_28_41_sig_highlighted.pdf", width=14, height=7)

myPlot <- ggplot(data=df.m4, aes(x=TF_family)) +

scale_x_discrete(limits = (sort(levels(as.factor(df.m2$TF_family))))) +

geom_bar(aes(y=number.actual, fill = interaction(p_value_greater_sig, p_value_less_sig)), stat="identity", width=0.8) +

scale_fill_manual(name="p-value < 0.05", values= c("grey","firebrick1","dodgerblue3"), labels=c("not significant", "enriched", "depleted")) +

facet_wrap(~ Module,scales="free_y", ncol=1) +

geom_point(aes(y=number.expected, group=1), color = "black",shape=23) +

theme_minimal() +

theme(axis.line= element_line(color="black")) + ggtitle("TF_in_module") +

theme(axis.text.x=element_text(angle=90,hjust=1, size=12)) +

theme(axis.text.y=element_text(size=12))

myPlot

dev.off()

# co_ord_flip

pdf(file="TF_num_per_module_2_28_41_co_ord_flip_sig_highlighted.pdf", width=8, height=10)

myPlot <- ggplot(data=df.m4, aes(x=TF_family)) +

geom_bar(aes(y=number.actual, fill = interaction(p_value_greater_sig, p_value_less_sig)), stat="identity", width=0.8) +

scale_fill_manual(name="p-value < 0.05", values= c("grey","firebrick1","dodgerblue3"), labels=c("not significant", "enriched", "depleted")) +

facet_wrap(~ Module, scales="free_x",ncol=3) +

geom_point(aes(y=number.expected, group=1), color = "black",shape=23) +

scale_x_discrete(limits = rev(sort(levels(as.factor(df.m2$TF_family))))) + theme_minimal() +

theme(axis.line= element_line(color="black")) + ggtitle("TF_in_module") +

theme(axis.text.x=element_text(size=12) +

theme(axis.text.y=element_text(size=12))) + coord_flip()

myPlot

dev.off()

#now plot with expected numbers too (expected adjusted for number of TF in module)

#this is to compare whether each TF family in each module is the same % of that TF as would be expected across all expressed genes

head(cdata_agg) # has percentage of TFs across all modules per family

dim(cdata_agg)

cdata_agg$perc_total <- cdata_agg$number/nrow(module_info)

head(cdata_agg)

# has percentage of TFs in module

head(t_modules) # has number of genes in each module as a dataframe with 58 rows (one row per TF family)

dim(t_modules)

perc_TF_family <- as.vector(as.numeric(cdata_agg$perc_total))

perc_TF_family

# multiply number of genes in module by % TF expected across all genes

expected_TF_per_module_based_on_expr <- sweep(as.matrix(t_modules),1,perc_TF_family,`*`)

head(expected_TF_per_module_based_on_expr)

rownames(expected_TF_per_module_based_on_expr) <- cdata_agg$TF_family

melted_expected_TF_per_module_based_on_expr <- melt(expected_TF_per_module_based_on_expr)

head(melted_expected_TF_per_module_based_on_expr)

colnames(melted_expected_TF_per_module_based_on_expr) <- c("TF_family","module","number")

melted_expected_TF_per_module_based_on_expr$module <- gsub("module","",melted_expected_TF_per_module_based_on_expr$module)

head(melted_expected_TF_per_module_based_on_expr)

dim(melted_expected_TF_per_module_based_on_expr) #[1] 2262 3

# use cdata for real values

head(cdata)

df.m2 <- merge(cdata, melted_expected_TF_per_module_based_on_expr, by = c("TF_family","module"), suffixes =c(".actual",".expected"))

head(df.m2)

tail(df.m2)

#### fisher's exact test ####

# using df.m2 created which has actual and expected numbers of TF for each module for each TF family

head(df.m2)

head(modules)

modules_to_merge <- modules

colnames(modules_to_merge) <- c("module", "num_genes")

head(modules_to_merge)

dim(modules_to_merge)

df.m3 <- merge(df.m2, modules_to_merge, by = "module")

head(df.m3)

dim(df.m2)

dim(df.m3)

df.m3$genes_actual <- (df.m3$num_genes - df.m3$number.actual)

df.m3$genes_expected <- (df.m3$num_genes - df.m3$number.expected)

df.m3 <- df.m3[,-5]

df.m3 <- cbind(df.m3[,1:3],df.m3[,5], df.m3[,4], df.m3[,6])

head(df.m3)

colnames(df.m3) <- c("module", "TF_family", "TF_actual", "other_genes_actual", "TF_expected", "other_genes_expected")

head(df.m3)

#make matrix for Fisher's exact test # here on 38.6.2017 13:16

test.df <- matrix(df.m3[1,3:6], nrow=2)

test.df <-matrix(as.integer(test.df), nrow=2)

test.df

fisher_greater <- fisher.test(test.df, alternative="greater")

fisher_greater

fisher_less <- fisher.test(test.df, alternative="less")

fisher_less

fisher_greater$p.value

fisher_less$p.value

result <- c(as.character(df.m3[1,1]), as.character(df.m3[1,2]), fisher_greater$p.value, fisher_less$p.value )

result

# now loop through all possibilities

result <- data.frame(module=character(), TF_family=character(), p_value_greater=numeric(),p_value_less=numeric(), stringsAsFactors=F)

result

for (k in 1:nrow(df.m3)) {

test.df <- matrix(df.m3[k,3:6], nrow=2)

test.df <-matrix(as.integer(test.df), nrow=2)

fisher_greater <- fisher.test(test.df, alternative="greater")

fisher_less <- fisher.test(test.df, alternative="less")

result[nrow(result)+1,] <- c(as.character(df.m3[k,1]), as.character(df.m3[k,2]), fisher_greater$p.value, fisher_less$p.value)

}

head(result)

tail(result)

result$p_value_greater_adj <- p.adjust(result$p_value_greater, method="BY")

result$p_value_less_adj <- p.adjust(result$p_value_less, method="BY")

write.csv(file="fishers_exact_test_results_all_expr_genes_background.csv", result)

## now plot graph with significant bars highlighted

head(df.m2)

head(result)

df.m4 <- merge(df.m2, result, by = c("TF_family","module"))

head(df.m4)

# add columns for yes significant and no significant

df.m4$p_value_greater_sig <- df.m4$p_value_greater <0.05

df.m4$p_value_less_sig <- df.m4$p_value_less <0.05

head(df.m4)

df.m4$module <- factor(df.m4$module, levels=order(unique(df.m4$module))) # set order of module so that it goes from 1:39

write.csv(file="df.m4_data_for_plotting_background.csv", df.m4)

write.csv(file="df.m2_data_for_plotting_background.csv", df.m2)

#I sorted the modules from 1 to 66 manually in the two csv files then saved the modified file as:

#"mod.df.m2_data_for_plotting_background.csv" and "mod.df.m4_data_for_plotting_background.csv"

df.m2 <- read.csv (file="mod.df.m2_data_for_plotting_background.csv", header=T)

df.m4 <- read.csv (file="mod.df.m4_data_for_plotting_background.csv", header=T)

library(ggplot2)

pdf(file="TF_num_per_module_sig_highlighted_all_expr_background1.pdf", width=15, height=20)

myPlot <- ggplot(data=df.m4, aes(x=TF_family)) +

scale_x_discrete(limits = (sort(levels(as.factor(df.m2$TF_family))))) +

geom_bar(aes(y=number.actual, fill = interaction(p_value_greater_sig, p_value_less_sig)), stat="identity", width=0.8) +

scale_fill_manual(name="p-value < 0.05", values= c("grey","firebrick1","dodgerblue3"), labels=c("not significant", "enriched", "depleted")) +

facet_wrap(~ Module,scales="free_y", ncol=3) +

geom_point(aes(y=number.expected, group=1), color = "black",shape=20) +

theme_minimal() +

theme(axis.line= element_line(color="black")) + ggtitle("TF_in_module") +

theme(axis.text.x=element_text(angle=90,hjust=1, size=5))

myPlot

dev.off()

# co_ord_flip

pdf(file="TF_num_per_module_co_ord_flip_sig_highlighted_all_expr_background.pdf", width=15, height=20)

myPlot <- ggplot(data=df.m4, aes(x=TF_family)) +

geom_bar(aes(y=number.actual, fill = interaction(p_value_greater_sig, p_value_less_sig)), stat="identity", width=0.8) +

scale_fill_manual(name="p-value < 0.05", values= c("grey","firebrick1","dodgerblue3"), labels=c("not significant", "enriched", "depleted")) +

facet_wrap(~ module, scales="free_x",ncol=6) +

geom_point(aes(y=number.expected, group=1), color = "black",shape=20) +

scale_x_discrete(limits = rev(sort(levels(as.factor(df.m2$TF_family))))) + theme_minimal() +

theme(axis.line= element_line(color="black")) + ggtitle("TF_in_module") +

theme(axis.text.y=element_text(size=4)) + coord_flip()

myPlot

dev.off()

# plot only meiosis-related modules

#I selected the data for modules 2, 28 and 41 from "mod.df.m2_data_for_plotting" and "mod.df.m4_data_for_plotting" and saved

# them in the files "mod.df.m2_data_2_28_41_for_plotting" and "mod.df.m4_data_2_28_41_for_plotting" respectively.

df.m2 <- read.csv (file="mod.df.m2_data_2_28_41_for_plotting_background.csv", header=T)

df.m4 <- read.csv (file="mod.df.m4_data_2_28_41_for_plotting_background.csv", header=T)

library(ggplot2)

pdf(file="TF_num_per_modules_2_28_41_sig_highlighted_all_expr_background.pdf", width=14, height=7)

myPlot <- ggplot(data=df.m4, aes(x=TF_family)) +

scale_x_discrete(limits = (sort(levels(as.factor(df.m2$TF_family))))) +

geom_bar(aes(y=number.actual, fill = interaction(p_value_greater_sig, p_value_less_sig)), stat="identity", width=0.8) +

scale_fill_manual(name="p-value < 0.05", values= c("grey","firebrick1","dodgerblue3"), labels=c("not significant", "enriched", "depleted")) +

facet_wrap(~ Module,scales="free_y", ncol=1) +

geom_point(aes(y=number.expected, group=1), color = "black",shape=23) +

theme_minimal() +

theme(axis.line= element_line(color="black")) + ggtitle("TF_in_module") +

theme(axis.text.x=element_text(angle=90,hjust=1, size=12)) +

theme(axis.text.y=element_text(size=12))

myPlot

dev.off()

# co_ord_flip

pdf(file="TF_num_per_module_2_28_41_co_ord_flip_sig_highlighted_all_expr_background.pdf", width=8, height=10)

myPlot <- ggplot(data=df.m4, aes(x=TF_family)) +

geom_bar(aes(y=number.actual, fill = interaction(p_value_greater_sig, p_value_less_sig)), stat="identity", width=0.8) +

scale_fill_manual(name="p-value < 0.05", values= c("grey","firebrick1","dodgerblue3"), labels=c("not significant", "enriched", "depleted")) +

facet_wrap(~ Module, scales="free_x",ncol=3) +

geom_point(aes(y=number.expected, group=1), color = "black",shape=23) +

scale_x_discrete(limits = rev(sort(levels(as.factor(df.m2$TF_family))))) + theme_minimal() +

theme(axis.line= element_line(color="black")) + ggtitle("TF_in_module") +

theme(axis.text.x=element_text(size=12) +

theme(axis.text.y=element_text(size=12))) + coord_flip()

myPlot

dev.off()

**#Part 10: Calculate homeolog expression patterns in triads (original script).**

#First Part: This is the original script used by Ramírez-González et al. [36].

#the script was used to calculate homeolog expression patterns in triads across the 8 tissue types.

#Triad is considered expressed when min sum the expression of the three homeologs > 0.5 TPM

library(sqldf)

library(ggplot2)

library(reshape2)

library(fields)

library(gridExtra)

library(ggtern)

library(clue)

library(geometry)

setwd("G:\\ABDUL_KADER\\Meiosis_genes_in_wheat\\Gene_Networks\\WGCNA_annoV1.1\\CalculateTriadCategory_sum_original\\")

metadata<-read.csv("metadata.txt", row.names = 1, sep="\t")

nrow(metadata)

head(metadata)

loadValuesFromExperiment<-function(metadata, folder, unit="tpm", values=c("Aneuploidy")){

metadata$Sample.IDs <- gsub("-",".",metadata$Sample.IDs)

v<-values[1]

v<-gsub(" ","_",v)

v<-gsub(",",".",v)

path<-paste0(folder,"\\",v,"_",unit,".tsv")

ret<-read.table(path, row.names = 1, header= TRUE)

if(length(values) > 1){

for(i in 2:length(values)){

v<-values[i]

v<-gsub(" ","_",v)

v<-gsub(",",".",v)

path<-paste0(folder,"\\",v,"_",unit,".tsv")

tmp<-read.table(path, row.names = 1, header= TRUE)

ret<-cbind(ret,tmp)

}

}

md<-metadata[metadata$Sample.IDs%in%colnames(ret),]

ret<-ret[,as.character(md$Sample.IDs),]

list(ret,md)

}

folder<-"expressionValuesPerGene"

tpms <-loadValuesFromExperiment(metadata, folder, unit="tpm", values=unique(metadata$study.title))

metadata_used<-tpms[[2]]

tpms<-tpms[[1]]

nrow(metadata_used)

#Loading the homologies

homologies<-read.csv("HCTriads.csv", sep=",")

head(homologies)

nrow(homologies)

expressed_genes<-read.csv("expressed_genes_HC_tpmOver0.5AtLeast1sample_130samples.csv")

dim(expressed_genes)

head(expressed_genes)

genes_to_use <- data.frame(gene=expressed_genes$gene)

head(genes_to_use)

nrow(genes_to_use)

getSamplesForFactor<- function(metadata, type="Intermed.tissue",factor="Meiotic_anther"){

ret<-""

if(type != "all"){

ret<-as.character(metadata[metadata[,type] == factor,]$Sample.IDs)

}else{

ret<-as.character(unique(metadata$Sample.IDs))

}

ret

}

getMeansPerFactor<- function(values, metadata, type="Intermed.tissue",factor="Meiotic_anther"){

samples <- getSamplesForFactor(metadata, type, factor)

vals <- values[,samples]

mean<-0

if(length(samples) == 1){

print("This factor only has one sample!")

print(factor)

mean<-vals

}else{

mean<-rowMeans(vals)

}

mean<-sort(mean,decreasing=T)

cumulative <- cumsum(mean)

cumulative<-data.frame(cumulative)

mean<-data.frame(mean)

mean$gene <- rownames(mean)

cumulative$gene <- rownames(cumulative)

mean$total_samples <- length(samples)

n <-merge(mean,cumulative, by='gene', all=T)

n <- n[order(n$cumulative,decreasing = F),]

n$seq <- seq(from = 1, to = nrow(n))

n$factor = factor

n

}

getMeansForAllFactors<-function(values, metadata, type="Intermed.tissue"){

factors<-unique(metadata[,type])

f<-factors[1]

meansDFs <- getMeansPerFactor(tpms,metadata,type=type, factor=f)

for (i in 2:length(factors)){

f<-factors[i]

localDF<-getMeansPerFactor(tpms,metadata,type=type, factor=f)

meansDFs <- rbind(meansDFs,localDF)

}

meansDFs

}

isExpressedPerFactor <- function(values, metadata, type="Intermed.tissue",factor="Meiotic_anther", minTPM=0.5){

samples <- getSamplesForFactor(metadata, type, factor)

vals <- values[,samples]

means <- rowMeans(vals)

expr <- means > minTPM

m2 <- data.frame( expressed = expr)

m2$factor<-factor

m2$transcript<-rownames(m2)

m2$total_samples <- length(samples)

m2

}

getExclusiveExpression<-function(values, metadata, minTPM=0.5, type="Intermed.tissue"){

means <- getMeansForAllFactors(values, metadata,type=type)

means$expressed<-means$mean > minTPM

exclusiveExpresison<-sqldf("SELECT gene, factor, mean, total_samples

FROM means

WHERE expressed

GROUP BY gene HAVING count(factor) = 1 ")

list(means, exclusiveExpresison )

}

reshape_triad_groups<-function(triads){

values<-data.frame(gene=triads$A, chr_group="A", group_id=triads$group_id)

values<-rbind(values,data.frame(gene=triads$B, chr_group="B", group_id=triads$group_id))

values<-rbind(values,data.frame(gene=triads$D, chr_group="D", group_id=triads$group_id))

values

}

get_triad_matrix<-function(normalised_triad, factor="all"){

tmp<-normalised_triad[factor==normalised_triad$factor,]

tmp<-acast(tmp, group_id~chr_group, value.var="normalised_triad")

tmp<-tmp[complete.cases(tmp),]

tmp

}

plot_normalized_triads<-function(triads){

p <- ggplot(triads, aes(chr_group, normalised_triad))

p <- p + geom_boxplot(outlier.alpha = 0.05)

p <- p + ylab("Contribution") + xlab("Chromosome group")

p

}

get_centroids<-function(clust_df){

clusters<-sort(unique(clust_df$clust))

n_clust<-length(clusters)

dat <- data.frame(

A=numeric(n_clust),B=numeric(n_clust), D=numeric(n_clust), size=numeric(n_clust),

clust=numeric(n_clust), description=character(n_clust),stringsAsFactors=FALSE )

for(c in clusters){

tmp_df_clust<-clust_df[clust_df$clust==c,]

dat[c,1] <- mean(tmp_df_clust$A)

dat[c,2] <- mean(tmp_df_clust$B)

dat[c,3] <- mean(tmp_df_clust$D)

dat[c,4] <- nrow(tmp_df_clust)

dat[c,5] <- c

dat[c,6] <- paste0("other.",c)

}

rownames(dat)<-clusters

dat

}

plot_clust_dist<-function(clust_df, title="All"){

tmp_df<-clust_df[,c("A","B","D","group_id","clust","description")]

tmp_df<-melt(tmp_df,id=c("group_id","clust","description"))

colnames(tmp_df)<-c("group_id","clust","description","chr_group","normalised_triad")

clusters<-sort(unique(tmp_df$description))

tern <- ggtern(clust_df,aes(A,B,D,color=description, alpha=0.01)) +

geom_point() + theme_legend_position(x = "topleft") + scale_alpha(guide = 'none')

gs<-list(tern)

dat <- data.frame(

A=numeric(0),B=numeric(0), D=numeric(0), size=numeric(0),stringsAsFactors=FALSE )

rownames(dat)<-rownames(clusters)

for(c in clusters){

tmp_df_clust<-tmp_df[tmp_df$description==c,]

p<-plot_normalized_triads(tmp_df_clust)

p<- p + ylim(0,1)

p<- p + ylab("") + xlab("")

p<- p + ggtitle(c)

dat[c,1] <- round(100*mean(tmp_df_clust[tmp_df_clust$chr_group=="A","normalised_triad"]),digits=2)

dat[c,2] <- round(100*mean(tmp_df_clust[tmp_df_clust$chr_group=="B","normalised_triad"]),digits=2)

dat[c,3] <- round(100*mean(tmp_df_clust[tmp_df_clust$chr_group=="D","normalised_triad"]),digits=2)

dat[c,4] <- nrow(tmp_df_clust)

gs[[length(gs)+1]] <- p

}

total_size<-sum(dat$size)

dat$percentage<-round(100*dat$size/total_size,digits=2)

gs[[length(gs)+1]]<-tableGrob(dat)

lay <- rbind(c( 1, 1, 1, 2, 4, 7),

c( 1, 1, 1, 3, 5, 8),

c( 9, 9, 9, 6,NA,NA)

)

g2 <- arrangeGrob(grobs = gs, layout_matrix = lay, top = title)

g2

}

get_means_df<-function(metadata, tpms, type="Intermed.tissue", min_mean_tpm=0.5){

samples<-getSamplesForFactor(metadata, type="all",factor="all")

values<-data.frame(value=numeric(nrow(tpms)),stringsAsFactors=FALSE)

if(length(samples) > 1){

print("All samples:")

#print(samples)

#print(colnames(tpms))

values$value<-rowMeans(tpms[,samples])

}else{

values$value<-tpms[,samples]

}

values$factor<-"all"

values$gene<-rownames(tpms)

values$samples<-length(samples)

print(unique(metadata[,type]))

for(f in as.character(unique(metadata[,type]))){

print(f)

samples<-getSamplesForFactor(metadata, type=type,factor=f)

tmp<-data.frame(value=numeric(nrow(tpms)),stringsAsFactors=FALSE)

if(length(samples) > 1){

tmp$value<-rowMeans(tpms[,samples])

}else{

tmp$value<-tpms[,samples]

}

tmp$factor<-f

tmp$gene<-rownames(tpms)

tmp$samples<-length(samples)

print(colnames(values))

print(colnames(tmp))

values<-rbind(values,tmp)

}

casted<-dcast(values, gene~factor, value.var="value")

casted$all<-NULL

rownames(casted)<-casted$gene

casted$gene<-NULL

casted<-as.matrix(casted)

print(head(casted))

tmp<-data.frame(value=numeric(nrow(casted)),stringsAsFactors=FALSE)

tmp$value<-rowMeans(casted)

tmp$factor<-"all_means"

tmp$gene<-rownames(casted)

tmp$samples<-ncol(casted)

print("...")

print(colnames(values))

print(colnames(tmp))

values<-rbind(values,tmp)

print(head(casted))

print(head(min_mean_tpm))

casted<-ifelse(casted < min_mean_tpm, NA, casted)

print(head(casted))

tmp<-data.frame(value=numeric(nrow(casted)),stringsAsFactors=FALSE)

tmp$value<-rowMeans(casted, na.rm = TRUE)

tmp$factor<-"all_mean_filter"

tmp$gene<-rownames(casted)

tmp$samples<-rowSums(!is.na(casted))

print("...")

print(colnames(values))

print(colnames(tmp))

values<-rbind(values,tmp)

values

}

get_means_df<-function(metadata, tpms, triads, type="Intermed.tissue"){

samples<-getSamplesForFactor(metadata, type="all",factor="all")

values<-data.frame(value=numeric(nrow(tpms)),stringsAsFactors=FALSE)

if(length(samples) > 1){

values$value<-rowMeans(tpms[,samples])

}else{

values$value<-tpms[,samples]

}

values$factor<-"all"

values$gene<-rownames(tpms)

values$samples<-length(samples)

#print(unique(metadata[,type]))

for(f in unique(metadata[,type])){

#print(f)

samples<-getSamplesForFactor(metadata, type=type,factor=f)

tmp<-data.frame(value=numeric(nrow(tpms)),stringsAsFactors=FALSE)

if(length(samples) > 1){

tmp$value<-rowMeans(tpms[,samples])

}else{

tmp$value<-tpms[,samples]

}

tmp$factor<-f

tmp$gene<-rownames(tpms)

tmp$samples<-length(samples)

values<-rbind(values,tmp)

}

casted<-dcast(values, gene~factor, value.var="value")

casted$all<-NULL

rownames(casted)<-casted$gene

casted$gene<-NULL

casted<-as.matrix(casted)

tmp<-data.frame(value=numeric(nrow(casted)),stringsAsFactors=FALSE)

tmp$value<-rowMeans(casted)

tmp$factor<-"all_means"

tmp$gene<-rownames(casted)

tmp$samples<-ncol(casted)

values<-rbind(values,tmp)

triads_flat<-reshape_triad_groups(triads)

#print(nrow(values))

vals<-sqldf("SELECT `values`.*, chr_group, group_id FROM `values`

LEFT JOIN triads_flat on `values`.gene = triads_flat.gene")

vals

}

get_triad_details<-function(normalised_triad, factor="all",min_triad_sum=1){

tmp<-normalised_triad[factor==normalised_triad$factor,]

tmp<-tmp[tmp$triad_sum>min_triad_sum,]

norm_triad_mat<-get_triad_matrix(tmp, factor=factor)

centroid<-t(as.matrix(colMeans(norm_triad_mat,)))

dists<-rdist(norm_triad_mat,centroid)

rownames(dists)<-rownames(norm_triad_mat)

colnames(dists)<-c("Distance")

ranked_dists<-as.matrix(rank(dists)/length(dists))

rownames(ranked_dists)<-rownames(dists)

colnames(ranked_dists)<-c("P rank")

rank_df<-data.frame(dists,ranked_dists)

rank_df$group_id<-rownames(rank_df)

ranked_triads<-sqldf("SELECT tmp.*, Distance, `P.rank` FROM tmp NATURAL JOIN rank_df ")

t_centroid <- tableGrob(round(centroid*100,digits=2))

p_dist<-ggplot(data=rank_df, aes(Distance)) + geom_histogram(bins=100,aes(y =..density..)) +geom_density(col=2)

p_rank<-ggplot(rank_df,aes(Distance,P.rank))+ geom_bin2d(bins = 75)+theme(legend.position="bottom")

p_genome_dist<-plot_normalized_triads(tmp)

s<-sample(1:nrow(rank_df), 1500, replace=FALSE)

rank_s <- rank_df[s,]

title<-paste0("Genome contribution in triads: ", factor)

p<-arrangeGrob(p_genome_dist, p_dist, t_centroid, p_rank

, ncol=2, top = title)

list(distances=rank_df,triad=tmp ,centroid=centroid, p_dist=p_dist, dist_sample=rank_s,

p_rank=p_rank, p_genome_dist=p_genome_dist, plot=p, ranked_triads=ranked_triads, matrix=norm_triad_mat)

}

get_clusters_by_distance_and_plots<-function(normalized_triads,

factor="all",

output_prefix="",

min_triad_sum=1,

title="Triads"){

f<-factor

triad_test<-get_triad_details(normalized_triads,factor=factor,min_triad_sum=min_triad_sum)

triad<-triad_test$ranked_triads

test_mat<-triad_test$matrix

mat_df<-data.frame(test_mat)

mat_df$group_id<-rownames(test_mat)

title<-paste0(title, ": " , f)

centers<-t(matrix(c(0.33,0.33,0.33,1,0,0,0,1,0,0,0,1,0,0.5,0.5,0.5,0,0.5,0.5,0.5,0), nrow=3))

colnames(centers)<-c("A","B","D")

rownames(centers)<-c("Central","A.dominant","B.dominant","D.dominant","A.suppressed","B.suppressed","D.suppressed")

expectation_distance<-rdist(test_mat,centers)

colnames(expectation_distance)<-c("Central",

"A.dominant", "B.dominant", "D.dominant",

"A.suppressed","B.suppressed","D.suppressed")

rownames(expectation_distance)<-rownames(test_mat)

triad_test$expectation_distance<-expectation_distance

mins<-apply( expectation_distance, 1, which.min)

clust_desc<-colnames(expectation_distance)

name_mins<-clust_desc[mins]

general_desc<-c("Central","Dominant", "Dominant", "Dominant",

"Suppressed","Suppressed","Suppressed")

general_name_mins<-general_desc[mins]

mat_df$clust<-mins

mat_df$description<-name_mins

mat_df$general_description<-general_name_mins

mat_df<-cbind(mat_df,expectation_distance)

triad_test$triad<-mat_df

centroids<-get_centroids(mat_df)

centroids$description<-c("Central","A.dominant","B.dominant","D.dominant",

"A.suppressed","B.suppressed","D.suppressed"

)

total_genes<-sum(centroids$size)

centroids[8,"A"]<-triad_test$centroid[,"A"]

centroids[8,"B"]<-triad_test$centroid[,"B"]

centroids[8,"D"]<-triad_test$centroid[,"D"]

centroids[8,"description"] <- "Global"

centroids[8,"size"]<-total_genes

centroids[8,"clust"]<-8

centroids$factor<-factor

centroids$percentage<-centroids$size/total_genes

p<-plot_clust_dist(mat_df, title=title)

table_save<-sqldf("SELECT * FROM mat_df

NATURAL LEFT JOIN triad

ORDER BY triad.group_id, triad.chr_group")

table_save<-table_save[ , !(names(table_save) %in% c("A","B","D"))]

table_save$min_triad_sum<-min_triad_sum

triad_test$triad<-table_save

triad_test$centroids<-centroids

triad_test$plot_cluster<-p

centroids$samples<-mean(triad_test$triad$samples)

centroids$min_triad_sum<-min_triad_sum

if(length(output_prefix) > 0){

f2<-gsub("\n","-",f)

f2<-gsub("/","_",f)

p_filename<-paste0(output_prefix,"_",f2,"_triad_dist.pdf")

ggsave(p_filename,plot=triad_test$plot, width = 30, height = 25, units = c("cm"))

p_filename<-paste0(output_prefix,"_",f2,"_triad_cluster.pdf")

ggsave(p_filename,plot=p, width = 30, height = 25, units = c("cm"))

t_filename<-paste0(output_prefix,"_",f2,"_triad_cluster.txt")

write.table(table_save, file=t_filename,

sep="\t",quote=TRUE,row.names=FALSE, na="" )

t_filename<-paste0(output_prefix,"_",f2,"_triad_centroids.txt")

write.table(centroids, file=t_filename,

sep="\t",quote=TRUE,row.names=FALSE, na="")

}

triad_test

}

ks.central<-function(meanTPMS, factor="all"){

m<-meanTPMS[meanTPMS$factor==factor,c("description","chr_group","value")]

test_df<-NULL

for(gr in c("A","B","D")){

central<-m[m$description=="Central" & m$chr_group==gr, "value"]

for(d in unique(meanTPMS$description)){

compare<-m[m$description==d & m$chr_group==gr, "value"]

for(alt in c("less","greater")){

test<-ks.test(central, compare, alternative=alt)

test$chr_grp<-gr

test$compare<-d

test$alternative<-alt

tmp<-data.frame(t(unlist(test)),stringsAsFactors = FALSE)

if(is.null(test_df)){

test_df<-tmp

}else{

colnames(tmp)<-colnames(test_df)

test_df<-rbind(test_df,tmp)

}

}

}

}

#print(test_df$p.value)

test_df$fdr_by<-p.adjust(as.numeric(test_df$p.value),method="BY")

test_df$bonferroni<-p.adjust(as.numeric(test_df$p.value),method="bonferroni")

test_df$factor<-factor

test_df

}

plotMeansTPM<-function(meansTPM, title="Test", filename="test"){

m1<-meansTPM[meansTPM$factor=="all_mean_filter",]

m2<-meansTPM[meansTPM$factor!="all",]

m2<-m2[m2$factor!="all_means",]

m2<-m2[m2$factor!="all_mean_filter",]

p <- ggplot(m1, aes(description, value, fill=chr_group))

p <- p + geom_boxplot(outlier.alpha = 0.05)

p <- p + ylim(c(0, 50))

p <- p + theme(axis.text.x = element_text(angle = 90, hjust = 1),

legend.position="bottom")

p1 <- p + ylab("TPM") + xlab("Category")

p <- ggplot(m2, aes(description, value))

p <- p + geom_boxplot(outlier.alpha = 0.05)

p <- p + ylim(c(0, 50)) + theme(axis.text.x = element_text(angle = 90, hjust = 1))

p <- p + facet_wrap(~ factor, drop = TRUE)

p2 <- p + ylab("TPM") + xlab("Category")

p <- ggplot(m1, aes(description, value))

p <- p + geom_boxplot(outlier.alpha = 0.05)

p <- p + ylim(c(0, 50))

p <-p + theme(axis.text.x = element_text(angle = 90, hjust = 1),

legend.position="bottom")

p3 <- p + ylab("TPM") + xlab("")

p <- ggplot(m1, aes(general_description, value, fill=chr_group))

p <- p + geom_boxplot(outlier.alpha = 0.05)

p <- p + ylim(c(0, 50))

p <- p + theme(axis.text.x = element_text(angle = 90, hjust = 1),

legend.position="bottom")

p4 <- p + ylab("TPM") + xlab("Category")

lay <- rbind(c( 3,2,2,4),

c( 1,2,2,NA))

g2 <- arrangeGrob(p1,p2, p3,p4,layout_matrix = lay, top = title)

p_filename<-paste0(filename,"_TPM_by_triad_disribution.pdf")

ggsave(p_filename,plot=g2, width = 30, height = 25, units = c("cm"))

}

get_normalized_triad_from_clusters<-function(meansTPM, triads){

m2<-meansTPM[meansTPM$factor!="all",]

m2<-m2[m2$factor!="all_means",]

tpms<-dcast(m2, gene~factor, value.var="value",drop=FALSE, fill=NA)

rownames(tpms)<-tpms$gene

tpms$gene<-NULL

values<-data.frame(value=numeric(nrow(tpms)),stringsAsFactors=FALSE)

values$value<-rowMeans(tpms, na.rm = TRUE)

values$factor<-"all_mean_filter"

values$gene<-rownames(tpms)

values$samples<-rowSums(!is.na(tpms))

triads_flat<-reshape_triad_groups(triads)

means_group<-sqldf("SELECT `values`.*, chr_group, group_id FROM `values`

LEFT JOIN triads_flat on `values`.gene = triads_flat.gene")

sums_per_group<-sqldf("SELECT group_id , factor, sum(value) as triad_sum

FROM means_group GROUP BY group_id , factor")

normalized_triads<-sqldf("SELECT means_group.*, triad_sum, value/triad_sum as normalised_triad

FROM means_group LEFT JOIN sums_per_group

ON sums_per_group.group_id = means_group.group_id

AND sums_per_group.factor = means_group.factor ")

normalized_triads

}

get_triads_and_plots<-function(metadata,tpms, homologies, genes_to_use,

type="Intermed.tissue",

dataset="All",

folder=".\\Figures\\All_synteny_triads",

min_triad_sums=c(10),

min_mean_triad_sums=c(0.5) ){

triadas_with_genes<-sqldf("SELECT * from homologies

WHERE synteny='all_synteny'

AND

(A in genes_to_use

OR B in genes_to_use

OR D in genes_to_use )

")

print(head(homologies))

print(nrow(triadas_with_genes))

print(head(genes_to_use))

tpms_for_triads<-tpms[c(as.character(triadas_with_genes$A),

as.character(triadas_with_genes$B),

as.character(triadas_with_genes$D)),]

print(nrow(tpms_for_triads))

#print(head(tpms_for_triads))

means_group<-get_means_df(metadata, tpms_for_triads,triadas_with_genes, type=type)

#means_group<-get_means_df(metadata, tpms_for_triads,type=type, min_mean_tpm=0.5)

sums_per_group<-sqldf("SELECT group_id , factor, sum(value) as triad_sum

FROM means_group GROUP BY group_id , factor")

normalized_triads<-sqldf("SELECT means_group.*, triad_sum, value/triad_sum as normalised_triad

FROM means_group LEFT JOIN sums_per_group

ON sums_per_group.group_id = means_group.group_id

AND sums_per_group.factor = means_group.factor ")

path<-paste0(folder,"\\",dataset,"\\",type,"\\")

for(min_triad_sum in min_triad_sums){

local_triads<-NULL

ks_ret<-NULL

centroids<-NULL

triads<-NULL

path<-paste0(folder,"\\",dataset,"\\",type,"\\min_tpm_sum_",min_triad_sum,"\\")

path<-gsub(" ","_",path)

dir.create(path, showWarnings = TRUE, recursive = TRUE, mode = "0777")

path<-paste0(folder,"\\",dataset,"\\",type,"\\min_tpm_sum_",min_triad_sum,"\\min_tpm_sum_",min_triad_sum)

for(f in unique(normalized_triads$factor)){

title<-paste0(dataset," triads:" , f, ".\\nMinimum triad TPM sum: ", min_triad_sum)

clusters<-get_clusters_by_distance_and_plots(normalized_triads, factor=f, title=title,

min_triad_sum=min_triad_sum,

output_prefix=path)

suppressWarnings(

tmp<-ks.central(clusters$triad, factor=f)

)

if(is.null(centroids)){

centroids<-clusters$centroids

triads<-clusters$triad

ks_ret<-tmp

}else{

centroids<-rbind(centroids,clusters$centroids)

triads<-rbind(triads,clusters$triad)

ks_ret<-rbind(ks_ret, tmp)

}

}

normalized_triads_filter<-get_normalized_triad_from_clusters(triads,triadas_with_genes )

title<-paste0(dataset," triads:" , f, ".\\nMinimum triad TPM sum: ", min_triad_sum)

clusters<-get_clusters_by_distance_and_plots(normalized_triads_filter, factor="all_mean_filter",

title=title,

min_triad_sum=min_triad_sum,

output_prefix=path)

centroids<-rbind(centroids,clusters$centroids)

triads<-rbind(triads,clusters$triad)

suppressWarnings(

tmp<-ks.central(clusters$triad, factor="all_mean_filter")

)

ks_ret<-rbind(ks_ret, tmp)

t_filename<-paste0(path,"min_tpm_sum",min_triad_sum)

title<-paste0("Category distribution ", dataset, "\n", type, "\n", "Min TPM average sum: ", min_triad_sum)

plotMeansTPM(triads, filename=t_filename, title=title)

t_filename<-paste0(path, "min_tpm_sum_", min_triad_sum, "_ks.txt")

write.table(ks_ret, file=t_filename, sep="\t",quote=TRUE,row.names=FALSE, na="")

t_filename<-paste0(path,"summary_triad_centroids.txt")

write.table(centroids, file=t_filename, sep="\t",quote=TRUE,row.names=FALSE, na="")

t_filename<-paste0(path,"summary_triad_cluster.txt")

write.table(triads, file=t_filename, sep="\t",quote=TRUE,row.names=FALSE, na="")

}

}

nrow(metadata)

head(metadata)

head(genes_to_use)

head(tpms)

head(homologies)

#plots for Intermed.tissue

get_triads_and_plots(metadata,tpms,homologies,genes_to_use, type="Intermed.tissue",

dataset="WGCNA_samples" , min_triad_sum=c(0.5))

**#Part 11: Calculate homeolog expression patterns in triads (modified script).**

#This script was used to calculate homeologs expression patterns in the triads that are expressed in meiotic anther tissue.

#Triad was considered expressed when any of its homoeologs was expressed according to the criterion used in the WCGNA analysis.

library(sqldf)

library(ggplot2)

library(reshape2)

library(fields)

library(gridExtra)

library(ggtern)

library(clue)

library(geometry)

setwd("G:\\ABDUL_KADER\\Meiosis_genes_in_wheat\\Gene_Networks\\WGCNA_annoV1.1\\CalculateTriadCategory_Max_tpm_0.5\\")

metadata<-read.csv("metadata.txt", row.names = 1, sep="\t")

nrow(metadata)

head(metadata)

loadValuesFromExperiment<-function(metadata, folder, unit="tpm", values=c("Aneuploidy")){

metadata$Sample.IDs <- gsub("-",".",metadata$Sample.IDs)

v<-values[1]

v<-gsub(" ","_",v)

v<-gsub(",",".",v)

path<-paste0(folder,"\\",v,"_",unit,".tsv")

ret<-read.table(path, row.names = 1, header= TRUE)

if(length(values) > 1){

for(i in 2:length(values)){

v<-values[i]

v<-gsub(" ","_",v)

v<-gsub(",",".",v)

path<-paste0(folder,"\\",v,"_",unit,".tsv")

tmp<-read.table(path, row.names = 1, header= TRUE)

ret<-cbind(ret,tmp)

}

}

md<-metadata[metadata$Sample.IDs%in%colnames(ret),]

ret<-ret[,as.character(md$Sample.IDs),]

list(ret,md)

}

folder<-"expressionValuesPerGene"

tpms <-loadValuesFromExperiment(metadata, folder, unit="tpm", values=unique(metadata$study.title))

metadata_used<-tpms[[2]]

tpms<-tpms[[1]]

nrow(metadata_used)

#Loading the homologies

homologies<-read.csv("HCTriads.csv", sep=",")

head(homologies)

nrow(homologies)

expressed_genes<-read.csv("Expressed_genes_in_triads_per_Meiotic_anther.csv")

dim(expressed_genes)

head(expressed_genes)

genes_to_use <- data.frame(gene=expressed_genes$gene)

head(genes_to_use)

nrow(genes_to_use)

getSamplesForFactor<- function(metadata, type="Intermed.tissue",factor="Meiotic_anther"){

ret<-""

if(type != "all"){

ret<-as.character(metadata[metadata[,type] == factor,]$Sample.IDs)

}else{

ret<-as.character(unique(metadata$Sample.IDs))

}

ret

}

getMeansPerFactor<- function(values, metadata, type="Intermed.tissue",factor="Meiotic_anther"){

samples <- getSamplesForFactor(metadata, type, factor)

vals <- values[,samples]

mean<-0

if(length(samples) == 1){

print("This factor only has one sample!")

print(factor)

mean<-vals

}else{

mean<-rowMeans(vals)

}

mean<-sort(mean,decreasing=T)

cumulative <- cumsum(mean)

cumulative<-data.frame(cumulative)

mean<-data.frame(mean)

mean$gene <- rownames(mean)

cumulative$gene <- rownames(cumulative)

mean$total_samples <- length(samples)

n <-merge(mean,cumulative, by='gene', all=T)

n <- n[order(n$cumulative,decreasing = F),]

n$seq <- seq(from = 1, to = nrow(n))

n$factor = factor

n

}

getMeansForAllFactors<-function(values, metadata, type="Intermed.tissue"){

factors<-unique(metadata[,type])

f<-factors[1]

meansDFs <- getMeansPerFactor(tpms,metadata,type=type, factor=f)

for (i in 2:length(factors)){

f<-factors[i]

localDF<-getMeansPerFactor(tpms,metadata,type=type, factor=f)

meansDFs <- rbind(meansDFs,localDF)

}

meansDFs

}

isExpressedPerFactor <- function(values, metadata, type="Intermed.tissue",factor="Meiotic_anther", minTPM=0){

samples <- getSamplesForFactor(metadata, type, factor)

vals <- values[,samples]

means <- rowMeans(vals)

expr <- means > minTPM

m2 <- data.frame( expressed = expr)

m2$factor<-factor

m2$transcript<-rownames(m2)

m2$total_samples <- length(samples)

m2

}

getExclusiveExpression<-function(values, metadata, minTPM=0, type="Intermed.tissue"){

means <- getMeansForAllFactors(values, metadata,type=type)

means$expressed<-means$mean > minTPM

exclusiveExpresison<-sqldf("SELECT gene, factor, mean, total_samples

FROM means

WHERE expressed

GROUP BY gene HAVING count(factor) = 1 ")

list(means, exclusiveExpresison )

}

reshape_triad_groups<-function(triads){

values<-data.frame(gene=triads$A, chr_group="A", group_id=triads$group_id)

values<-rbind(values,data.frame(gene=triads$B, chr_group="B", group_id=triads$group_id))

values<-rbind(values,data.frame(gene=triads$D, chr_group="D", group_id=triads$group_id))

values

}

get_triad_matrix<-function(normalised_triad, factor="all"){

tmp<-normalised_triad[factor==normalised_triad$factor,]

tmp<-acast(tmp, group_id~chr_group, value.var="normalised_triad")

tmp<-tmp[complete.cases(tmp),]

tmp

}

plot_normalized_triads<-function(triads){

p <- ggplot(triads, aes(chr_group, normalised_triad))

p <- p + geom_boxplot(outlier.alpha = 0.05)

p <- p + ylab("Contribution") + xlab("Chromosome group")

p

}

get_centroids<-function(clust_df){

clusters<-sort(unique(clust_df$clust))

n_clust<-length(clusters)

dat <- data.frame(

A=numeric(n_clust),B=numeric(n_clust), D=numeric(n_clust), size=numeric(n_clust),

clust=numeric(n_clust), description=character(n_clust),stringsAsFactors=FALSE )

for(c in clusters){

tmp_df_clust<-clust_df[clust_df$clust==c,]

dat[c,1] <- mean(tmp_df_clust$A)

dat[c,2] <- mean(tmp_df_clust$B)

dat[c,3] <- mean(tmp_df_clust$D)

dat[c,4] <- nrow(tmp_df_clust)

dat[c,5] <- c

dat[c,6] <- paste0("other.",c)

}

rownames(dat)<-clusters

dat

}

plot_clust_dist<-function(clust_df, title="All"){

tmp_df<-clust_df[,c("A","B","D","group_id","clust","description")]

tmp_df<-melt(tmp_df,id=c("group_id","clust","description"))

colnames(tmp_df)<-c("group_id","clust","description","chr_group","normalised_triad")

clusters<-sort(unique(tmp_df$description))

tern <- ggtern(clust_df,aes(A,B,D,color=description, alpha=0.01)) +

geom_point() + theme_legend_position(x = "topleft") + scale_alpha(guide = 'none')

gs<-list(tern)

dat <- data.frame(

A=numeric(0),B=numeric(0), D=numeric(0), size=numeric(0),stringsAsFactors=FALSE )

rownames(dat)<-rownames(clusters)

for(c in clusters){

tmp_df_clust<-tmp_df[tmp_df$description==c,]

p<-plot_normalized_triads(tmp_df_clust)

p<- p + ylim(0,1)

p<- p + ylab("") + xlab("")

p<- p + ggtitle(c)

dat[c,1] <- round(100*mean(tmp_df_clust[tmp_df_clust$chr_group=="A","normalised_triad"]),digits=2)

dat[c,2] <- round(100*mean(tmp_df_clust[tmp_df_clust$chr_group=="B","normalised_triad"]),digits=2)

dat[c,3] <- round(100*mean(tmp_df_clust[tmp_df_clust$chr_group=="D","normalised_triad"]),digits=2)

dat[c,4] <- nrow(tmp_df_clust)

gs[[length(gs)+1]] <- p

}

total_size<-sum(dat$size)

dat$percentage<-round(100*dat$size/total_size,digits=2)

gs[[length(gs)+1]]<-tableGrob(dat)

lay <- rbind(c( 1, 1, 1, 2, 4, 7),

c( 1, 1, 1, 3, 5, 8),

c( 9, 9, 9, 6,NA,NA)

)

g2 <- arrangeGrob(grobs = gs, layout_matrix = lay, top = title)

g2

}

get_means_df<-function(metadata, tpms, type="Intermed.tissue", min_mean_tpm=0){

samples<-getSamplesForFactor(metadata, type="all",factor="all")

values<-data.frame(value=numeric(nrow(tpms)),stringsAsFactors=FALSE)

if(length(samples) > 1){

print("All samples:")

#print(samples)

#print(colnames(tpms))

values$value<-rowMeans(tpms[,samples])

}else{

values$value<-tpms[,samples]

}

values$factor<-"all"

values$gene<-rownames(tpms)

values$samples<-length(samples)

print(unique(metadata[,type]))

for(f in as.character(unique(metadata[,type]))){

print(f)

samples<-getSamplesForFactor(metadata, type=type,factor=f)

tmp<-data.frame(value=numeric(nrow(tpms)),stringsAsFactors=FALSE)

if(length(samples) > 1){

tmp$value<-rowMeans(tpms[,samples])

}else{

tmp$value<-tpms[,samples]

}

tmp$factor<-f

tmp$gene<-rownames(tpms)

tmp$samples<-length(samples)

print(colnames(values))

print(colnames(tmp))

values<-rbind(values,tmp)

}

casted<-dcast(values, gene~factor, value.var="value")

casted$all<-NULL

rownames(casted)<-casted$gene

casted$gene<-NULL

casted<-as.matrix(casted)

print(head(casted))

tmp<-data.frame(value=numeric(nrow(casted)),stringsAsFactors=FALSE)

tmp$value<-rowMeans(casted)

tmp$factor<-"all_means"

tmp$gene<-rownames(casted)

tmp$samples<-ncol(casted)

print("...")

print(colnames(values))

print(colnames(tmp))

values<-rbind(values,tmp)

print(head(casted))

print(head(min_mean_tpm))

casted<-ifelse(casted < min_mean_tpm, NA, casted)

print(head(casted))

tmp<-data.frame(value=numeric(nrow(casted)),stringsAsFactors=FALSE)

tmp$value<-rowMeans(casted, na.rm = TRUE)

tmp$factor<-"all_mean_filter"

tmp$gene<-rownames(casted)

tmp$samples<-rowSums(!is.na(casted))

print("...")

print(colnames(values))

print(colnames(tmp))

values<-rbind(values,tmp)

values

}

get_means_df<-function(metadata, tpms, triads, type="Intermed.tissue"){

samples<-getSamplesForFactor(metadata, type="all",factor="all")

values<-data.frame(value=numeric(nrow(tpms)),stringsAsFactors=FALSE)

if(length(samples) > 1){

values$value<-rowMeans(tpms[,samples])

}else{

values$value<-tpms[,samples]

}

values$factor<-"all"

values$gene<-rownames(tpms)

values$samples<-length(samples)

for(f in unique(metadata[,type])){

samples<-getSamplesForFactor(metadata, type=type,factor=f)

tmp<-data.frame(value=numeric(nrow(tpms)),stringsAsFactors=FALSE)

if(length(samples) > 1){

tmp$value<-rowMeans(tpms[,samples])

}else{

tmp$value<-tpms[,samples]

}

tmp$factor<-f

tmp$gene<-rownames(tpms)

tmp$samples<-length(samples)

values<-rbind(values,tmp)

}

casted<-dcast(values, gene~factor, value.var="value")

casted$all<-NULL

rownames(casted)<-casted$gene

casted$gene<-NULL

casted<-as.matrix(casted)

tmp<-data.frame(value=numeric(nrow(casted)),stringsAsFactors=FALSE)

tmp$value<-rowMeans(casted)

tmp$factor<-"all_means"

tmp$gene<-rownames(casted)

tmp$samples<-ncol(casted)

values<-rbind(values,tmp)

triads_flat<-reshape_triad_groups(triads)

#print(nrow(values))

vals<-sqldf("SELECT `values`.*, chr_group, group_id FROM `values`

LEFT JOIN triads_flat on `values`.gene = triads_flat.gene")

vals

}

get_triad_details<-function(normalised_triad, factor="all",min_triad_sum=0){

tmp<-normalised_triad[factor==normalised_triad$factor,]

tmp<-tmp[tmp$triad_sum>min_triad_sum,]

norm_triad_mat<-get_triad_matrix(tmp, factor=factor)

centroid<-t(as.matrix(colMeans(norm_triad_mat,)))

dists<-rdist(norm_triad_mat,centroid)

rownames(dists)<-rownames(norm_triad_mat)

colnames(dists)<-c("Distance")

ranked_dists<-as.matrix(rank(dists)/length(dists))

rownames(ranked_dists)<-rownames(dists)

colnames(ranked_dists)<-c("P rank")

rank_df<-data.frame(dists,ranked_dists)

rank_df$group_id<-rownames(rank_df)

ranked_triads<-sqldf("SELECT tmp.*, Distance, `P.rank` FROM tmp NATURAL JOIN rank_df ")

t_centroid <- tableGrob(round(centroid*100,digits=2))

p_dist<-ggplot(data=rank_df, aes(Distance)) + geom_histogram(bins=100,aes(y =..density..)) +geom_density(col=2)

p_rank<-ggplot(rank_df,aes(Distance,P.rank))+ geom_bin2d(bins = 75)+theme(legend.position="bottom")

p_genome_dist<-plot_normalized_triads(tmp)

s<-sample(1:nrow(rank_df), 1500, replace=FALSE)

rank_s <- rank_df[s,]

title<-paste0("Genome contribution in triads: ", factor)

p<-arrangeGrob(p_genome_dist, p_dist, t_centroid, p_rank

, ncol=2, top = title)

list(distances=rank_df,triad=tmp ,centroid=centroid, p_dist=p_dist, dist_sample=rank_s,

p_rank=p_rank, p_genome_dist=p_genome_dist, plot=p, ranked_triads=ranked_triads, matrix=norm_triad_mat)

}

get_clusters_by_distance_and_plots<-function(normalized_triads,

factor="all",

output_prefix="",

min_triad_sum=0,

title="Triads"){

f<-factor

triad_test<-get_triad_details(normalized_triads,factor=factor,min_triad_sum=min_triad_sum)

triad<-triad_test$ranked_triads

test_mat<-triad_test$matrix

mat_df<-data.frame(test_mat)

mat_df$group_id<-rownames(test_mat)

title<-paste0(title, ": " , f)

centers<-t(matrix(c(0.33,0.33,0.33,1,0,0,0,1,0,0,0,1,0,0.5,0.5,0.5,0,0.5,0.5,0.5,0), nrow=3))

colnames(centers)<-c("A","B","D")

rownames(centers)<-c("Central","A.dominant","B.dominant","D.dominant","A.suppressed","B.suppressed","D.suppressed")

expectation_distance<-rdist(test_mat,centers)

colnames(expectation_distance)<-c("Central",

"A.dominant", "B.dominant", "D.dominant",

"A.suppressed","B.suppressed","D.suppressed")

rownames(expectation_distance)<-rownames(test_mat)

triad_test$expectation_distance<-expectation_distance

mins<-apply( expectation_distance, 1, which.min)

clust_desc<-colnames(expectation_distance)

name_mins<-clust_desc[mins]

general_desc<-c("Central","Dominant", "Dominant", "Dominant",

"Suppressed","Suppressed","Suppressed")

general_name_mins<-general_desc[mins]

mat_df$clust<-mins

mat_df$description<-name_mins

mat_df$general_description<-general_name_mins

mat_df<-cbind(mat_df,expectation_distance)

triad_test$triad<-mat_df

centroids<-get_centroids(mat_df)

centroids$description<-c("Central","A.dominant","B.dominant","D.dominant",

"A.suppressed","B.suppressed","D.suppressed"

)

total_genes<-sum(centroids$size)

centroids[8,"A"]<-triad_test$centroid[,"A"]

centroids[8,"B"]<-triad_test$centroid[,"B"]

centroids[8,"D"]<-triad_test$centroid[,"D"]

centroids[8,"description"] <- "Global"

centroids[8,"size"]<-total_genes

centroids[8,"clust"]<-8

centroids$factor<-factor

centroids$percentage<-centroids$size/total_genes

p<-plot_clust_dist(mat_df, title=title)

table_save<-sqldf("SELECT * FROM mat_df

NATURAL LEFT JOIN triad

ORDER BY triad.group_id, triad.chr_group")

table_save<-table_save[ , !(names(table_save) %in% c("A","B","D"))]

table_save$min_triad_sum<-min_triad_sum

triad_test$triad<-table_save

triad_test$centroids<-centroids

triad_test$plot_cluster<-p

centroids$samples<-mean(triad_test$triad$samples)

centroids$min_triad_sum<-min_triad_sum

if(length(output_prefix) > 0){

f2<-gsub("\n","-",f)

f2<-gsub("/","_",f)

p_filename<-paste0(output_prefix,"_",f2,"_triad_dist.pdf")

ggsave(p_filename,plot=triad_test$plot, width = 30, height = 25, units = c("cm"))

p_filename<-paste0(output_prefix,"_",f2,"_triad_cluster.pdf")

ggsave(p_filename,plot=p, width = 30, height = 25, units = c("cm"))

t_filename<-paste0(output_prefix,"_",f2,"_triad_cluster.txt")

write.table(table_save, file=t_filename,

sep="\t",quote=TRUE,row.names=FALSE, na="" )

t_filename<-paste0(output_prefix,"_",f2,"_triad_centroids.txt")

write.table(centroids, file=t_filename,

sep="\t",quote=TRUE,row.names=FALSE, na="")

}

triad_test

}

ks.central<-function(meanTPMS, factor="all"){

m<-meanTPMS[meanTPMS$factor==factor,c("description","chr_group","value")]

test_df<-NULL

for(gr in c("A","B","D")){

central<-m[m$description=="Central" & m$chr_group==gr, "value"]

for(d in unique(meanTPMS$description)){

compare<-m[m$description==d & m$chr_group==gr, "value"]

for(alt in c("less","greater")){

test<-ks.test(central, compare, alternative=alt)

test$chr_grp<-gr

test$compare<-d

test$alternative<-alt

tmp<-data.frame(t(unlist(test)),stringsAsFactors = FALSE)

if(is.null(test_df)){

test_df<-tmp

}else{

colnames(tmp)<-colnames(test_df)

test_df<-rbind(test_df,tmp)

}

}

}

}

#print(test_df$p.value)

test_df$fdr_by<-p.adjust(as.numeric(test_df$p.value),method="BY")

test_df$bonferroni<-p.adjust(as.numeric(test_df$p.value),method="bonferroni")

test_df$factor<-factor

test_df

}

plotMeansTPM<-function(meansTPM, title="Test", filename="test"){

m1<-meansTPM[meansTPM$factor=="all_mean_filter",]

m2<-meansTPM[meansTPM$factor!="all",]

m2<-m2[m2$factor!="all_means",]

m2<-m2[m2$factor!="all_mean_filter",]

p <- ggplot(m1, aes(description, value, fill=chr_group))

p <- p + geom_boxplot(outlier.alpha = 0.05)

p <- p + ylim(c(0, 50))

p <- p + theme(axis.text.x = element_text(angle = 90, hjust = 1),

legend.position="bottom")

p1 <- p + ylab("TPM") + xlab("Category")

p <- ggplot(m2, aes(description, value))

p <- p + geom_boxplot(outlier.alpha = 0.05)

p <- p + ylim(c(0, 50)) + theme(axis.text.x = element_text(angle = 90, hjust = 1))

p <- p + facet_wrap(~ factor, drop = TRUE)

p2 <- p + ylab("TPM") + xlab("Category")

p <- ggplot(m1, aes(description, value))

p <- p + geom_boxplot(outlier.alpha = 0.05)

p <- p + ylim(c(0, 50))

p <-p + theme(axis.text.x = element_text(angle = 90, hjust = 1),

legend.position="bottom")

p3 <- p + ylab("TPM") + xlab("")

p <- ggplot(m1, aes(general_description, value, fill=chr_group))

p <- p + geom_boxplot(outlier.alpha = 0.05)

p <- p + ylim(c(0, 50))

p <- p + theme(axis.text.x = element_text(angle = 90, hjust = 1),

legend.position="bottom")

p4 <- p + ylab("TPM") + xlab("Category")

lay <- rbind(c( 3,2,2,4),

c( 1,2,2,NA))

g2 <- arrangeGrob(p1,p2, p3,p4,layout_matrix = lay, top = title)

p_filename<-paste0(filename,"_TPM_by_triad_disribution.pdf")

ggsave(p_filename,plot=g2, width = 30, height = 25, units = c("cm"))

}

get_normalized_triad_from_clusters<-function(meansTPM, triads){

m2<-meansTPM[meansTPM$factor!="all",]

m2<-m2[m2$factor!="all_means",]

tpms<-dcast(m2, gene~factor, value.var="value",drop=FALSE, fill=NA)

rownames(tpms)<-tpms$gene

tpms$gene<-NULL

values<-data.frame(value=numeric(nrow(tpms)),stringsAsFactors=FALSE)

values$value<-rowMeans(tpms, na.rm = TRUE)

values$factor<-"all_mean_filter"

values$gene<-rownames(tpms)

values$samples<-rowSums(!is.na(tpms))

triads_flat<-reshape_triad_groups(triads)

means_group<-sqldf("SELECT `values`.*, chr_group, group_id FROM `values`

LEFT JOIN triads_flat on `values`.gene = triads_flat.gene")

sums_per_group<-sqldf("SELECT group_id , factor, sum(value) as triad_sum

FROM means_group GROUP BY group_id , factor")

normalized_triads<-sqldf("SELECT means_group.*, triad_sum, value/triad_sum as normalised_triad

FROM means_group LEFT JOIN sums_per_group

ON sums_per_group.group_id = means_group.group_id

AND sums_per_group.factor = means_group.factor ")

normalized_triads

}

get_triads_and_plots<-function(metadata,tpms, homologies, genes_to_use,

type="Intermed.tissue",

dataset="All",

folder=".\\Figures",

min_triad_sums=c(0),

min_mean_triad_sums=c(0) ){

triadas_with_genes<-sqldf("SELECT * from homologies

WHERE synteny='all_synteny'

AND

(A in genes_to_use

OR B in genes_to_use

OR D in genes_to_use )

")

print(head(homologies))

print(nrow(triadas_with_genes))

print(head(genes_to_use))

tpms_for_triads<-tpms[c(as.character(triadas_with_genes$A),

as.character(triadas_with_genes$B),

as.character(triadas_with_genes$D)),]

print(nrow(tpms_for_triads))

means_group<-get_means_df(metadata, tpms_for_triads,triadas_with_genes, type=type)

sums_per_group<-sqldf("SELECT group_id , factor, sum(value) as triad_sum

FROM means_group GROUP BY group_id , factor")

normalized_triads<-sqldf("SELECT means_group.*, triad_sum, value/triad_sum as normalised_triad

FROM means_group LEFT JOIN sums_per_group

ON sums_per_group.group_id = means_group.group_id

AND sums_per_group.factor = means_group.factor ")

path<-paste0(folder,"\\",dataset,"\\",type,"\\")

for(min_triad_sum in min_triad_sums){

local_triads<-NULL

ks_ret<-NULL

centroids<-NULL

triads<-NULL

path<-paste0(folder,"\\",dataset,"\\",type,"\\min_tpm_sum_",min_triad_sum,"\\")

path<-gsub(" ","_",path)

dir.create(path, showWarnings = TRUE, recursive = TRUE, mode = "0777")

path<-paste0(folder,"\\",dataset,"\\",type,"\\min_tpm_sum_",min_triad_sum,"\\min_tpm_sum_",min_triad_sum)

for(f in unique(normalized_triads$factor)){

title<-paste0(dataset," triads:" , f, ".\\nMinimum triad TPM sum: ", min_triad_sum)

clusters<-get_clusters_by_distance_and_plots(normalized_triads, factor=f, title=title,

min_triad_sum=min_triad_sum,

output_prefix=path)

suppressWarnings(

tmp<-ks.central(clusters$triad, factor=f)

)

if(is.null(centroids)){

centroids<-clusters$centroids

triads<-clusters$triad

ks_ret<-tmp

}else{

centroids<-rbind(centroids,clusters$centroids)

triads<-rbind(triads,clusters$triad)

ks_ret<-rbind(ks_ret, tmp)

}

}

normalized_triads_filter<-get_normalized_triad_from_clusters(triads,triadas_with_genes )

title<-paste0(dataset," triads:" , f, ".\\nMinimum triad TPM sum: ", min_triad_sum)

clusters<-get_clusters_by_distance_and_plots(normalized_triads_filter, factor="all_mean_filter",

title=title,

min_triad_sum=min_triad_sum,

output_prefix=path)

centroids<-rbind(centroids,clusters$centroids)

triads<-rbind(triads,clusters$triad)

suppressWarnings(

tmp<-ks.central(clusters$triad, factor="all_mean_filter")

)

ks_ret<-rbind(ks_ret, tmp)

t_filename<-paste0(path,"min_tpm_sum",min_triad_sum)

title<-paste0("Category distribution ", dataset, "\n", type, "\n", "Min TPM average sum: ", min_triad_sum)

plotMeansTPM(triads, filename=t_filename, title=title)

t_filename<-paste0(path, "min_tpm_sum_", min_triad_sum, "_ks.txt")

write.table(ks_ret, file=t_filename, sep="\t",quote=TRUE,row.names=FALSE, na="")

t_filename<-paste0(path,"summary_triad_centroids.txt")

write.table(centroids, file=t_filename, sep="\t",quote=TRUE,row.names=FALSE, na="")

t_filename<-paste0(path,"summary_triad_cluster.txt")

write.table(triads, file=t_filename, sep="\t",quote=TRUE,row.names=FALSE, na="")

}

}

#plots for Intermed.tissue

get_triads_and_plots(metadata,tpms,homologies,genes_to_use, type="Intermed.tissue",

dataset="MeioticAntherTriads" , min_triad_sum=c(0))

**#Part 12: Export meiosis-related modules data for Cytoscape visualisation.**

setwd("G:\\ABDUL_KADER\\Meiosis_genes_in_wheat\\Gene_Networks\\WGCNA_annoV1.1\\HC_exp_in_meiosis\\maxP0.05")

library(WGCNA)

options(stringsAsFactors = FALSE)

lnames=load(file="G:\\ABDUL_KADER\\Meiosis_genes_in_wheat\\Gene_Networks\\WGCNA_annoV1.1\\HC_exp_in_meiosis\\filtered_data_HC_only_ready_for_WGCNA_0.5tpm.Rdata")

lnames

datExpr <- datExpr0

rm(datExpr0)

head(rownames(datExpr))

head(colnames(datExpr))

lnames=load(file="G:\\ABDUL_KADER\\Meiosis_genes_in_wheat\\Gene_Networks\\WGCNA_annoV1.1\\HC_exp_in_meiosis\\maxP0.05\\bwnet_network_components_mergeCutHeight0.15.Rdata")

lnames

#find out which genes were in which blocks

bwnet_test <- load(file="bwnet_network_mergeCutHeight0.15.RData")

names(bwnet)

is.vector(bwnet$blocks) #[1] TRUE

names(bwnet$blocks) #NULL

blocks_df <- as.data.frame(bwnet$blocks)

head(blocks_df)

rownames(blocks_df) <- colnames(datExpr)

head(blocks_df)

write.csv(file="blocks.csv", blocks_df)

# now find out which blocks contained which modules (do this by comparing gene content)

gene_module <- read.csv(file="genes_with_modules_mergeCutHeight0.15_no_expr_values.csv", header=T)

head(gene_module)

merged_gene_module_block <- merge(gene_module, blocks_df, by.x="X", by.y=0)

head(merged_gene_module_block)

tail(merged_gene_module_block)

write.csv(file="merged_gene_module_block.csv", merged_gene_module_block)

blocks_modules <- merged_gene_module_block[,2:4]

head(blocks_modules)

blocks_modules <- blocks_modules[!duplicated(blocks_modules),]

#For module 2

setwd("G:\\ABDUL_KADER\\Meiosis_genes_in_wheat\\Gene_Networks\\WGCNA_annoV1.1\\HC_exp_in_meiosis\\maxP0.05")

modules <- c("2")

modules #[1] "2"

# which block is this module in?

block <- blocks_modules[blocks_modules$bwnetModuleLabels==modules,][,3]

block #[1] 2

block[1] #[1] 2

# load 2nd TOM

lnames <- load(paste0("Signed_hybrid_TOM-blockwise_maxP0.05-block.",block,".Rdata"))

lnames #[1] "TOM"

TOM.mat = as.matrix(TOM)

# genes in block

genes_in_block <- merged_gene_module_block[merged_gene_module_block$`bwnet$blocks`==block,]

head(genes_in_block)

head(genes_in_block$bwnetModuleLabels)

dim(genes_in_block)

# Select module probes

probes = genes_in_block$X

head(probes)

inModule = is.finite(match(genes_in_block$bwnetModuleLabels, modules));

head(inModule)

inModule

modProbes = probes[inModule];

head(modProbes)

length(probes)

length(modProbes) #[1] 4940 (this is the number of genes in the selected module.. ME2)

# Select the corresponding Topological Overlap

dim(TOM.mat)

modTOM = TOM.mat[inModule, inModule];

dimnames(modTOM) = list(modProbes, modProbes)

dim(modTOM)

setwd("G:\\ABDUL_KADER\\Meiosis_genes_in_wheat\\Gene_Networks\\WGCNA_annoV1.1\\HC_exp_in_meiosis\\maxP0.05\\Cytoscape")

threshold <- 0.25 #use different thresholds (0.1, 0.15, 0.2, 0.05, 0.02, 0.01)

cyt = exportNetworkToCytoscape(modTOM,

edgeFile = paste("CytoscapeInput-edges-", paste(modules, collapse="-"),"threshold", threshold, ".txt", sep=""),

nodeFile = paste("CytoscapeInput-nodes-", paste(modules, collapse="-"),"threshold", threshold, ".txt", sep=""),

weighted = TRUE,

threshold = threshold,

nodeNames = modProbes,

nodeAttr = genes_in_block$bwnetModuleLabels[inModule])

# For module 28

setwd("G:\\ABDUL_KADER\\Meiosis_genes_in_wheat\\Gene_Networks\\WGCNA_annoV1.1\\HC_exp_in_meiosis\\maxP0.05")

# choose the module and therefore the block to load

modules <- c("28")

modules

# which block is this module in?

block <- blocks_modules[blocks_modules$bwnetModuleLabels==modules,][,3]

block #[1] 1

block[1] #[1] 1

# load 2nd TOM

lnames <- load(paste0("Signed_hybrid_TOM-blockwise_maxP0.05-block.",block,".Rdata"))

lnames #[1] "TOM"

TOM.mat = as.matrix(TOM)

# genes in block

genes_in_block <- merged_gene_module_block[merged_gene_module_block$`bwnet$blocks`==block,]

head(genes_in_block)

head(genes_in_block$bwnetModuleLabels)

dim(genes_in_block)

# Select module probes

probes = genes_in_block$X

head(probes)

inModule = is.finite(match(genes_in_block$bwnetModuleLabels, modules));

head(inModule)

modProbes = probes[inModule];

head(modProbes)

length(probes)

length(modProbes) #[1] 544 (this is the number of genes in the selected module.. ME11)

# Select the corresponding Topological Overlap

dim(TOM.mat)

modTOM = TOM.mat[inModule, inModule];

dimnames(modTOM) = list(modProbes, modProbes)

dim(modTOM)

setwd("G:\\ABDUL_KADER\\Meiosis_genes_in_wheat\\Gene_Networks\\WGCNA_annoV1.1\\HC_exp_in_meiosis\\maxP0.05\\Cytoscape")

threshold <- 0.15 #use different thresholds (0.1, 0.15, 0.2, 0.05, 0.02, 0.01)

cyt = exportNetworkToCytoscape(modTOM,

edgeFile = paste("CytoscapeInput-edges-", paste(modules, collapse="-"),"threshold", threshold, ".txt", sep=""),

nodeFile = paste("CytoscapeInput-nodes-", paste(modules, collapse="-"),"threshold", threshold, ".txt", sep=""),

weighted = TRUE,

threshold = threshold,

nodeNames = modProbes,

nodeAttr = genes_in_block$bwnetModuleLabels[inModule])

# For module 41

setwd("G:\\ABDUL_KADER\\Meiosis_genes_in_wheat\\Gene_Networks\\WGCNA_annoV1.1\\HC_exp_in_meiosis\\maxP0.05")

# choose the module and therefore the block to load

modules <- c("41")

modules

# which block is this module in?

block <- blocks_modules[blocks_modules$bwnetModuleLabels==modules,][,3]

block #[1] 2

block[1] #[1] 2

# load 2nd TOM

lnames <- load(paste0("Signed_hybrid_TOM-blockwise_maxP0.05-block.",block,".Rdata"))

lnames #[1] "TOM"

TOM.mat = as.matrix(TOM)

# genes in block

genes_in_block <- merged_gene_module_block[merged_gene_module_block$`bwnet$blocks`==block,]

head(genes_in_block)

head(genes_in_block$bwnetModuleLabels)

dim(genes_in_block) # [1] 25085 4

# Select module probes

probes = genes_in_block$X

head(probes)

inModule = is.finite(match(genes_in_block$bwnetModuleLabels, modules));

head(inModule) # [1] FALSE FALSE FALSE FALSE FALSE FALSE

modProbes = probes[inModule];

head(modProbes)

length(probes) #[1] 25085

length(modProbes) #[1] 313 (this is the number of genes in the selected module.. ME11)

# Select the corresponding Topological Overlap

dim(TOM.mat)

modTOM = TOM.mat[inModule, inModule];

dimnames(modTOM) = list(modProbes, modProbes)

dim(modTOM)

setwd("G:\\ABDUL_KADER\\Meiosis_genes_in_wheat\\Gene_Networks\\WGCNA_annoV1.1\\HC_exp_in_meiosis\\maxP0.05\\Cytoscape")

threshold <- 0.0 #use different thresholds (0.1, 0.15, 0.2, 0.05, 0.02, 0.01)

cyt = exportNetworkToCytoscape(modTOM,

edgeFile = paste("CytoscapeInput-edges-", paste(modules, collapse="-"),"threshold", threshold, ".txt", sep=""),

nodeFile = paste("CytoscapeInput-nodes-", paste(modules, collapse="-"),"threshold", threshold, ".txt", sep=""),

weighted = TRUE,

threshold = threshold,

nodeNames = modProbes,

nodeAttr = genes_in_block$bwnetModuleLabels[inModule])
